# From Far West to East: Joining the Molecular Architecture of Imidazole-like Ligands in HO-1 Complexes

**DOI:** 10.3390/ph14121289

**Published:** 2021-12-10

**Authors:** Giuseppe Floresta, Antonino Nicolò Fallica, Vincenzo Patamia, Valeria Sorrenti, Khaled Greish, Antonio Rescifina, Valeria Pittalà

**Affiliations:** 1 Department of Drug and Health Sciences, University of Catania, V.le A. Doria 6, 95125 Catania, Italy; giuseppe.floresta@kcl.ac.uk (G.F.); antonino.fallica@phd.unict.it (A.N.F.); vincenzo.patamia@unict.it (V.P.); sorrenti@unict.it (V.S.); 2 Department of Analytics, Environmental & Forensics, King’s College London, London SE1 9NH, UK; 3 Department of Molecular Medicine and Nanomedicine Unit, Princess Al-Jawhara Center for Molecular Medicine, College of Medicine and Medical Sciences, Arabian Gulf University, Manama 329, Bahrain; khaledfg@agu.edu.bh

**Keywords:** fragment-based ligand design, fragment growing, ligand joining, structure-based drug design, heme oxygenase, HO-1 inhibitors, imidazole

## Abstract

HO-1 overexpression has been reported in several cases/types of human malignancies. Unfortunately, poor clinical outcomes are reported in most of these cases, and the inhibition of HO-1 is considered a valuable and proven anticancer approach. To identify novel hit compounds suitable as HO-1 inhibitors, we report here a fragment-based approach where ligand joining experiments were used. The two most important parts of the classical structure of the HO-1 inhibitors were used as a starting point, and 1000 novel compounds were generated and then virtually evaluated by structure and ligand-based approaches. The joining experiments led us to a novel series of indole-based compounds. A synthetic pathway for eight selected molecules was designed, and the compounds were synthesized. The biological activity revealed that some molecules reach the micromolar activity, whereas molecule **4d** inhibits the HO-1 with an IC_50_ of 1.03 μM. This study suggested that our joining approach was successful, and a novel hit compound was generated. These results are ongoing for further development.

## 1. Introduction

Despite the several efforts accomplished by the scientific community, cancer still represents one of the most important causes of death worldwide. This medical condition represents a challenge from both a clinical and an economic point of view, and its treatment is far from being resolutive [1]. Modulating the activity of novel biological targets implicated in tumor onset and progression is regarded as a valuable strategy to define novel and better antitumor therapies [2]. In this context, it has been widely reported that heme oxygenase (HO) plays a key role in tumor growth and aggressiveness [3,4,5,6]. Heme oxygenase’s physiological role consists in the oxidative heme breakdown at the cytoplasmatic level with the consequent stoichiometric production of three catabolites: carbon monoxide, ferrous ion, and biliverdin [7]. After its production, biliverdin is later converted into bilirubin by the biliverdin reductase enzyme. Heme end-products are involved in the control of several physiological activities, ranging from antioxidant to neuroprotective functions [8,9,10,11,12]. Nowadays, two main HO isoforms have been described. Both isoforms possess a similar aminoacidic sequence and catalyze the same reaction but differ in their genetic origin, tissue distribution, and mechanism of induction [13]. HO-1, also referred to as heat-shock protein 32, is basally expressed in high concentrations in the spleen and the liver. In other tissues, its expression is triggered by stressful stimuli such as xenobiotics, toxins, heavy metals, UV radiations, and oxidative stress.

On the other hand, HO-2 represents the constitutive isoform generally expressed in the brain and testis, where it seems to play a role in vasodilation through carbon monoxide production, neuroprotection, and male reproduction [14,15]. Consistently with its overall cytoprotective properties, HO-1 is highly induced in cancerous cells [16,17,18,19]. Indeed, its overexpression has been linked to increased tumor aggressiveness, neovascularization, metastasization, failure of apoptosis, and poor chances of survival [20,21]. Moreover, tumor cells overexpressing HO-1 are hardly treatable with common anticancer therapies, such as radiotherapy, chemotherapy, and photodynamic therapy. Finally, HO-1 induction is also related to the acquisition of resistance to common anticancer drugs, such as the antimetabolite fluorouracil [22,23,24]. From this general perspective, HO-1 inhibition could account for a valid strategy to be pursued in addition to the wide range of the current anticancer therapies [25]. Over the past years, we gained expertise on the design of potent HO inhibitors [15,26,27,28,29,30,31,32,33,34], we published a database (HemeOxDB, accessed 10 September 2021, http://www.researchdsf.unict.it/hemeoxdb) containing all published HO inhibitors [35] and developed a descriptive 3D-QSAR model for HO-1 inhibitors [36,37]. Motivated by the successful achievements of our in silico approaches, we again decided to exploit these computational techniques to identify novel lead compounds for HO-1 inhibition.

It is common and widely used to gain further interactions with the targeted receptor growing a compound to optimize a new chemical entity. In fact, it has been proved that during the optimization process of a hit to lead compound, the original molecule gains an average molecular weight of 85 Da [38]. This growth strategy is particularly useful in the fragment-based drug design; moreover, with recent molecular modeling technologies, it is also possible to achieve ligand growing exploration from virtual growing experiments [39], and our research group has already successfully applied in silico techniques to develop several HO-1/2 inhibitors [28,29,30,32,36,37,40]. This work describes the development of new scaffolds as HO-1 inhibitors exploiting virtual structure-guided ligand-joining experiments. The molecules were generated and classified in silico and then synthesized and tested for their activity in HO-1.

## 2. Results and Discussion

### 2.1. Molecular Modeling

**Figure 1:**
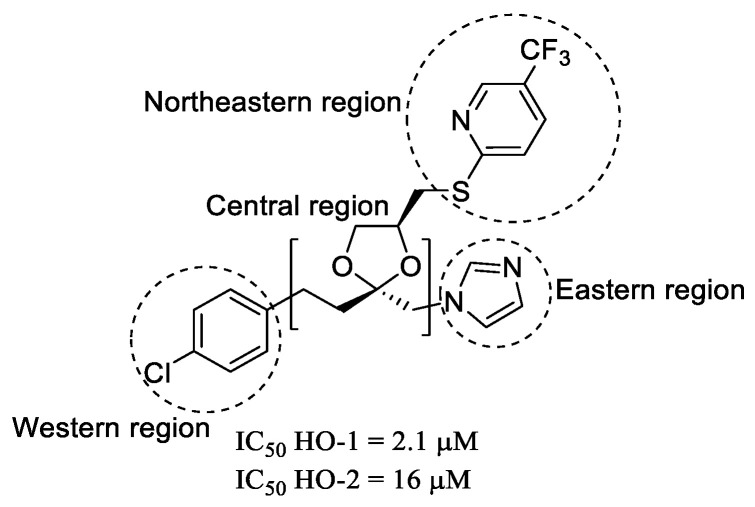
Chemical structure and IC_50_ values of representative HO-1 and HO-2 inhibitors (QC-80).

Fragment-based and virtual fragment-based drug designs are now established as productive and cost-effective starting point/optimization methods in structure-based drug discovery. In these experiments, the candidate fragments are identified and then merged or linked together to increase binding and selectivity. We recently reported a successful virtual fragment-based drug design used to develop HO-1 inhibitors where a growing approach was exploited in the growing experiments [41]. Enthusiastic about the results, in this paper, a different virtual fragment-based approach is reported. Differently from the aforementioned growing experiments, the design was achieved using a ligand joining protocol, where two different ligands inside the HO-1 binding pocket were virtually joined to connect the two fragments. The drug design experiments started by selecting common starting moieties in the typical HO-1 inhibitors’ structure. HO-1 inhibitors can be divided into four different regions, as shown in Figure 1. The northeastern region is not a requisite for HO-1 inhibition and can only increase the activity but not the HO-1/HO-2 selectivity. The eastern region, where usually an imidazole or imidazole-like moiety coordinates the iron (II) of the heme substrate in HO-1, denotes the most critical region for inhibiting heme oxygenase enzymes. In fact, thanks to this interaction, the iron (II) is protected from oxidation by disrupting an ordered solvent structure involving the critical Asp140 hydrogen-bond network (Tyr58, Tyr114, Arg136, and Asn210) and consequent displacement of water residues needed for catalysis. A central region is a connecting unit. Different types and lengths are well-tolerated, but four or five atom chains are preferred. Finally, the western region is the foremost area in controlling HO-1 potency and selectivity toward HO-2. It is crucial for the interaction with the distal hydrophobic pocket of the enzyme [15,33].

**Figure 2:**
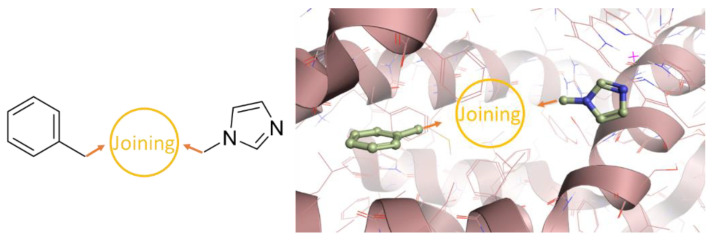
Representation of the ligand-joining experiments inside HO-1.

Taking the typical structure of the HO-1 inhibitors as a starting point, our ligand-joining experiments started from a simple imidazole and a benzyl group located in the western region, as already proved by us to be part of the western portion of some of the most powerful compounds against HO-1 (Figure 2) [33].

**Figure 3:**
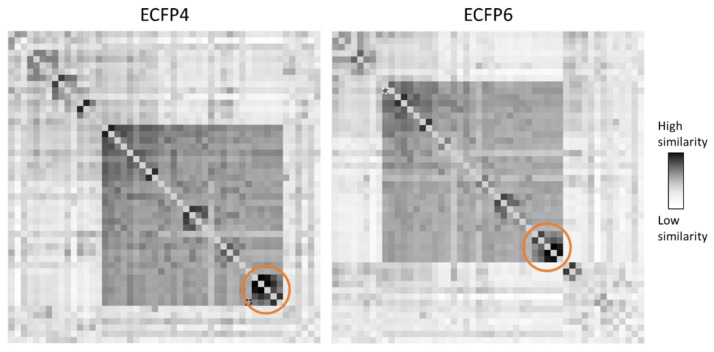
ECFP4 and FCFP4 fingerprint similarity matrix. Circled in orange is the highest similarity among the compounds (molecules **1**, **4d**, and **8a**–**c**).

In the fragment-based joining experiments, the benzyl group located in the far west area was joined to the imidazole inside the binding pocket of HO-1. One thousand compounds were generated and selected for further analysis (Table S1). Then, the potency of the compounds was evaluated and ranked by docking calculation and aligned in our previously published 3D-QSAR model [37]. Then, as already proven to be effective [42], the mean value between the two calculated potencies (*K*_i_ from docking calculation and IC_50_ from the 3D-QSAR evaluation) was used to rank all the generated molecules. To find common scaffolds to facilitate the synthesis of the final compounds, we examined the results of the best-ranked 50 compounds. To assess the molecular diversity and find out common structural clusters within the compounds, the pairwise similarity was calculated by using ECFP4/FCFP4 circular fingerprints. ECFP4/FCFP4 circular fingerprints showed that the new set of compounds covers a wide range of molecular features; interestingly, two common clusters of compounds containing the indole within the connecting unit and the western region were identified by both fingerprints (Figure 3, Tables S2 and S3). In the two different sets of compounds, the indole is substituted with a starting benzyl group either at its nitrogen atom or the 5-ring position, whereas a common connecting linker is always located in position 2. These newly identified indole-based ligands indicated that the computer-aided joining experiment gave structures with the appropriate chemical features for HO-1 inhibition with predicted potencies that reach the low micromolar range (as the average of the predicted *K*_i_ and IC_50_). To verify our procedure’s predictive capabilities, five molecules (**1**, **4d**, and **8a**–**c**) were selected, synthesized, and tested for their actual inhibition of HO-1. Moreover, three other molecules (**4a**–**c**) were also synthesized to evaluate the presence of smaller substituents in the newly identified indole central ring.

### 2.2. Chemistry

**Scheme 1:**
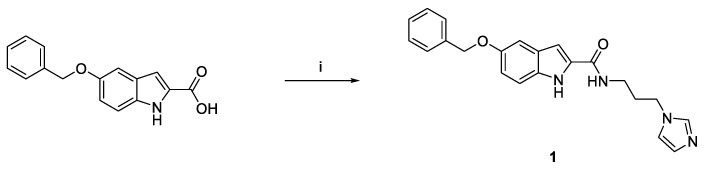
Synthesis of compound **1**. Reagents and conditions: (i) CDI, anhydrous THF, 2 h, room temperature; then, 3-(1*H*-imidazol-1-yl)propan-1-amine, 22 h, room temperature; reflux, 90 min.

The synthesis of final compound **1** is shown in Scheme 1. Compound **1** was synthesized in a one-step synthetic procedure by reaction of 5-(benzyloxy)-1*H*-indole-2-carboxylic acid, 3-(1*H*-imidazol-1-yl)propan-1-amine, and 1,1′-carbonyldiimidazole (CDI), as carboxylic acid activator, in anhydrous THF and at room temperature for 24 h and then at refluxing conditions for 90 min.

**Scheme 2:**
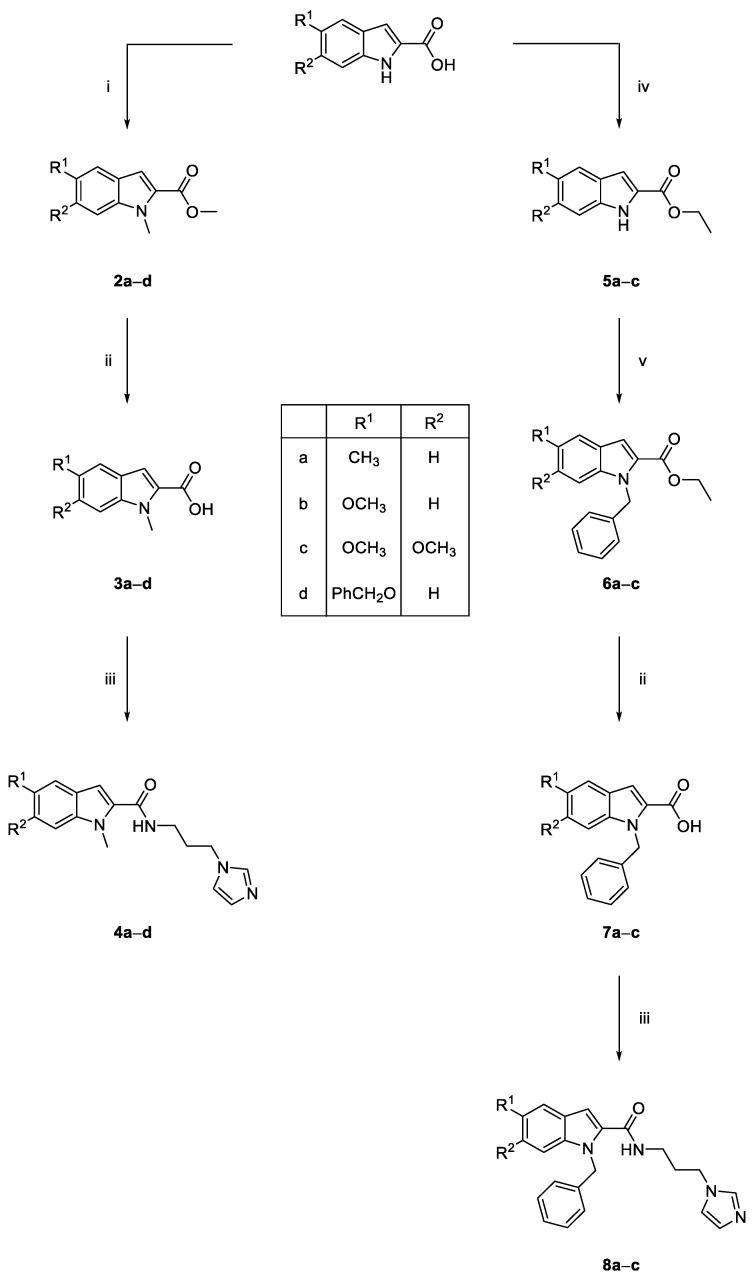
Synthetic procedure adopted for the synthesis of compounds **4a**–**d** and **8a**–**c**. Reagents and conditions: (i) CH_3_I, K_2_CO_3_, anhydrous DMF, room temperature, 48 h; (ii) LiOH, THF/H_2_O/CH_3_OH, MW, 150 psi, 150 W, 100 °C, 90 min; (iii) EDC hydrochloride, HOBt, 3-(1*H*-imidazol-1-yl)propan-1-amine, anhydrous DMF, 0 °C, then room temperature, 24 h, under Ar; (iv) EtOH, *p*-TsOH, reflux, 16 h; (v) NaH oil dispersion 80%, 0 °C, 1 h, then benzyl bromide, room temperature, 3 h, under N_2_.

Scheme 2 reports the synthesis of final compounds **4a**–**d** and **8a**–**c**. Compounds **4a**–**d** were synthesized in three steps. The first step involved the contemporary esterification of the carboxylic acid function and methylation of the indole nitrogen using a large excess of CH_3_I and K_2_CO_3_ in dry DMF at room temperature for 48 h (intermediates **2a**–**d**). Hydrolysis of intermediates **2a**–**d** was performed using LiOH monohydrate in a THF/H_2_O/CH_3_OH mixture under microwave conditions for 90 min, affording the substituted methyl-1*H*-indole-2-carboxylic acid **3a**–**d**. The last step for the synthesis of compounds **4a**–**d** involved the condensation at 0 °C of intermediates **3a**–**d** with 3-(1*H*-imidazol-1-yl)propan-1-amine using 1-ethyl-3-(3-dimethylaminopropyl)carbodiimide hydrochloride (EDC·HCl) and hydroxybenzotriazole (HOBt) as coupling agents and anhydrous DMF as the reaction solvent.

Compounds **8a**–**c** were obtained in four steps. In order to protect the carboxylic function in the subsequent synthetic steps, a Fischer esterification of the appropriate substituted 1*H*-indole-2-carboxylic acid with refluxing ethanol and *p*-toluenesulfonic acid for 16 h brought intermediates **5a**–**c**. In the second step, the benzylation of compounds **5a**–**c** was performed using benzyl bromide and NaH (oil dispersion 80%) as the base and anhydrous DMF as the reaction solvent for 3 h at room temperature and under a N_2_ atmosphere, obtaining intermediates **6a**–**c**. The final two steps involved the basic hydrolysis of ethyl esters **6a**–**c** (compounds **7a**–**c**) and the condensation of carboxylic acids **7a**–**c** with 3-(1*H*-imidazol-1-yl)propan-1-amine following the same synthetic procedure described above for the synthesis of compounds **3a**–**d** and **4a**–**d**, respectively.

### 2.3. In Vitro HO-1 Inhibition and Structure–Activity Relationships Studies

The inhibitory potency of the novel compounds was evaluated reproducing in vitro the HO-1 catalytic cycle. Rat spleen microsomal fractions were chosen to isolate HO-1 in its most native form; indeed, it is well known that in basal conditions, HO-1 is highly expressed in spleen tissues. In the presence of a strong HO-1 inhibitor, the amount of heme catabolites will be drastically reduced. Among the three catabolites, bilirubin concentrations can easily be monitored spectrophotometrically; the lowest is the absorbance detected with the assay, and the highest is the potency of the putative inhibitor. Results obtained from the in vitro enzymatic assay are reported in Table 1, using Azalanstat as the reference compound.

Looking at the poses inside the HO-1 from the docking calculation (Figures S17–S19), is it possible to classify the poses of the compounds in three different clusters accordingly with their structures. Molecules **1** and **4d** are part of the first, molecules **4a**–**c** are part of the second, and molecules **8a**–**c** are part of the third. The *N*-unsubstituted compound **1** showed a low IC_50_ value of 89.6 μM. The insertion of a substituent to the nitrogen atom of the indole ring led to compounds **4a**–**d** and **8a**–**c**. As a general trend, the *N*-methyl-1*H*-indole-2-carboxamides **4a**–**d** displayed better IC_50_ values when compared to their *N*-benzyl derivatives **8a**–**c**. All the molecules of the second cluster can be correctly allocated inside the binding pocket. However, any of them is able to reach strong stabilizing interaction, resulting in binders weaker than molecule **4d**. In the third cluster, the presence of the benzyl group in the nitrogen of the central core produces a series of bulkier compounds where the benzyl is not reaching any relevant interaction inside the pocket. From the obtained results, it is possible to speculate that the nature of the substituent on the indole ring plays an important role in the inhibitory potency of the novel compounds. Indeed, a small electron-donating substituent as the methyl group in the 5 position brought the worst results for both the *N*-methyl and the *N*-benzyl substituted compounds (367.31 μM for compound **4a** and 2063 μM for compound **8a**). The introduction of a methoxy group in the 5 position on the indole ring slightly ameliorated the IC_50_ values when compared to the 5-methyl derivatives from three- to six-fold (compare **4b** vs. **4a** and **8b** vs. **8a**, respectively), even if the IC_50_ values were higher than 100 μM. An additional methoxy group in position 6 on the indole ring afforded compounds **4c** and **8c**, which were two- and four-fold more potent than **4b** and **4c**, respectively. Methylation of the nitrogen atom of the indole ring of compound **1** led to the most potent compound of the series (compound **4d**), which displayed an IC_50_ value of 1.03 μM and was 87-fold more potent than its parent compound **1**. Molecules of the first docking cluster allocate their benzyl substituent in the far western pocket, whereas the indole at the central core could be flipped in two different ways that apparently are responsible for the difference in potency of the two compounds. When no substituent is present on the nitrogen of the indole, the hydrogen is pointing out the binding pocket; differently, when a methyl group is present, this is allocated in a different way inside the pocket, increasing the hydrophobic interaction and stabilizing the molecule, resulting in a better inhibitor activity of molecule **4d**.

## 3. Materials and Methods

### 3.1. Chemistry

Materials and chemicals were purchased from Fisher Scientific (Waltham, MA, USA), Merck (Kenilworth, NJ, USA), CEM Corporation (Charlotte, NC, USA), as reagent grade or better, and were used with no further manipulation. Solvents and NMR solvents were purchased from Fisher Scientific, Merck, and VWR (Radnor, PA, USA). Microwave-assisted reactions were performed using a CEM Discover instrument in closed Pyrex glass tubes (ca. 10 mL) with Teflon-coated septa. Reactions were monitored on TLC aluminum sheets coated with silica gel (60 F254, Merck, Kenilworth, NJ, USA) and visualized by UV (λ = 254 and 366 nm) and iodine vapors. Compounds were purified by flash chromatography employing Merck silica gel (60, 0.040–0.063 mm, 230–400 mesh) as stationary phase and using glass columns. Infrared spectra were recorded on a Perkin Elmer 281 FTIR spectrometer using KBr disks or NaCl plates. ^1^H- and ^13^C-NMR spectra were determined with Varian Unity Inova 200 and 500 MHz instruments in CDCl_3_ or DMSO-*d*_6_ solution. Chemical shifts are given in ppm values, using tetramethylsilane (TMS) as the internal standard; coupling constants (*J*) are given in Hz. Signal multiplicities are characterized as s (singlet), d (doublet), t (triplet), q (quartet), p (pentet), m (multiplet), and br (broad). Melting points were determined in an IA9200 Electrothermal apparatus equipped with a digital thermometer in capillary glass tubes and are uncorrected. Elemental analyses (C, H, N) were executed on a Carlo Erba Elemental Analyzer Mod. 1108; the purity of compounds was ≥95%, and results were within ±0.4% of the theoretical values.

#### 3.1.1. Synthesis of N-(3-(1H-Imidazol-1-yl)propyl)-5-(benzyloxy)-1H-indole-2-carboxamide (1)

In a round-bottom flask, 5-(benzyloxy)-1*H*-indole-2-carboxylic acid (0.374 mmol, 1 eq.) and 1,1′-carbonyldiimidazole (0.374 mmol, 1 eq.) were dissolved in anhydrous THF (2 mL) and stirred at room temperature for 2 h. Then, 3-(1*H*-imidazol-1-yl)propan-1-amine (0.374 mmol, 1 eq.) was added, and the obtained suspension was stirred at room temperature for 22 h and refluxed for 90 min. After this time, 200 µL of deionized H_2_O were added to the suspension, after which the solution turned bright yellow. The mixture was refluxed for 1 h and then cooled to room temperature. The reaction solvent was removed under reduced pressure and to the residue were added CH_2_Cl_2_ and NaOH 1M. The two phases were separated; the organic phase was washed three times with deionized H_2_O and then dried with anhydrous Na_2_SO_4_, filtered, and concentrated. The obtained yellow solid was triturated with diethylether and filtered under vacuum, giving the pure desired product as a pale yellow solid (12%): mp 204–206 °C; IR (KBr) cm^−1^ 3446 (br), 3281, 3104, 2946, 1622, 1557, 1508, 1445, 1430, 1388, 1330, 1231, 1180, 1116, 1079, 1013, 916, 846, 820, 745, 696, 660; ^1^H NMR (500 MHz, DMSO-*d*_6_): δ 11.34 (s, 1H, NH), 8.48 (t, *J* = 5,5 Hz, 1H, CONH), 7.74 (s, 1H, imidazole), 7.44 (d, *J* = 7.5 Hz, 2H, aromatic), 7.39–7.29 (m, 4H, aromatic), 7.23 (s, 1H, imidazole), 7.15 (s, 1H, aromatic), 6.99 (s, 1H, aromatic), 6.93–6.90 (m, 2H, aromatic + imidazole), 5.07 (s, 2H, PhCH_2_O), 4.03 (t, *J* = 7.0 Hz, 2H, CH_2_-imidazole), 3.24 (dt, *J* = 5.4, 6.5 Hz, 2H, CH_2_NH), 1.96 (p, *J* = 7.0 Hz, 2H, CH_2_CH_2_CH_2_); ^13^C NMR (125 MHz, DMSO-*d*_6_): δ 161.67, 153.01, 137.83, 132.27, 132.12, 128.72, 128.10, 128.02, 127.94, 127.65, 119.93, 115.36, 113.51, 103.99, 102.64, 69.99, 44.27, 36.38, 31.04. Anal. calcd for: C_22_H_22_N_4_O_2_: C, 70.57; H, 5.92; N, 14.96. Found: C, 70.48; H, 5.91; N, 14.99.

#### 3.1.2. General Procedure for the Synthesis of Substituted 1-Methyl-1*H*-Indole Methyl Esters (**2a**–**d**)

In a round-bottom flask, the proper commercial indole (1 mmol, 1 eq.) and K_2_CO_3_ (6 mmol, 6 eq.) were suspended in 5 mL of anhydrous DMF. CH_3_I (6 mmol, 6 eq.) was dropped to the suspension, and the mixture was stirred at room temperature for 48 h. The mixture was diluted with 50 mL of deionized H_2_O and extracted with EtOAc (3 × 25 mL). The organic phase was dried with anhydrous Na_2_SO_4_, filtered, and concentrated under reduced pressure. The residue was purified by flash chromatography eluting with a gradient mixture of cyclohexane/EtOAc. According to this procedure, the following products were obtained.

##### Methyl 1,5-Dimethyl-1*H*-indole-2-carboxylate (**2a**)

White solid (92%): mp 72.5–73.5 °C; IR (KBr) cm^−1^ 1715, 1523, 1471, 1450, 1399, 1224, 1137, 1089, 798, 764, 740; ^1^H NMR (500 MHz, DMSO-*d*_6_): δ 7.48–7.45 (m, 2H, aromatic), 7.19–7.16 (m, 2H, aromatic), 3.99 (s, 3H, NCH_3_), 3.84 (s, 3H, COOCH_3_), 2.38 (s, 3H, CH_3_). Anal. calcd for: C_12_H_13_NO_2_: C, 70.92; H, 6.45; N, 6.89. Found: C, 70.86; H, 6.43; N, 6.90.

##### Methyl 5-Methoxy-1-methyl-1*H*-indole-2-carboxylate (**2b**)

White solid (77%): mp 123–125 °C; IR (KBr) cm^−1^ 3399 (br), 2955, 1706, 1519, 1474, 1430, 1220, 1141, 1089, 1025, 843, 799, 762, 736; ^1^H NMR (500 MHz, DMSO-*d*_6_): δ 7.50 (d, *J* = 9.0 Hz, 1H, aromatic), 7.17–7.13 (m, 2H, aromatic), 7.00 (dd, *J* = 9.0, 2.5 Hz, 1H, aromatic), 3.99 (s, 3H, NCH_3_), 3.84 (s, 3H, OCH_3_), 3.77 (s, 3H, COOCH_3_). Anal. calcd for: C_12_H_13_NO_3_: C, 65.74; H, 5.98; N, 6.39. Found: C, 65.87; H, 5.99; N, 6.37.

##### Methyl 5,6-Dimethoxy-1-methyl-1*H*-indole-2-carboxylate (**2c**)

Pale yellow solid (95%): mp 118–120 °C; IR (KBr) cm^−1^ 2996, 2958, 1703, 1628, 1516, 1474, 1448, 1350, 1223, 1141, 1089, 1007, 846, 799, 760; ^1^H NMR (500 MHz, DMSO-*d*_6_): δ 7.13 (s, 1H, aromatic), 7.11 (s, 1H, aromatic), 7.08 (s, 1H, aromatic), 3.99 (s, 3H, NCH_3_), 3.86 (s, 3H, 5-OCH_3_), 3.81 (s, 3H, 6-OCH_3_), 3.77 (s, 3H, COOCH_3_). Anal. calcd for: C_13_H_15_NO_4_: C, 62.64; H, 6.07; N, 5.62. Found: C, 62.73; H, 6.06; N, 5.63.

##### Methyl 5-(Benzyloxy)-1-methyl-1*H*-indole-2-carboxylate (**2d**)

White solid (89%): mp 134–135 °C; IR (KBr) cm^−1^ 3397 (br), 2956, 2907, 1716, 1516, 1466, 1436, 1386, 1296, 1256, 1226, 1200, 1148, 1090, 1012, 852, 803; ^1^H NMR (200 MHz, DMSO-*d*_6_): δ 7.55–7.32 (m, 6H, aromatic), 7.24 (d, *J* = 2.2 Hz, 1H, aromatic), 7.16 (s, 1H, aromatic), 7.08 (dd, *J* = 9.0, 2.4 Hz, 1H, aromatic), 5.11 (s, 2H, OCH_2_), 4.00 (s, 3H, NCH_3_), 3.84 (s, 3H, COOCH_3_). Anal. calcd for: C_18_H_17_NO_3_: C, 73.20; H, 5.80; N, 4.74. Found: C, 73.07; H, 5.81; N, 4.73.

#### 3.1.3. General Procedure for the Synthesis of *N*-Substituted 1*H*-Indole-2-carboxylic Acids (**3a**–**d**, **7a**–**c**)

In a sealed Pyrex tube equipped with a magnetic stirrer, the proper carboxylic acid (**2a**–**d**, **6a**–**c**) (1 eq.) and LiOH monohydrate (2 eq.) were suspended in a mixture of THF/H_2_O/CH_3_OH (3:1.5:1 ratio). The reaction was stirred under microwave irradiation (150 psi, 150 W, 100 °C) for 90 min. The reaction solvents were removed under reduced pressure, and to the residue was added HCl 1M. The suspension was stirred at room temperature for 30 min. The solid was filtered under reduced pressure and repeatedly washed with deionized H_2_O, affording the pure products that needed no further purification. According to this procedure, the following products were obtained.

##### 1,5-Dimethyl-1*H*-indole-2-carboxylic Acid (**3a**)

The title compound was obtained using 0.81 mmol of compound **2a** and 1.62 mmol of LiOH monohydrate in a mixture of THF (1.8 mL), H_2_O (904 μL), and CH_3_OH (602 μL). White solid (85%): mp 219 °C; IR (KBr) cm^−1^ 2918, 1679, 1528, 1474, 1414, 1273, 1235, 1134, 910, 882, 785; ^1^H NMR (500 MHz, DMSO-*d*_6_): δ 7.45–7.42 (m, 2H, aromatic), 7.16–7.14 (m, 1H, aromatic), 7.09 (br s, 1H, aromatic), 3.99 (s, 3H, NCH_3_), 2.38 (s, 3H, CH_3_). Anal. calcd for: C_11_H_11_NO_2_: C, 69.83; H, 5.86; N, 7.40. Found: C, 69.98; H, 5.85; N, 7.41.

##### 5-Methoxy-1-methyl-1*H*-indole-2-carboxylic Acid (**3b**)

The title compound was obtained using 0.72 mmol of compound **2b** and 1.44 mmol of LiOH monohydrate in a mixture of THF (1.6 mL), H_2_O (818 μL), and CH_3_OH (602 μL). White solid (88%): mp 217 °C; IR (KBr) cm^−1^ 3448, 2959, 1687, 1519, 1472, 1266, 1231, 1217, 1142, 1027, 846, 800; ^1^H NMR (500 MHz, DMSO-*d*_6_): δ 7.47 (d, *J* = 9.0 Hz, 1H, aromatic), 7.14–7.12 (m, 2H, aromatic), 6.98 (dd, *J* = 9.0, 2.0 Hz, 1H, aromatic), 3.99 (s, 3H, NCH_3_), 3.77 (s, 3H, OCH_3_). Anal. calcd for: C_11_H_11_NO_3_: C, 64.38; H, 5.40; N, 6.83. Found: C, 64.44; H, 5.39; N, 6.85.

##### 5,6-Dimethoxy-1-methyl-1*H*-indole-2-carboxylic Acid (**3c**)

The title compound was obtained using 0.80 mmol of compound **2c** and 1.6 mmol of LiOH monohydrate in a mixture of THF (1.6 mL), H_2_O (904 μL), and CH_3_OH (545 μL). White solid (quantitative): mp 210 °C; IR (KBr) cm^−1^ 3538, 3489, 2947, 1683, 1636, 1519, 1476, 1231, 1143, 1095, 1003, 858, 803, 768; ^1^H NMR (500 MHz, DMSO-*d*_6_): δ 7.10–7.06 (m, 3H, aromatic), 3.98 (s, 3H, NCH_3_), 3.86 (s, 3H, 5-OCH_3_), 3.77 (s, 3H, 6-OCH_3_). Anal. calcd for: C_12_H_13_NO_4_: C, 61.27; H, 5.57; N, 5.95. Found: C, 61.38; H, 5.55; N, 5.94.

##### 5-(Benzyloxy)-1-methyl-1*H*-indole-2-carboxylic Acid (**3d**)

The title compound was obtained using 0.64 mmol of compound **2** and 1.28 mmol of LiOH monohydrate in a mixture of THF (1.36 mL), H_2_O (682 μL) and CH_3_OH (454 μL). White solid (96%): mp > 300 °C (dec.); IR (KBr) cm^−1^ 3500, 2952, 1700, 1524, 1478, 1428, 1380, 1296, 1207, 1132, 1083, 806; ^1^H NMR (200 MHz, DMS O-*d*_6_): δ 7.50–7.28 (m, 6H, aromatic), 7.11 (d, *J* = 2.0 Hz, 1H, aromatic), 6.87 (dd, *J* = 9.0, 2.0 Hz, 1H, aromatic), 6.71 (s, 1H, aromatic), 5.08 (s, 2H, PhCH_2_O), 4.01 (s, 3H, NCH_3_). Anal. calcd for: C_17_H_15_NO_3_: C, 72.58; H, 5.37; N, 4.98. Found: C, 72.41; H, 5.36; N, 4.99.

##### 1-Benzyl-5-methyl-1*H*-indole-2-carboxylic Acid (**7a**)

The title compound was obtained using 0.655 mmol of compound **6a** and 1.31 mmol of LiOH monohydrate in a mixture of THF (1.6 mL), H_2_O (818 μL), and CH_3_OH (545 μL). White solid (81%): mp 198–199 °C; IR (KBr) cm^−1^ 3447, 1670, 1531, 1465, 1453, 1426, 1277, 1216, 1127, 903, 791, 736; ^1^H NMR (200 MHz, DMSO-*d*_6_): δ 7.47–6.98 (m, 9H, aromatic), 5.85 (s, 2H, PhCH_2_), 2.36 (s, 3H, CH_3_). Anal. calcd for: C_17_H_15_NO_2_: C, 76.96; H, 5.70; N, 5.28. Found: C, 77.12; H, 5.69; N, 5.29.

##### 1-Benzyl-5-methoxy-1*H*-indole-2-carboxylic Acid (**7b**)

The title compound was obtained using 0.64 mmol of compound **6** and 1.28 mmol of LiOH monohydrate in a mixture of THF (1.36 mL), H_2_O (682 μL), and CH_3_OH (454 μL). White solid (80%): mp 174–175 °C; IR (KBr) cm^−1^ 3506 (br), 1664, 1519, 1458, 1421, 1266, 1219, 1172, 1032, 708; ^1^H NMR (500 MHz, CDCl3): δ 7.43 (s, 1H, aromatic), 7.27–7.20 (m, 4H, aromatic), 7.10 (d, *J* = 2.5 Hz, 1H, aromatic), 7.04 (d, *J* = 9.0 Hz, 2H, aromatic), 7.00 (dd, *J* = 9.0, 2.5 Hz, 1H, aromatic), 5.81 (s, 2H, PhCH_2_O), 3.85 (s, 3H, OCH_3_). Anal. Calcd for: C_17_H_15_NO_3_: C, 72.58; H, 5.37; N, 4.98. Found: C, 72.70; H, 5.39; N, 4.97.

##### 1-Benzyl-5,6-dimethoxy-1*H*-indole-2-carboxylic Acid (**7c**)

The title compound was obtained using 0.133 mmol of compound **6** and 0.266 mmol of LiOH monohydrate in a mixture of THF (0.327 μL), H_2_O (167 μL), and CH_3_OH (111 μL). White solid (79%): mp 188 °C; IR (KBr) cm^−1^ 3457, 1652, 1520, 1493, 1455, 1250, 1216, 1158, 1011; ^1^H NMR (200 MHz, CDCl_3_): δ 7.42 (s, 1H, aromatic), 7.28–7.24 (m, 3H, aromatic), 7.07–7.03 (m, 3H, aromatic), 6.69 (s, 1H, aromatic), 5.81 (s, 2H, PhCH_2_O), 3.93 (s, 3H, 5-OCH_3_), 3.86 (s, 3H, 6-OCH_3_). Anal. calcd for: C_18_H_17_NO_4_: C, 69.44; H, 5.50; N, 4.50. Found: C, 69.32; H, 5.51; N, 4.51.

#### 3.1.4. General Procedure for the Synthesis of *N*-Substituted 1*H*-Indole-2-carboxamides (**4a**–**d**, **8a**–**c**)

In a flame-dried two-necked round-bottom flask, the appropriate carboxylic acid (**3a**–**d**, **7a**–**c**, 1 eq.) was dissolved in anhydrous DMF in an argon atmosphere. The obtained solution was cooled to 0 °C with an ice bath. EDC hydrochloride (1.5 eq.) was added, and the reaction was stirred for 15 min. After this time, HOBt (1.5 eq.) was added, and the mixture was stirred for a further 15 min. Finally, 3-(1*H*-imidazol-1-yl)propan-1-amine (2 eq.) was added to the solution, and the reaction was slowly warmed at room temperature and left to react for 24 h under an argon atmosphere. The mixture was concentrated, diluted with an aqueous solution of NaHCO_3_ 5%, and extracted three times with EtOAc. The organic phases were dried with anhydrous Na_2_SO_4_, filtered, and concentrated. The crude was purified by flash chromatography eluting with a gradient mixture of CH_2_Cl_2_/CH_3_OH. According to this procedure, the following products were obtained.

##### *N*-(3-(1*H*-Imidazol-1-yl)propyl)-1,5-dimethyl-1*H*-indole-2-carboxamide (**4a**)

The title compound was obtained using 0.57 mmol of compound **3a**, 0.78 mmol of EDC hydrochloride, 0.78 mmol of HOBt, and 1.04 mmol of 3-(1*H*-imidazol-1-yl)propan-1-amine in 5 mL of anhydrous DMF. Whitish solid (75%): mp 102–103 °C; IR (KBr) cm^−1^ 3447 (br), 3218, 2934, 1645, 1552, 1515, 1466, 1394, 1305, 1270, 1230, 1086; 812, 745; ^1^H NMR (500 MHz, DMSO-*d*_6_): δ 8.50 (t, *J* = 5.6 Hz, 1H, CONH), 7.67 (s, 1H, imidazole), 7.44–7.35 (m, 2H, aromatic), 7.21 (s, 1H, aromatic), 7.09 (dd, *J* = 8.6, 1H, aromatic), 6.96 (s, 1H, imidazole), 6.89 (s, 1H, imidazole), 4.03 (t, *J* = 6.9 Hz, 2H, CH_2_-imidazole), 3.92 (s, 3H, NCH_3_), 3.22 (dt, *J* = 5.7, 6.5 Hz, 2H, CONHCH_2_), 2.37 (s, 3H, CH_3_), 1.96 (p, *J* = 6.9 Hz, 2H, CH_2_CH_2_CH_2_); ^13^C NMR (125 MHz, DMSO-*d*_6_): δ 162.34, 137.52, 137.11, 132.28, 128.99, 128.44, 125.97, 125.52, 120.95, 119.63, 110.35, 103.88, 44.01, 36.26, 31.50, 30.89, 21.21. Anal. calcd for: C_17_H_20_N_4_O: C, 68.90; H, 6.80; N, 18.90. Found: C, 68.73; H, 6.81; N, 18.86.

##### *N*-(3-(1*H*-Imidazol-1-yl)propyl)-5-methoxy-1-methyl-1*H*-indole-2-carboxamide (**4b**)

The title compound was obtained using 0.652 mmol of compound **3b**, 0.978 mmol of EDC hydrochloride, 0.978 mmol of HOBt, and 1.304 mmol of 3-(1*H*-imidazol-1-yl)propan-1-amine in 5 mL of anhydrous DMF. Pale yellow solid (54%): mp 121–122 °C; IR (KBr) cm^−1^ 3286, 2938, 1633, 1545, 1509, 1470, 1457, 1397, 1274, 1219, 1024, 840, 804; ^1^H NMR (500 MHz, DMSO-*d*_6_): δ 8.49 (t, *J* = 5.5 Hz, 1H, CONH), 7.67 (s, 1H, imidazole), 7.42 (d, *J* = 9.0 Hz, 1H, aromatic), 7.22 (s, 1H, aromatic), 7.10 (d, *J* = 2.0 Hz, 1H, aromatic), 6.98 (s, 1H, imidazole), 6.93–6.90 (m, 2H, aromatic + imidazole), 4.04 (t, *J* = 7.0 Hz, 2H, CH_2_-imidazole), 3.94 (s, 3H, NCH_3_), 3.77 (s, 3H, OCH_3_), 3.23 (dt, *J* = 5.6, 6.5 Hz, 2H, CONHCH_2_), 1.97 (p, *J* = 7.0 Hz, 2H, CH_2_CH_2_CH_2_); ^13^C NMR (125 MHz, DMSO-*d*_6_): δ 162.20, 154.16, 137.47, 133.94, 132.52, 128.40, 126.00, 119.57, 114.48, 111.47, 103.93, 102.32, 55.49, 43.96, 36.22, 31.54, 30.86. Anal. calcd for: C_17_H_20_N_4_O_2_: C, 65.37; H, 6.45; N, 17.94. Found: C, 65.29; H, 6.46; N, 17.99.

##### *N*-(3-(1*H*-Imidazol-1-yl)propyl)-5,6-dimethoxy-1-methyl-1*H*-indole-2-carboxamide (**4c**)

The title compound was obtained using 0.703 mmol of compound **3c**, 1.055 mmol of EDC hydrochloride, 1.055 mmol of HOBt, and 1.406 mmol of 3-(1*H*-imidazol-1-yl)propan-1-amine in 5 mL of anhydrous DMF. Pale yellow solid (71%): mp 146–147 °C; IR (KBr) cm^−1^ 3228, 2942, 1649, 1624, 1540, 1508, 1473, 1395, 1246, 1215, 1094, 1001, 840, 813; ^1^H NMR (500 MHz, DMSO-*d*_6_): δ 8.35 (t, *J* = 5.5 Hz, 1H, CONH), 7.68 (s, 1H, imidazole), 7.22 (s, 1H, imidazole), 7.09 (s, 1H, aromatic), 7.04 (s, 1H, aromatic), 6.97 (s, 1H, aromatic), 6.91 (s, 1H, imidazole), 4.03 (t, *J* = 7.0 Hz, 2H, CH_2_-imidazole), 3.95 (s, 3H, NCH_3_), 3.84 (s, 3H, 5-OCH_3_), 3.77 (s, 3H, 6-OCH_3_), 3.22 (dt, *J* = 5.6, 6.5 Hz, 2H, CONHCH_2_), 1.96 (p, *J* = 7.0 Hz, 2H, CH_2_CH_2_CH_2_); ^13^C NMR (125 MHz, DMSO-*d*_6_): δ 162.35, 148.70, 145.62, 137.53, 133.78, 130.38, 128.41, 119.66, 118.30, 104.58, 102.82, 93.54, 55.98, 55.90, 44.05, 36.20, 31.73, 30.99. Anal. calcd for: C_18_H_22_N_4_O_3_: C, 63.14; H, 6.48; N, 16.36. Found: C, 63.23; H, 6.49; N, 16.31.

##### *N*-(3-(1*H*-Imidazol-1-yl)propyl)-5-(benzyloxy)-1-methyl-1*H*-indole-2-carboxamide (**4d**)

The title compound was obtained using 0.355 mmol of compound **3d**, 0.533 mmol of EDC hydrochloride, 0.533 mmol of HOBt, and 0.71 mmol of 3-(1*H*-imidazol-1-yl)propan-1-amine in 5 mL of anhydrous DMF. White solid (87%): mp 174–175 °C; IR (KBr) cm^−1^ 3194, 1635, 1551, 1506, 1452, 1388, 1269, 1235, 1200, 1161, 1101, 1072, 1013, 845, 751, 707; ^1^H NMR (500 MHz, DMSO-*d*_6_): δ 8.48 (t, *J* = 5.7 Hz, 1H, CONH), 7.66 (s, 1H, imidazole), 7.48–7.36 (m, 5H, aromatic), 7.34–7.29 (m, 1H, aromatic), 7.22–7.20 (m, 1H, aromatic), 7.19 (d, *J* = 2.4 Hz, 1H, imidazole), 7.01–6.98 (m, 1H, aromatic), 6.97–6.96 (m, 1H, aromatic), 6.90–6.89 (m, 1H, imidazole), 5.11 (s, 2H, PhCH_2_O), 4.03 (t, *J* = 6.9 Hz, 2H, CH_2_-imidazole), 3.93 (s, 3H, NCH_3_), 3.22 (dt, *J* = 5.7, 6.5 Hz, 2H, CONHCH_2_), 1.96 (p, *J* = 6.9 Hz, 2H, CH_2_CH_2_CH_2_); ^13^C NMR (125 MHz, DMSO-*d*_6_): δ 162.03, 153.02, 137.57, 133.97, 132.54, 128.42, 128.39, 127.72, 127.70, 125.85, 119.43, 114.92, 111.41, 103.91, 103.85, 69.77, 43.83, 36.12, 31.45, 30.80. Anal. calcd for: C_23_H_24_N_4_O_2_: C, 71.11; H, 6.23; N, 14.42. Found: C, 70.98; H, 6.22; N, 14.45.

##### *N*-(3-(1*H*-Imidazol-1-yl)propyl)-1-benzyl-5-methyl-1*H*-indole-2-carboxamide (**8a**)

The title compound was obtained using 0.291 mmol of compound **7a**, 0.437 mmol of EDC hydrochloride, 0.437 mmol of HOBt, and 0.582 mmol of 3-(1*H*-imidazol-1-yl)propan-1-amine in 2.5 mL of anhydrous DMF. White solid (72%): mp 178–179 °C; IR (KBr) cm^−1^ 3448 (br), 3198, 2923, 1654, 1560, 1506, 1456, 1352, 1302, 1278, 1223, 1082, 917, 789; ^1^H NMR (500 MHz, DMSO-*d*_6_): δ 8.57 (t, *J* = 5.7 Hz, 1H, CONH), 7.63 (s, 1H, imidazole), 7.41 (s, 1H, aromatic), 7.36 (d, *J* = 8.5 Hz, 1H, aromatic), 7.24–7.13 (m, 4H), 7.04 (s, 1H, aromatic), 7.03–6.99 (m, 3H, aromatic + imidazole), 6.89 (s, 1H, imidazole), 5.79 (s, 2H, PhCH_2_), 3.94 (t, *J* = 6.9 Hz, 2H, CH_2_-imidazole), 3.19 (dt, *J* = 5.8, 6.5 Hz, 2H, CONHCH_2_), 2.35 (s, 3H, CH_3_), 1.91 (p, 2H, *J* = 6.9 Hz CH_2_CH_2_CH_2_); ^13^C NMR (125 MHz, DMSO-*d*_6_): δ 162.42, 139.10, 136.71, 132.04, 129.40, 128.54, 128.35, 127.16, 126.66, 126.30, 125.79, 121.18, 119.67, 110.92, 104.87, 46.99, 43.95, 36.23, 30.87, 21.20. Anal. calcd for: C_23_H_24_N_4_O: C, 74.17; H, 6.49; N, 15.04. Found: C, 73.99; H, 6.47; N, 15.08.

##### *N*-(3-(1*H*-Imidazol-1-yl)propyl)-1-benzyl-5-methoxy-1*H*-indole-2-carboxamide (**8b**)

The title compound was obtained using 0.495 mmol of compound **7b**, 0.743 mmol of EDC hydrochloride, 0.743 mmol of HOBt, and 0.99 mmol of 3-(1*H*-imidazol-1-yl)propan-1-amine in 5 mL of anhydrous DMF. White solid (75%): mp 151–152 °C; IR (KBr) cm^−1^ 3245, 2928, 1643, 1543, 1450, 1349, 1271, 1230, 1078, 1030, 795; ^1^H NMR (500 MHz, DMSO-*d*_6_): δ 8.57 (t, *J* = 5.7 Hz, 1H, CONH), 7.61 (s, 1H, imidazole), 7.39 (d, *J* = 9.0 Hz, 1H, aromatic), 7.24–7.19 (m, 2H, aromatic), 7.18–7.13 (m, 2H, aromatic + imidazole), 7.12 (d, *J* = 2.4 Hz, 1H aromatic), 7.05 (s, 1H, aromatic), 7.04–7.01 (m, 2H, aromatic), 6.90–6.84 (m, 2H, aromatic + imidazole), 5.80 (s, 2H, PhCH_2_), 3.95 (t, *J* = 6.9 Hz, 2H, CH_2_-imidazole), 3.75 (s, 3H, OCH_3_), 3.20 (dt, *J* = 5.8, 6.5 Hz, 2H, CONHCH_2_), 1.92 (p, *J* = 6.8 Hz, 2H, CH_2_CH2CH_2_); ^13^C NMR (125 MHz, DMSO-*d*_6_): δ 162.16, 154.26, 139.00, 137.37, 133.38, 132.23, 128.40, 127.01, 126.54, 126.31, 119.45, 114.58, 111.94, 104.81, 102.50, 55.44, 46.93, 43.76, 36.11, 30.80. Anal. calcd for: C_23_H_24_N_4_O_2_: C, 71.11; H, 6.23; N, 14.42. Found: C, 71.00; H, 6.22; N, 14.45.

##### *N*-(3-(*1H*-Imidazol-1-yl)propyl)-1-benzyl-5,6-dimethoxy-1*H*-indole-2-carboxamide (**8c**)

The title compound was obtained using 0.104 mmol of compound **7c**, 0.156 mmol of EDC hydrochloride, 0.156 mmol of HOBt, and 0.208 mmol of 3-(1*H*-imidazol-1-yl)propan-1-amine in 1.5 mL of anhydrous DMF. White solid (46%): mp 110–111 °C; IR (KBr) cm^−1^ 3422 (br), 3309, 1628, 1542, 1443, 1266, 1224, 1163, 1111, 844, 705; ^1^H NMR (500 MHz, DMSO-*d*_6_): δ 8.41 (t, *J* = 6.0 Hz, 1H, CONH), 7.61 (s, 1H, imidazole), 7.23–7.20 (m, 2H, aromatic), 7.17–7.14 (m, 2H, aromatic), 7.10 (s, 1H, imidazole), 7.05–7.02 (m, 4H, aromatic), 6.88 (s, 1H, imidazole), 5.81 (s, 2H, PhCH_2_), 3.93 (t, *J* = 6.5 Hz, 2H, CH_2_-imidazole), 3.75 (s, 3H, 5-OCH_3_), 3.74 (s, 3H, 6-OCH_3_), 3.17 (dt, *J* = 6.1, 6.9 Hz, 2H, CONHCH_2_), 1.90 (p, *J* = 6.5 Hz, 2H, CH_2_CH_2_CH_2_); ^13^C NMR (125 MHz, DMSO-*d*_6_): δ 162.29, 148.70, 145.74, 139.18, 137.44, 133.33, 130.11, 128.44, 128.40, 127.03, 126.69, 119.55, 118.61, 105.53, 102.94, 94.04, 55.95, 55.89, 46.92, 43.83, 36.06, 30.95. Anal. calcd for: C_24_H_26_N4O_3_: C, 68.88; H, 6.26; N, 13.39. Found: C, 69.01; H, 6.27; N, 13.35.

#### 3.1.5. General Procedure for the Synthesis of Substituted 1*H*-Indole Ethyl Esters (**5a**–**c**)

The proper commercial indole (1 mmol, 1 eq) was dissolved in 20 mL of EtOH. *p*-toluensulfonic acid (5 mmol, 5 eq) was added to the mixture, and the solution was refluxed for 16 h. After cooling to room temperature, the reaction solvent was removed under reduced pressure, a saturated solution of Na_2_CO_3_ was added, and the mixture was extracted three times with CH_2_Cl_2_. The reunited organic phases were dried with Na_2_SO_4_, filtered, and the solvent was removed under vacuo. With the exception of compound **5c**, the obtained product did not require any purification. According to this procedure, the following products were obtained.

##### Ethyl 5-Methyl-1*H*-indole-2-carboxylate (**5a**)

Beige solid (53%): mp 158–159 °C; IR (KBr) cm^−1^ 3327, 2940, 1686, 1533, 1380, 1331, 1255, 1211, 1023, 800, 773, 743, 669; ^1^H NMR (500 MHz, CDCl_3_): δ 8.81 (s, 1H, NH), 7.46 (s, 1H, aromatic), 7.31 (d, *J* = 8.5 Hz, 1H, aromatic), 7.16 (d, *J* = 1.5 Hz, 1H, aromatic), 7.14 (d, *J* = 1.5 Hz, 1H, aromatic), 4.40 (q, *J* = 7.0 Hz, 2H, CH_2_CH_3_), 2.44 (s, 3H, CH_3_), 1.41 (t, *J* = 7.0 Hz, 3H, CH_2_CH_3_). Anal. calcd for: C_12_H_13_NO_2_: C, 70.92; H, 6.45; N, 6.89. Found: C, 70.79; H, 6.46; N, 6.87.

##### Ethyl 5-Methoxy-1*H*-indole-2-carboxylate (**5b**)

Light brownish solid (89%): mp 154–155 °C; IR (KBr) cm^−1^ 3328, 1683, 1528, 1457, 1340, 1251, 1219, 1158, 1027, 858, 774; ^1^H NMR (500 MHz, DMSO-*d*_6_): δ 11.72 (s, 1H, NH), 7.37–7.34 (m, 1H, aromatic), 7.10–7.05 (m, 2H, aromatic), 6.94–6.91 (m, 1H, aromatic), 4.34 (q, *J* = 7.0 Hz, 2H, CH_2_CH_3_), 3.76 (s, 3H, OCH_3_), 1.34 (t, *J* = 7.0 Hz, 3H, CH_2_CH_3_). Anal. calcd for: C_12_H_13_NO_3_: C, 65.74; H, 5.98; N, 6.39. Found: C, 65.61; H, 5.96 N, 6.41.

##### Ethyl 5,6-Dimethoxy-1*H*-indole-2-carboxylate (**5c**)

Purified by flash chromatography eluting with a mixture of cyclohexane/EtOAc (7:3). White solid (41%): mp 171−172 °C; IR (KBr) cm^−1^ 3314, 2986, 1682, 1523, 1501, 1458, 1348, 1285, 1250, 1213, 1193, 1145, 1028, 1006, 843, 770; ^1^H NMR (500 MHz, CDCl_3_): δ 8.84 (s, 1H, NH), 7.12 (d, *J* = 2.0 Hz, 1H, aromatic), 7.04 (s, 1H, aromatic), 6.85 (s, 1H, aromatic), 4.39 (q, *J* = 7.0 Hz, 2H, CH_2_CH_3_), 3.93 (s, 3H, 5-OCH_3_), 3.92 (s, 3H, 6-OCH_3_), 1.40 (t, *J* = 7.0 Hz, 3H, CH_2_CH_3_). Anal. calcd for: C_13_H_15_NO_4_: C, 62.64; H, 6.07; N, 5.62. Found: C, 62.72; H, 6.06; N, 5.60.

#### 3.1.6. General Procedure for the Synthesis of Substituted 1-Benzyl-1*H*-indole Ethyl Esters (**6a**–**c**)

In a two-necked round-bottom flask, NaH (oil dispersion 80%, 2 eq.) was added to 3 mL of anhydrous DMF under N_2_, and the suspension was cooled to 0 °C with an ice bath. The proper 1*H*-indole ethyl ester (1 eq.) was dissolved in 2 mL of anhydrous DMF and added dropwise via syringe to the stirring suspension, and the reaction was warmed to room temperature. After 1 h, benzyl bromide (2 eq.) was added dropwise, and the mixture was stirred at room temperature for 3 h. Then, the mixture was poured in crushed ice and extracted with EtOAc (3 × 35 mL). The reunited organic phases were washed with deionized H_2_O (1 × 45 mL) and brine (1 × 45 mL). After drying with Na_2_SO_4_, filtering, and removing the solvent under reduced pressure, the crude was purified by flash chromatography eluting with a gradient mixture of cyclohexane/EtOAc. According to this procedure, the following products were obtained.

##### Ethyl 1-Benzyl-5-methyl-1*H*-indole-2-carboxylate (**6a**)

The title compound was obtained using 0.59 mmol of compound **5**, 1.18 mmol of NaH, and 1.18 mmol of benzyl bromide. Pale yellow waxy solid (77%): mp 61–63 °C; IR (KBr) cm^−1^ 2926, 1706, 1523, 1454, 1250, 1200, 1149, 1094, 1029, 867, 703; ^1^H NMR (200 MHz, DMSO-*d*_6_): δ 7.49–6.97 (m, 9H, aromatic), 5.83 (s, 2H, PhCH_2_), 4.27 (q, *J* = 7.0 Hz, 2H, CH_2_CH_3_), 2.37 (s, 3H, CH_3_), 1.28 (t, *J* = 7.0 Hz, 3H, CH_2_CH_3_). Anal. calcd for: C_19_H_19_NO_2_: C, 77.79; H, 6.53; N, 4.77. Found: C, 77.92; H, 6.54; N, 4.76.

##### Ethyl 1-Benzyl-5-methoxy-1*H*-indole-2-carboxylate (**6**)

The title compound was obtained using 0.655 mmol of compound **5**, 1.31 mmol of NaH, and 1.31 mmol of benzyl bromide. Pale yellow oil (95%): IR (NaCl) cm-1 3401 (br), 2982, 2933, 1704, 1624, 1520, 1454, 1419, 1251, 1213, 1094, 1030, 844, 801, 762, 736; ^1^H NMR (500 MHz, CDCl_3_): δ 7.29 (s, 1H, aromatic), 7.25–7.17 (m, 4H, aromatic), 7.09 (d, *J* = 2.5 Hz, 1H, aromatic), 7.04–7.01 (m, 2H, aromatic), 6.97 (dd, *J* = 9.0, 2.5 Hz, 1H, aromatic), 5.81 (s, 2H, PhCH_2_), 4.31 (q, *J* = 7.0 Hz, 2H, CH_2_CH_3_), 3.84 (s, 3H, OCH_3_), 1.34 (t, *J* = 7.0 Hz, 3H, CH_2_CH_3_). Anal. calcd for: C_19_H_19_NO_3_: C, 73.77; H, 6.19; N, 4.53. Found: C, 73.58; H, 6.20; N, 4.54.

##### Ethyl 1-Benzyl-5,6-dimethoxy-1*H*-indole-2-carboxylate (**6c**)

The title compound was obtained using 0.324 mmol of compound **5**, 0.648 mmol of NaH, and 0.648 mmol of benzyl bromide. Colorless oil (41%): IR (NaCl) cm^−1^ 3534 (br), 2935, 1694, 1630, 1520, 1494, 1454, 1414, 1251, 1216, 1154, 1089,1029, 846, 737, 703; ^1^H NMR (200 MHz, CDCl_3_): δ 7.36–7.19 (m, 5H, aromatic), 7.06–7.01 (m, 3H, aromatic), 6.71 (s, 1H, aromatic), 5.80 (s, 2H, PhCH_2_), 4.29 (q, *J* = 7.0 Hz, 2H, CH_2_CH_3_), 3.92 (s, 3H,5-OCH_3_), 3.85 (s, 3H,6-OCH_3_), 1.33 (t, *J* = 7.0 Hz, 3H, CH_2_CH_3_). Anal. calcd for: C_20_H_21_NO_4_: C, 70.78; H, 6.24; N, 4.13. Found: C, 70.59; H, 6.23; N, 4.14.

### 3.2. Molecular Modeling

The molecules’ structures were built using Marvin Sketch with a protonation state calculated at neutral pH. The 2D molecules were initially subjected to a molecular mechanics energy minimization by the Merck molecular force field (MMFF94); then, they were optimized at the semiempirical level employing the parameterized model number 3 (PM3) Hamiltonian as implemented in the MOPAC2016 package (Keywords used in MOPAC2016: PM3, MMOK, XYZ, BONDS, DDMIN = 0, PRECISE, GNORM = 0.01, CHECK) [43,44]. The pairwise similarity was calculated using ECFP4/FCFP4 circular fingerprints using Forge 10.6.0 Revision: 36004 (Cresset Biomolecular Discovery Ltd., Litlington, UK). Spark (accessed on 10 September 2021, v. 10.6.0, https://www.cresset-group.com/products/spark/) was used for the ligand-growing experiments using Cresset’s molecular field points (local extrema of the electrostatic, van der Waals, and the molecule’s hydrophobic potentials) [45]. The 1000 molecules derived from the ligand growing experiments were generated using a database of 1,589,469 fragments (Figure S20). Docking experiments were performed using AutoDock 4.2.6 in YASARA (GUI, v. 20.12.24) [46] using 3HOK (X-ray Crystal Structure of Human Heme Oxygenase-1 with (2*R*, 4*S*)-2-[2-(4-chlorophenyl)ethyl]-2-[(1*H*-imidazol-1-yl)methyl]-4[((5-trifluoromethylpyridin-2-yl)thio)methyl]-1,3-dioxolane) PDB code of the HO-1. AutoGrid (4.2.6) was used to generate the grid maps with a spacing of 0.375 Å and dimensions encompassing all atoms extending 5 Å from the surface of the PM3 minimized structure of the crystallized ligand. All the parameters were inserted at their default settings. In the docking tab, the macromolecule and ligand are selected, and GA parameters are set as ga_runs = 100, ga_pop_size = 150, ga_num_evals = 20,000,000, ga_num_generations = 27,000, ga_elitism = 1, ga_mutation_rate = 0.02, ga_crossover_rate = 0.8, ga_crossover_mode = two points, ga_cauchy_alpha = 0.0, ga_cauchy_beta = 1.0, number of generations for picking worst individual = 10. The QSAR evaluation was achieved using Forge (v. 10.4.2) after alignment with the dataset in the 3D-QSAR model already published [37]. The field points of all the molecules evaluated in the QSAR model (negative, positive, shape, and hydrophobic) were calculated and generated using the XED (extended electron distribution) force field in Forge; then, the molecules were aligned with the training set of the QSAR model by a maximum common substructure algorithm using a customized and validated set-up [37,47,48]. All the specifications used for the conformation hunt and alignment are detailed in the Supplementary Material (Figures S21 and S22). The maximum number of conformations created for each molecule was set to 500. The root mean square deviation of the atomic positions’ cutoff, which is the similarity threshold below which two conformers are assumed identical, was set to 0.5 Å. The gradient cutoff for conformer minimization was set to 0.1 kcal/mol. The energy window was set to 2.5 kcal/mol, and all the conformers with calculated energy outside the selected energy window were rejected.

### 3.3. Biology

#### 3.3.1. Preparation of Spleen Microsomal Fractions

Rat spleen microsomal fraction obtained by differential centrifugation, according to Ryter et al. [49], was used as source of the HO-1 protein. As reported in the literature [50], HO-1 is abundant in the rat spleen. We chose this source because it represents the most native (i.e., closest to in vivo) form of HO-1. The experiments reported in the present paper complied with current Italian law and met the guidelines of the Institutional Animal Care and Use Committee of the Ministry of Health (Directorate-General for Animal Health and Veterinary Medicines, Italy) “Dosing of enzymatic activities in rat microsomes” (2018–2022), project code 02769.N.VLY. We used male albino rats of strain Sprague–Dawley (150 g body weight and age 45 d) to obtain spleen microsomal fraction. Rats had free access to water and were kept at room temperature with a natural photoperiod (12 h light–12 h dark cycle). Each rat was sacrificed, and their spleens were pooled and then homogenized in 50 mM Tris buffer, pH 7.4, containing 0.25 M sucrose, using a Potter–Elvehjem homogenizing system with a Teflon pestle. Rat spleen homogenate was centrifuged at 10,000× *g* for 20 min at 4 °C, and the supernatant was centrifuged at 100,000× *g* for 60 min at 4 °C. The 100,000 g pellet (microsomes) was resuspended in 100 mM potassium phosphate buffer, pH 7.8, containing MgCl_2_ 2 mM. After measuring its protein concentration, the microsomal fraction was aliquoted and stored at −80 °C for up to 2 months.

#### 3.3.2. Preparation of Biliverdin Reductase

Liver tissue was used to obtain biliverdin reductase. Liver tissue was homogenized on ice in 3 volumes of a solution 1.15% KCl *w*/*v* and Tris buffer 20 mM, pH 7.8. Rat liver homogenate was centrifuged at 10,000× *g* for 20 min at 4 °C, and the supernatant was centrifuged at 100,000× *g* for 1 h at 4 °C. The 100,000 g supernatant was aliquoted and stored at −80 °C after its protein concentration was measured.

#### 3.3.3. Measurement of HO-1 Enzymatic Activity in the Microsomal Fraction of Rat Spleen

Determination of HO-1 activity has been performed by measuring the bilirubin formation using the difference in absorbance at 464–530 nm as described by Ryter et al. [49]. The incubation mixture contained the following in a final volume of 500 µL: 20 mM Tris–HCl, pH 7.4, (1 mg/mL) microsomal extract, 0.5–2 mg/mL biliverdin reductase, 1 mM NADPH, 2 mM glucose 6-phosphate (G6P), 1 U G6P dehydrogenase, 25 µM hemin, and 10 µL of DMSO (or the same volume of DMSO solution of test compounds to a final concentration of 100, 10, and 1 µM). Enzymatic reactions were carried out in the dark for 1 h at 37 °C in a circulating water bath. Then, 500 µL of chloroform was used to stop the reaction. The amount of bilirubin formed was measured in the chloroform phase, with a double-beam spectrophotometer as OD464–530 nm (extinction coefficient, 40 mM/cm for bilirubin). The enzymatic activity was defined as Units (one Unit is the amount of enzyme catalyzing the formation of 1 nmol of bilirubin/mg protein/h).

## 4. Conclusions

In this paper, we reported a computer-aided fragment-based ligand-joining experiment inside the binding pocket of the HO-1 starting from the imidazole of the eastern region and a benzyl group located far in the western region of the HO-1 pocket. Then, the two structures were virtually joined, and 1000 new compounds were generated and evaluated by docking calculation and scoring after alignment in our previously published 3D QSAR model. Five compounds were selected among the 50 best-scored compounds by fingerprint similarity analysis and synthesized along with the other three compounds. The measured IC_50_ against HO-1 confirmed the molecules’ activity, and molecules with the methyl substituent at the nitrogen atom of the indole were generally more potent than benzyl-substituted compounds. Moreover, molecule **4d** showed an IC_50_ in the low micromolar range as predicted by the calculation. Considering the success of this approach, we will extend this work by synthesizing some of the other most potent derivatives found out by the virtual joining experiments. We will also use other fragment-based computer-aided experiments to individuate other hit compounds.

## Figures and Tables

**Table 1:** General structure and experimental IC_50_ values of compounds **1**, **4a**–**d**, **8a**–**c**, and Azalanstat.

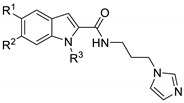					
Compound	R^1^	R^2^	R^3^	HO-1 In Silico (μM) ^a^	HO-1 IC_50_ (μM) ^b^
**1**	PhCH_2_O	H	H	1.19	89.60 ± 6.10
**4a**	CH_3_	H	CH_3_	—	367.31 ± 34.50
**4b**	OCH_3_	H	CH_3_	—	113.33 ± 18.50
**4c**	OCH_3_	OCH_3_	CH_3_	—	55.48 ± 3.82
**4d**	PhCH_2_O	H	CH_3_	1.03	1.03 ± 0.13
**8a**	CH_3_	H	PhCH_2_	1.49	2063 ± 412
**8b**	OCH_3_	H	PhCH_2_	1.29	349.24 ± 37.00
**8c**	OCH_3_	OCH_3_	PhCH_2_	1.30	81.03 ± 4.65
Azalanstat ^c^	—	—	—	—	5.30 ± 0.40

^a^ In silico average calculated activity for the virtually evaluated compounds (*K*_i_ from docking and IC_50_ from QSAR). ^b^ Data are shown as IC_50_ values in μM ± standard deviation (SD) and are the mean of triplicate experiments. ^c^ Data taken from ref. [33].

## Data Availability

The data has been present in main text and supplementary material.

## Supplementary Materials

The following are available online at https://www.mdpi.com/article/10.3390/ph14121289/s1, Figure S1: ^1^H NMR (500 MHz, DMSO-*d*_6_) of compound **1**; Figure S2: ^13^C NMR (125 MHz, DMSO-*d*_6_) of compound **1**; Figure S3: ^1^H NMR (500 MHz, DMSO-*d*_6_) of compound **4a**; Figure S4: ^13^C NMR (125 MHz, DMSO-*d*_6_) of compound **4a**; Figure S5: ^1^H NMR (500 MHz, DMSO-*d*_6_) of compound **4b**; Figure S6: ^13^C NMR (125 MHz, DMSO-*d*_6_) of compound **4b**; Figure S7: ^1^H NMR (500 MHz, DMSO-*d*_6_) of compound **4c**; Figure S8: ^13^C NMR (125 MHz, DMSO-*d*_6_) of compound **4c**; Figure S9: ^1^H NMR (500 MHz, DMSO-*d*_6_) of compound **4d**; Figure S10: ^13^C NMR (125 MHz, DMSO-*d*_6_) of compound **4d**; Figure S11: ^1^H NMR (500 MHz, DMSO-*d*_6_) of compound **8a**; Figure S12: ^13^C NMR (125 MHz, DMSO-*d*_6_) of compound **8a**; Figure S13: ^1^H NMR (500 MHz, DMSO-*d*_6_) of compound **8b**; Figure S14: ^13^C NMR (125 MHz, DMSO-*d*_6_) of compound **8b**; Figure S15: ^1^H NMR (500 MHz, DMSO-*d*_6_) of compound **8c**; Figure S16: ^13^C NMR (125 MHz, DMSO-*d*_6_) of compound **8c**; Figure S17: Docked pose of **1** (light pink) and **4d** (blue) inside HO-1; Figure S18: Docked pose of **4a** (light pink), **4b** (green) and **4c** (blue) inside HO-1; Figure S19: Docked pose of **8a** (blue), **8b** (light pink), and **8c** (green) inside HO-1; Figure S20: Spark’s libraries used for the growing experiments; Figure S21: Forge’s parameters used for the conformation hunt; Figure S22: Forge’s parameters used for the alignment; Table S1: *K*_i_ μM from docking, IC_50_ μM from QSAR and average μM calculated activity for the virtually evaluated compounds; Table S2: ECPF4 fingerprint similarity matrix values; Table S3: ECPF4 fingerprint similarity matrix values.
