# Supplementary material for: From Far West to East: Joining the Molecular Architecture of Imidazole-like Ligands in HO-1 Complexes
Source: Pharmaceuticals (Basel). 2021 Dec 10;14(12):1289. doi: 10.3390/ph14121289 (PMC8704944; doi:10.3390/ph14121289)
Supplement: Supplementary file 1 [file pharmaceuticals-14-01289-s001.zip › pharmaceuticals-1489749-supplementary.pdf]

Supplementary Material

From Far West to East: Joining the Molecular Architecture of Imidazole-like Ligands in HO-1 Complexes

Giuseppe Floresta [1,2,†], Antonino Nicolò Fallica [1,†], Vincenzo Patamia [1], Valeria Sorrenti [1], Khaled Greish [3], Antonio Rescifina [1,*] and Valeria Pittalà [1,*]

1 Department of Drug and Health Sciences, University of Catania, V.le A. Doria 6, 95125 Catania, Italy; giuseppe.floresta@kcl.ac.uk (G.F.); antonino.fallica@phd.unict.it (A.N.F.); vincenzo.patamia@unict.it (V.P.); sorrenti@unict.it (V.S.)

2 Department of Analytics, Environmental & Forensics, King's College London, London SE1 9NH, UK

3 Department of Molecular Medicine and Nanomedicine Unit, Princess Al-Jawhara Center for Molecular Medicine, College of Medicine and Medical Sciences, Arabian Gulf University, Manama 329, Bahrain; khaledfg@agu.edu.bh

* Correspondence: arescifina@unict.it (A.R.); vpittala@unict.it (V.P.)

† These authors contributed equally.

Table of contents

Figures S1–16 NMR spectra of compounds. S2–S9

Figure S17. Docked pose of **1** (light pink) and **4d** (blue) inside HO-1. S10

Figure S18. Docked pose of **4a** (light pink), **4b** (green) and **4c** (blue) inside HO-1. S10

Figure S19. Docked pose of **8a** (blue), **8b** (light pink) and **8c** (green) inside HO-1. S11

Figure S20. Spark's libraries used for the growing experiments. S12

Figure S21. Forge's parameters used for the conformation hunt. S13

Figure S22. Forge's parameters used for the alignment. S13

Table S1. K_i µM from docking, IC_{50} µM from QSAR and Average µM calculated activity for the virtually evaluated compounds. S14

Table S2. ECPF4 fingerprint similarity matrix values. S25

Table S3. ECPF4 fingerprint similarity matrix values. S25

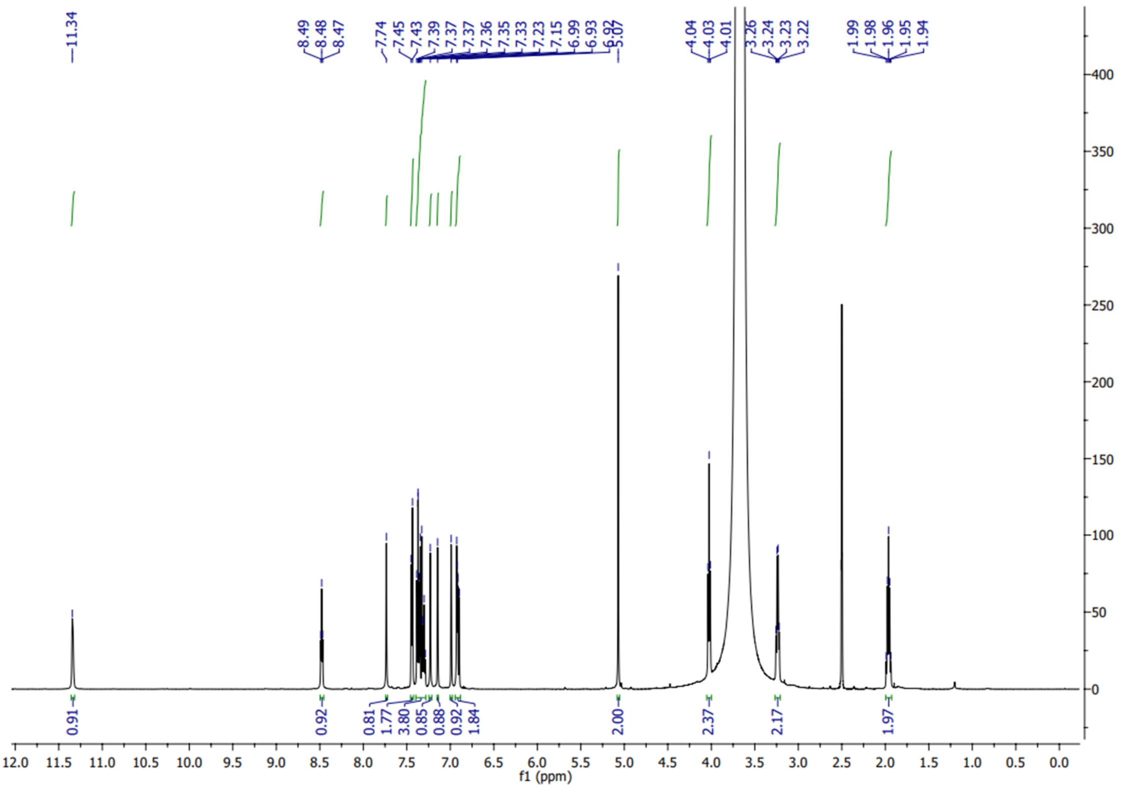

Figure S1. ^{1}H NMR (500 MHz, DMSO-d_6) of compound **1**.

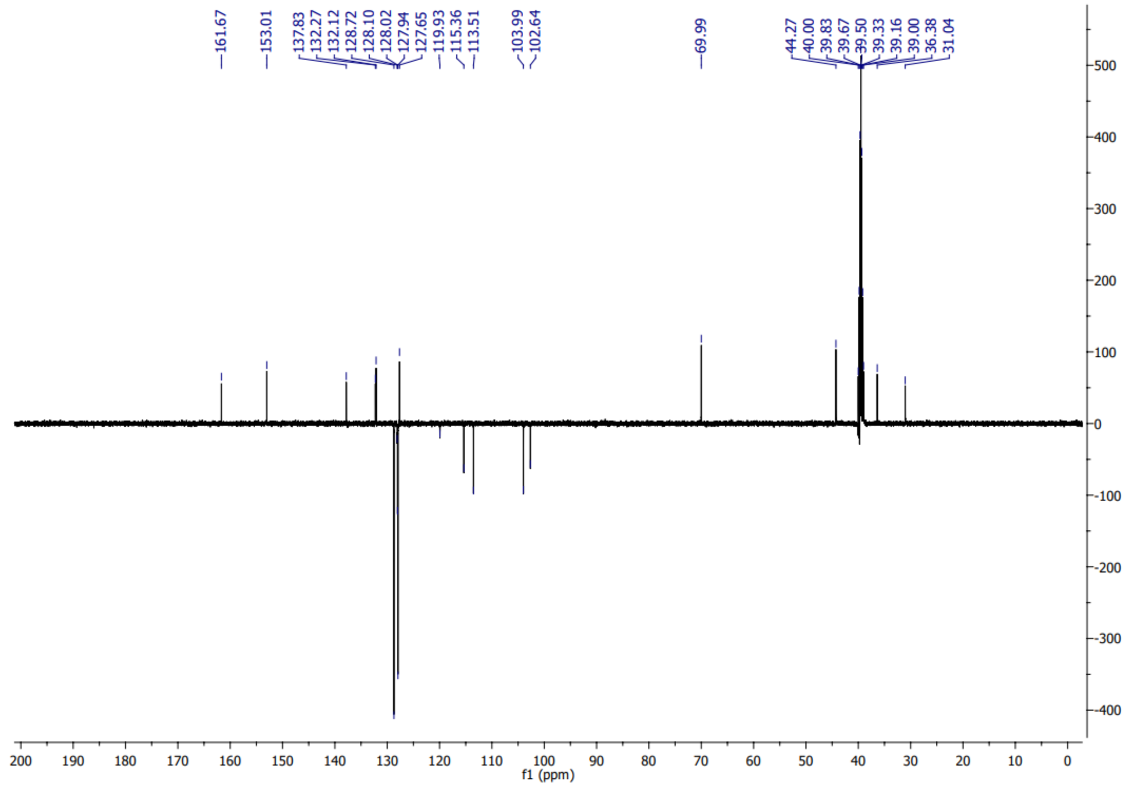

Figure S2. ^{13}C NMR (500 MHz, DMSO-d_6) of compound **1**.

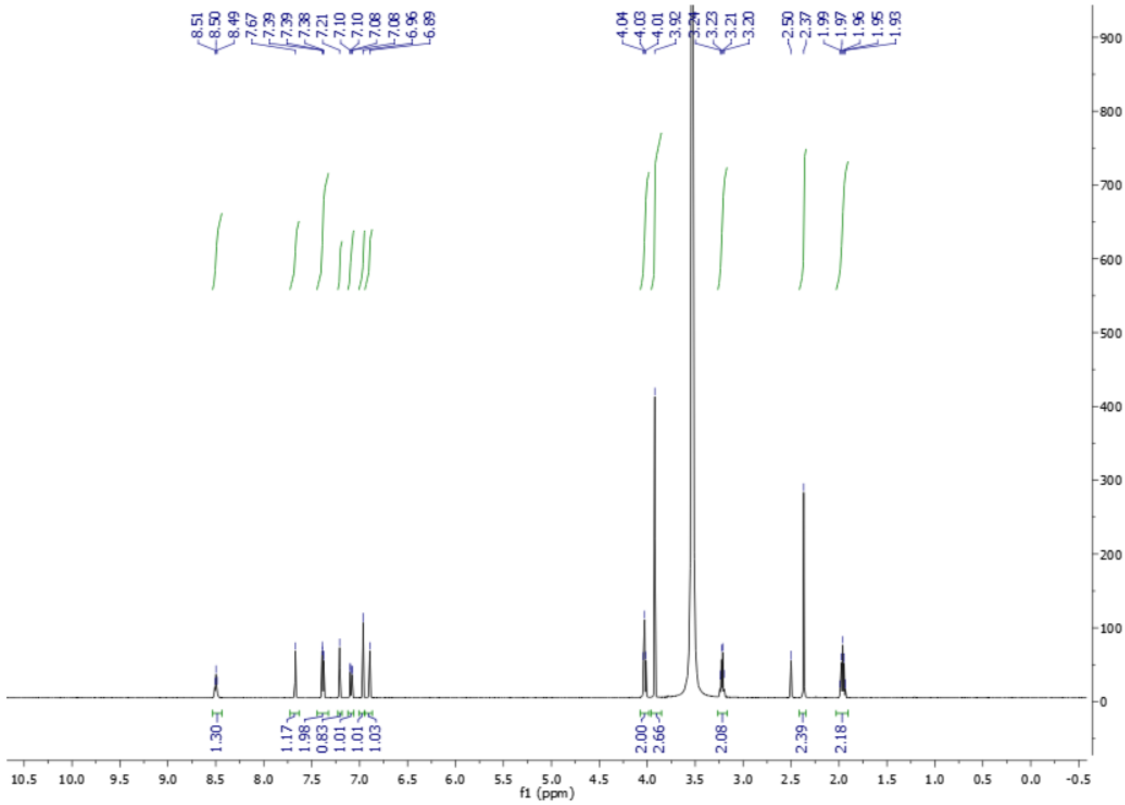

Figure S3. ^{1}H NMR (500 MHz, DMSO-d_6) of compound **4a**.

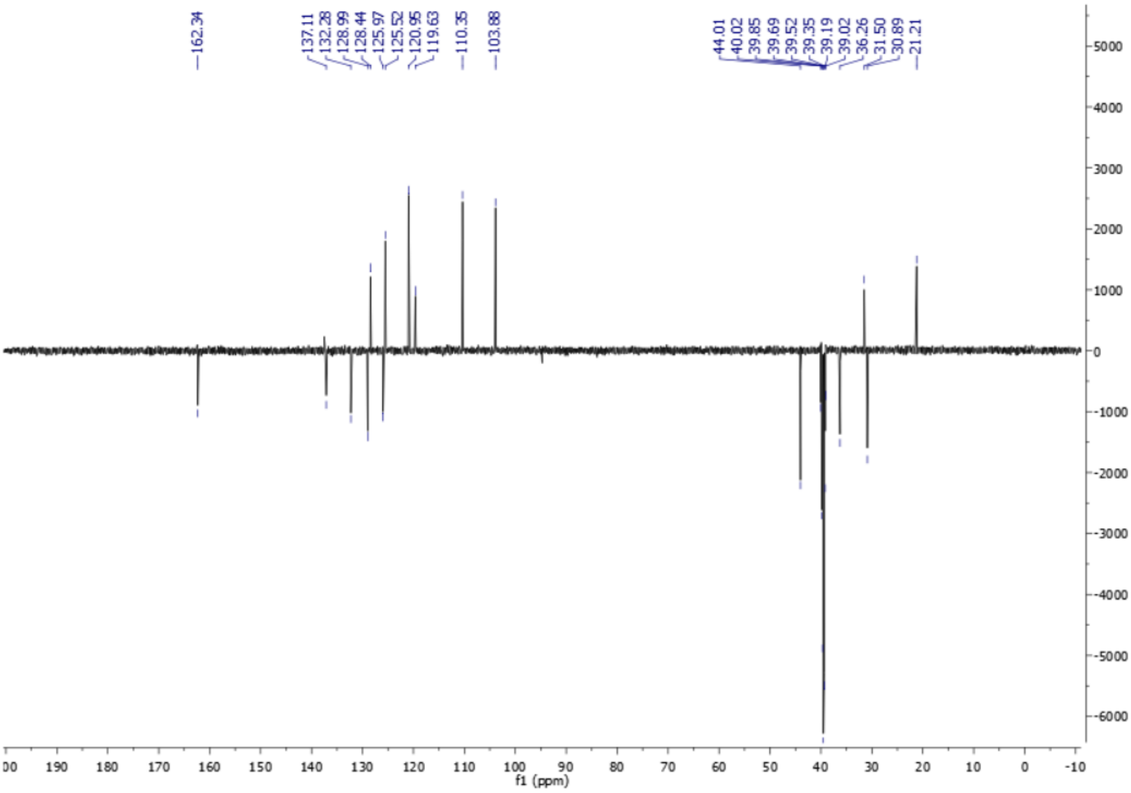

Figure S4. ^{13}C NMR (125 MHz, DMSO-d_6) of compound **4a**.

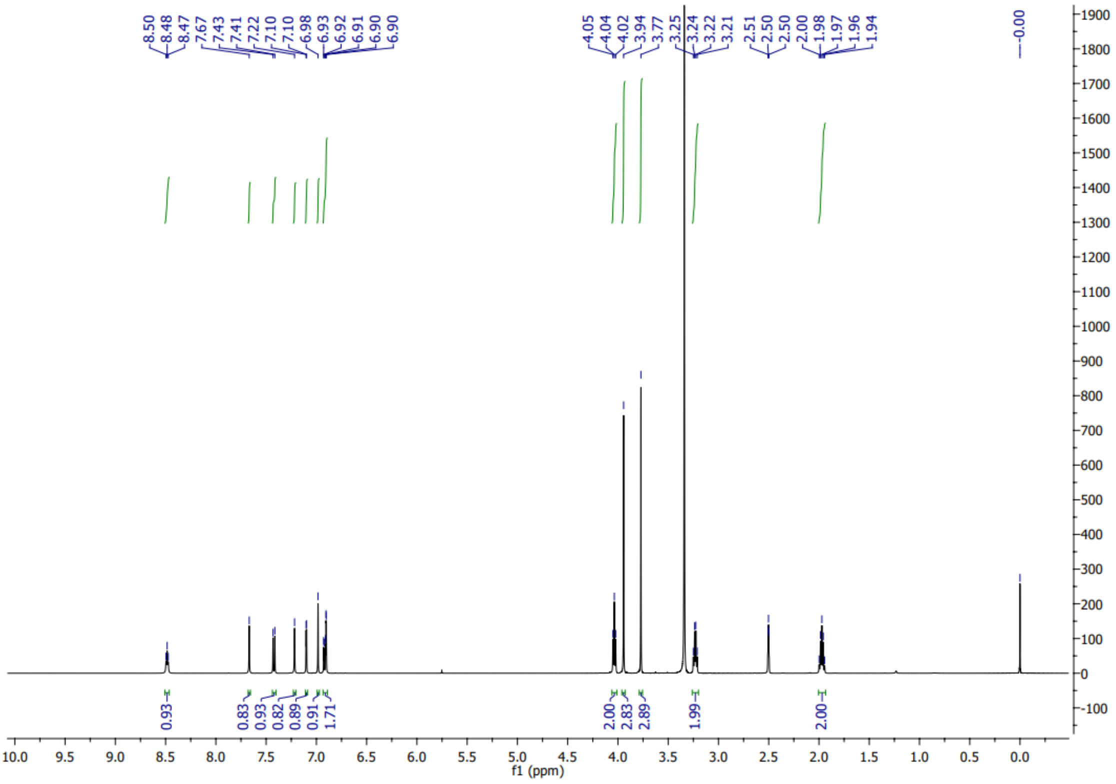

Figure S5. ^{1}H NMR (500 MHz, DMSO-d_6) of compound **4b**.

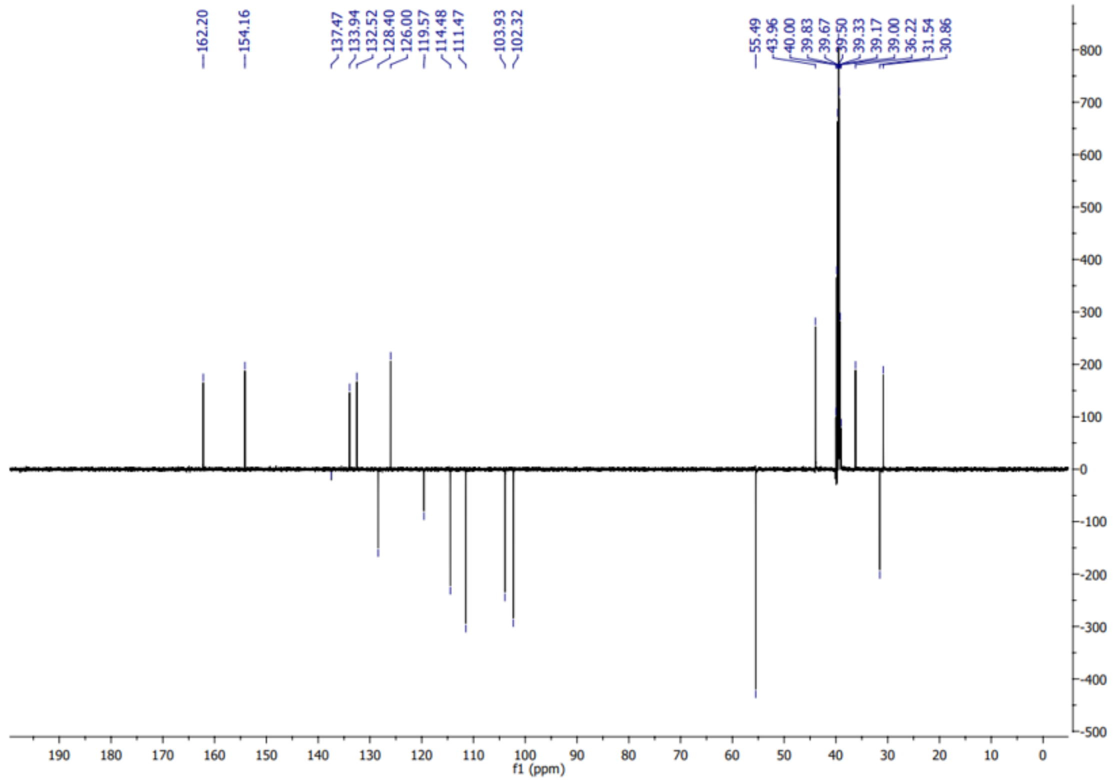

Figure S6. ^{13}C NMR (125 MHz, DMSO-d_6) of compound **4b**.

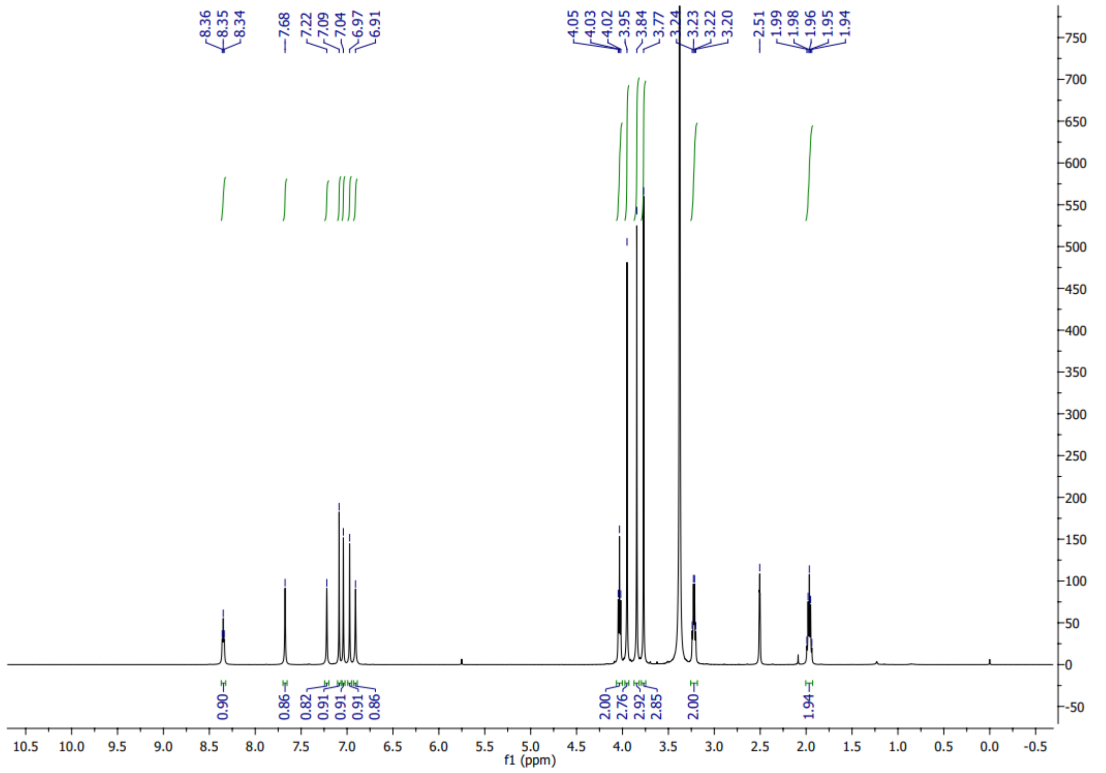

Figure S7. ^{1}H NMR (500 MHz, DMSO-d_6) of compound **4c**.

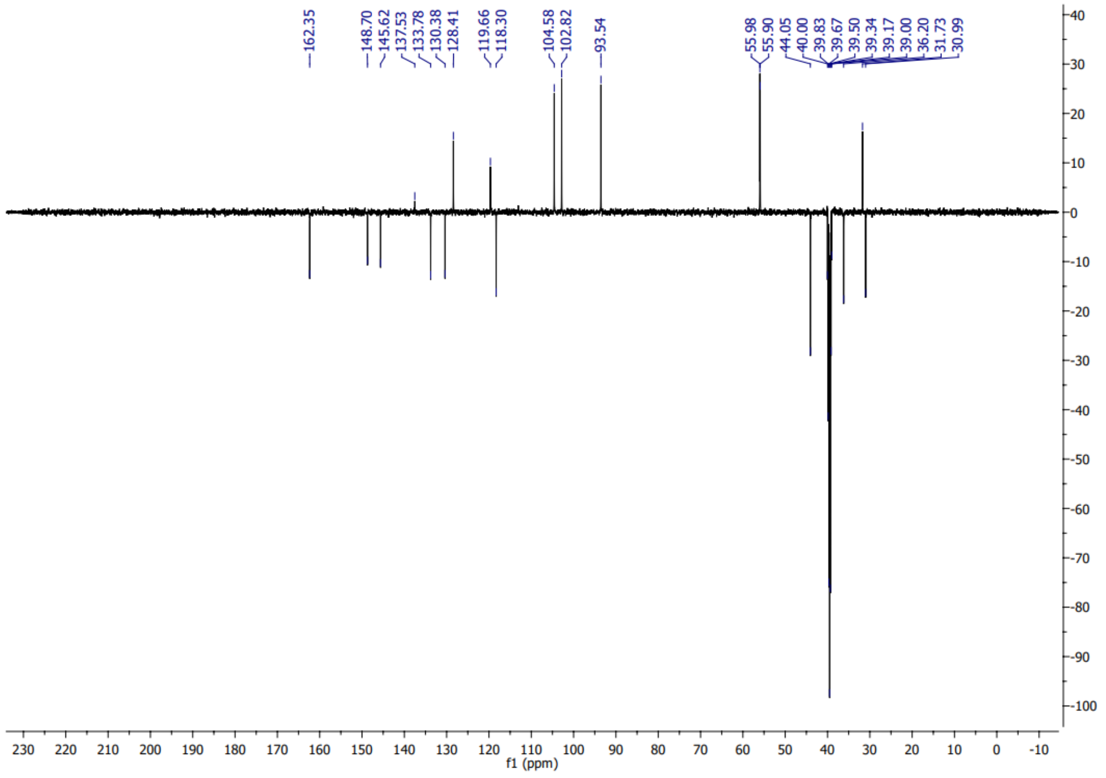

Figure S8. ^{13}C NMR (125 MHz, DMSO-d_6) of compound **4c**.

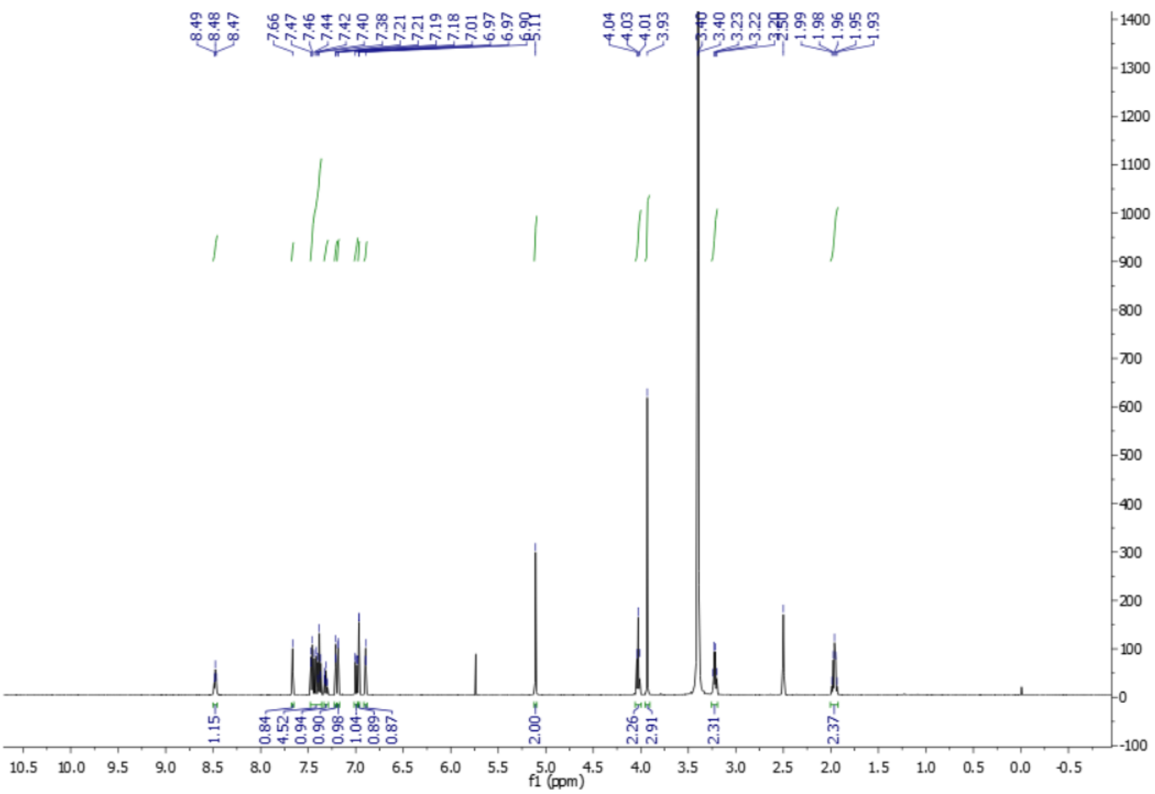

Figure S9. ^{1}H NMR (500 MHz, DMSO-d_6) of compound **4d**.

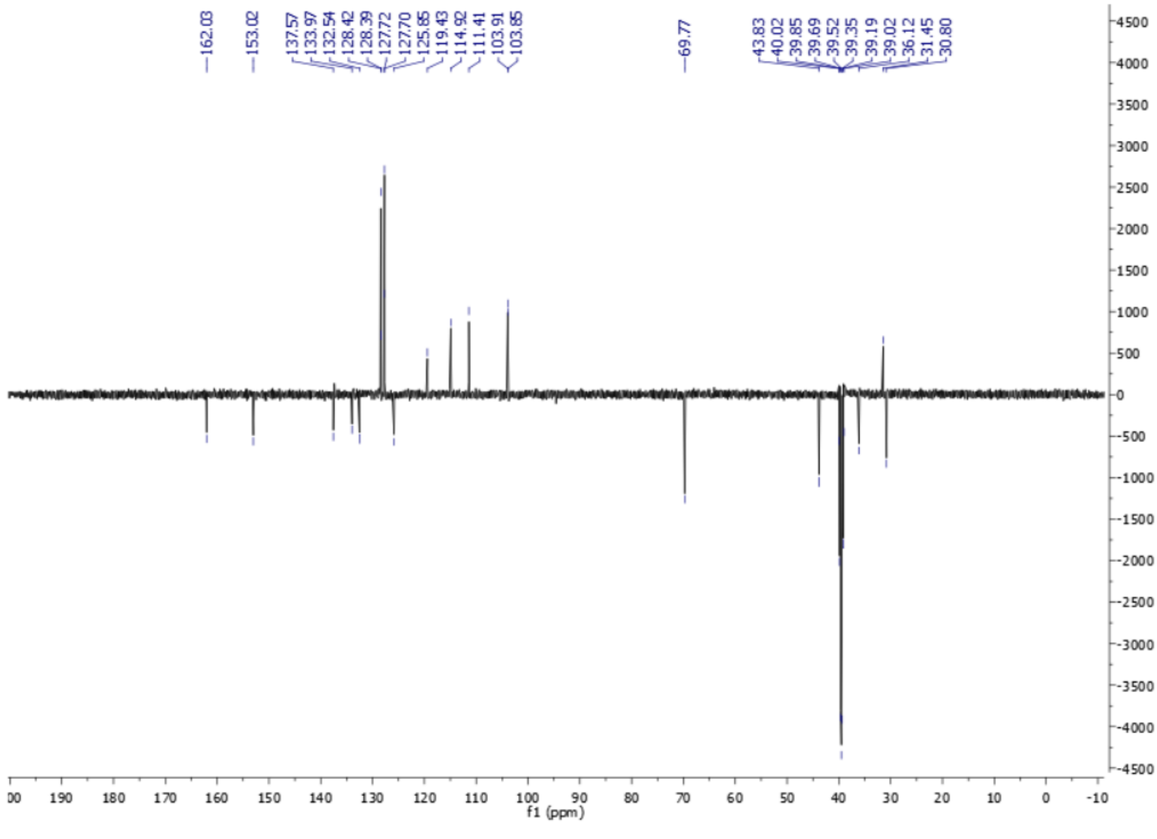

Figure S10. ^{13}C NMR (125 MHz, DMSO-d_6) of compound **4d**.

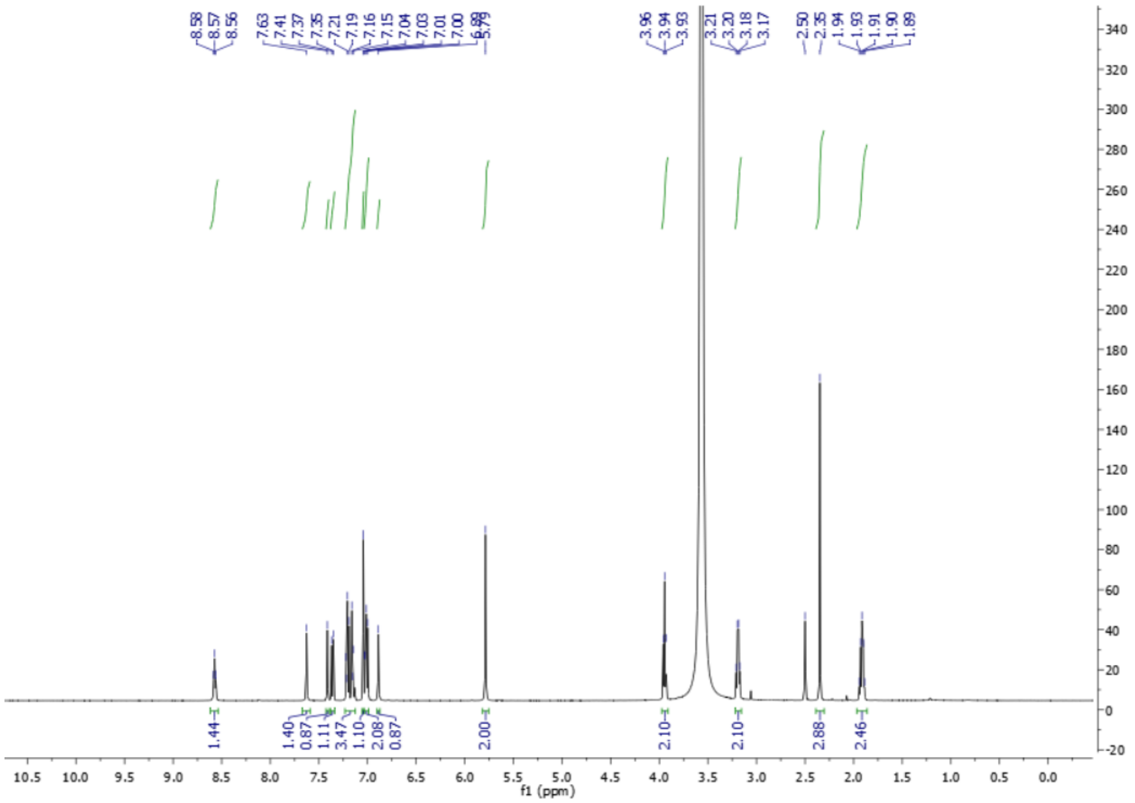

Figure S11. ^{1}H NMR (500 MHz, DMSO-d_6) of compound **8a**.

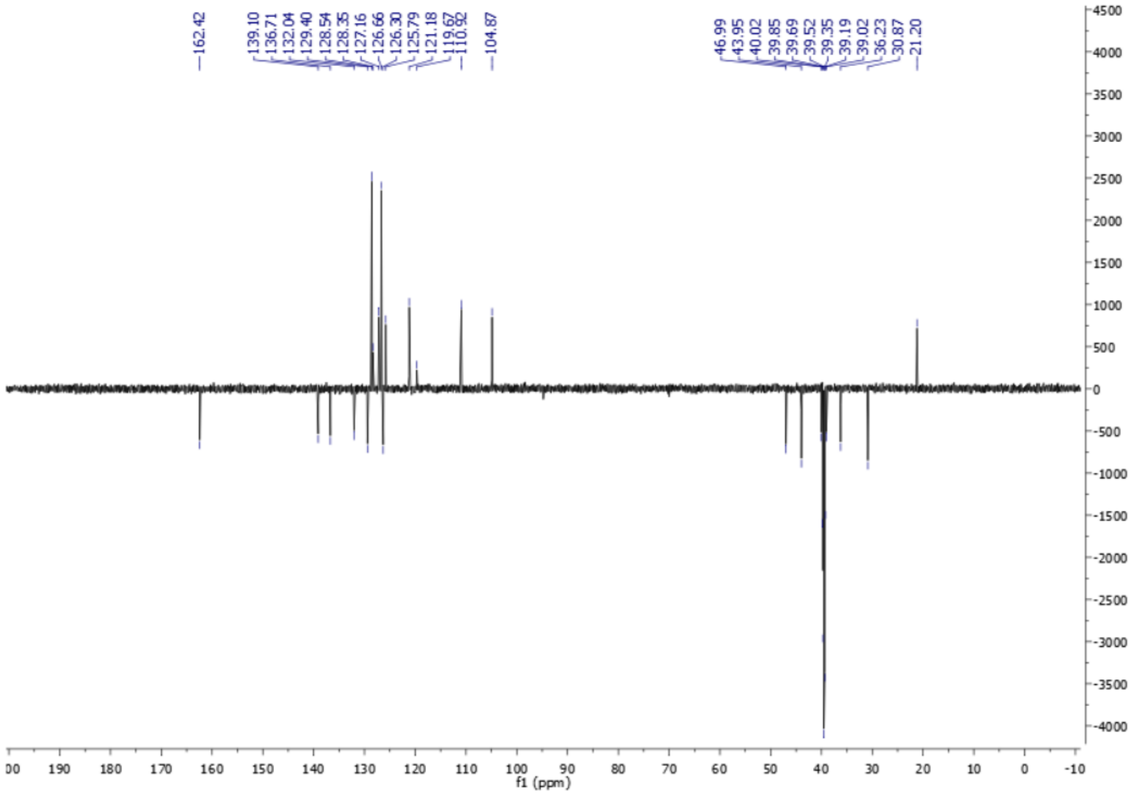

Figure S12. ^{13}C NMR (125 MHz, DMSO-d_6) of compound **8a**.

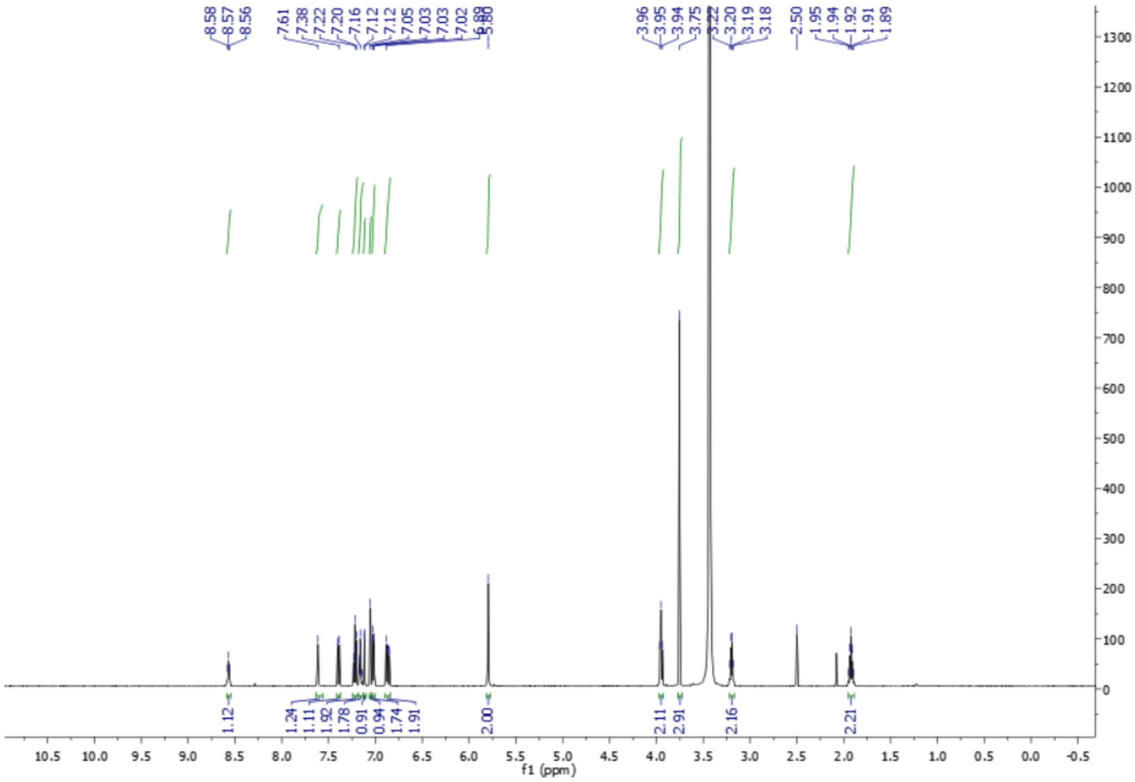

Figure S13. ^{1}H NMR (500 MHz, DMSO-*d*6) of compound **8b**.

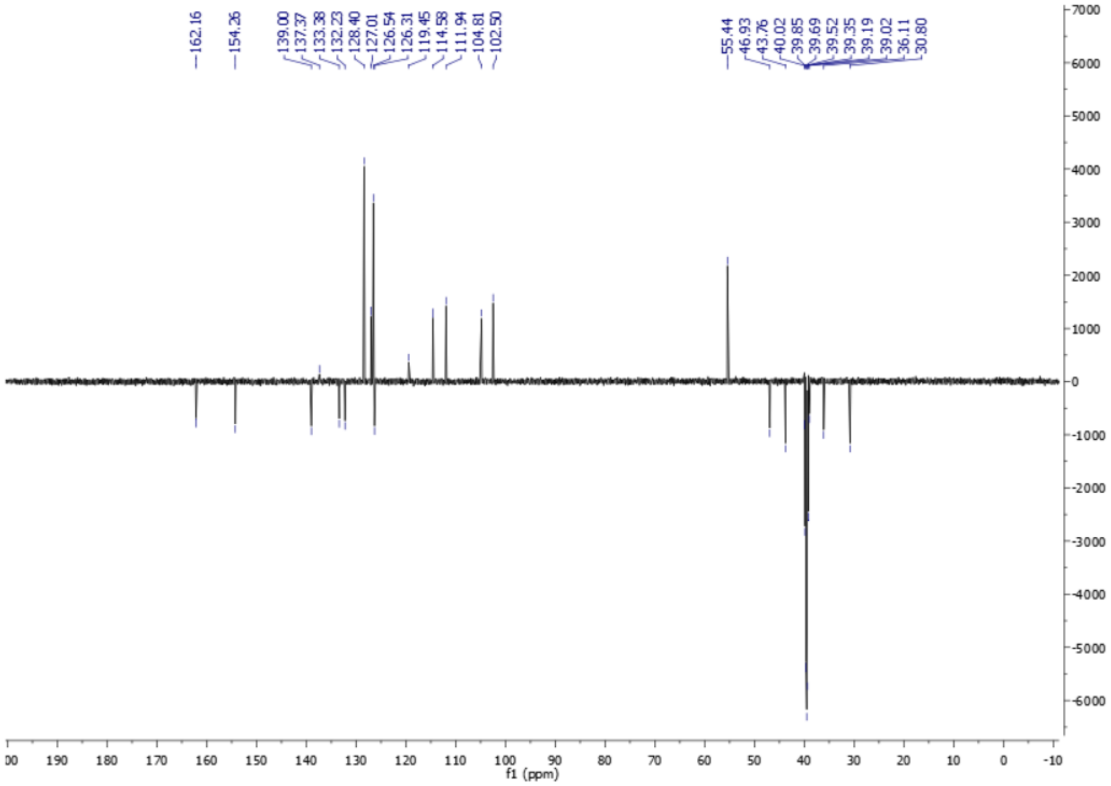

Figure S14. ^{13}C NMR (125 MHz, DMSO-*d*6) of compound **8b**.

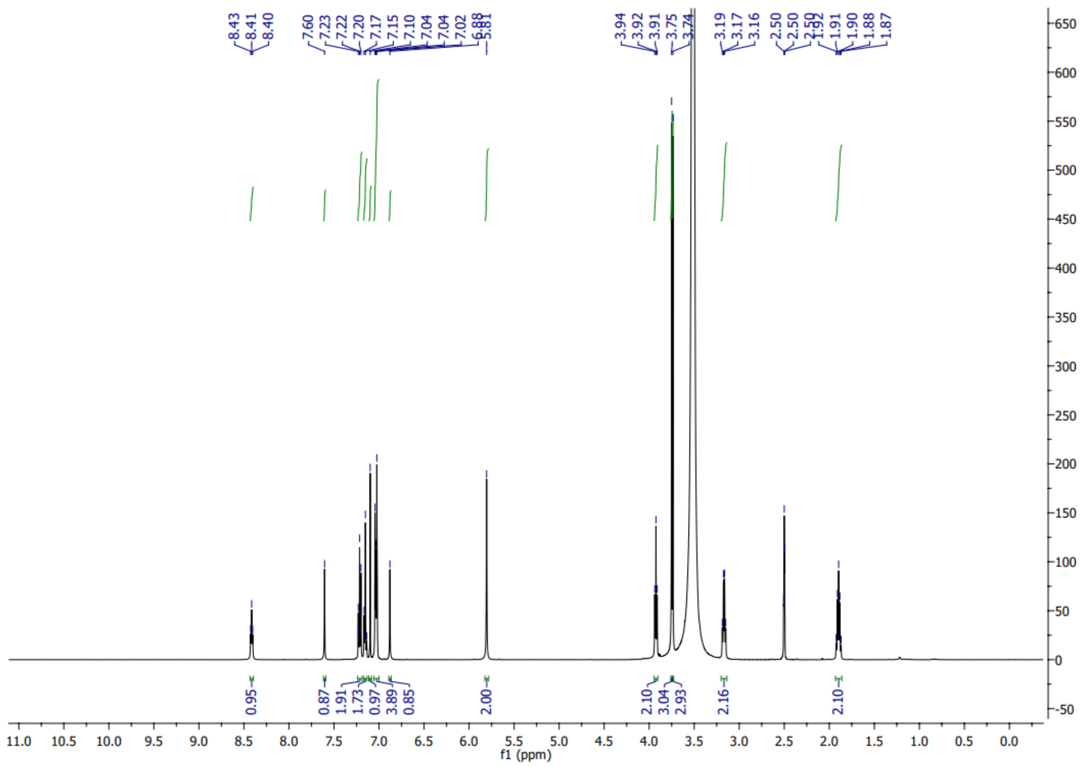

Figure S15. ^{1}H NMR (500 MHz, DMSO-d_6) of compound **8c**.

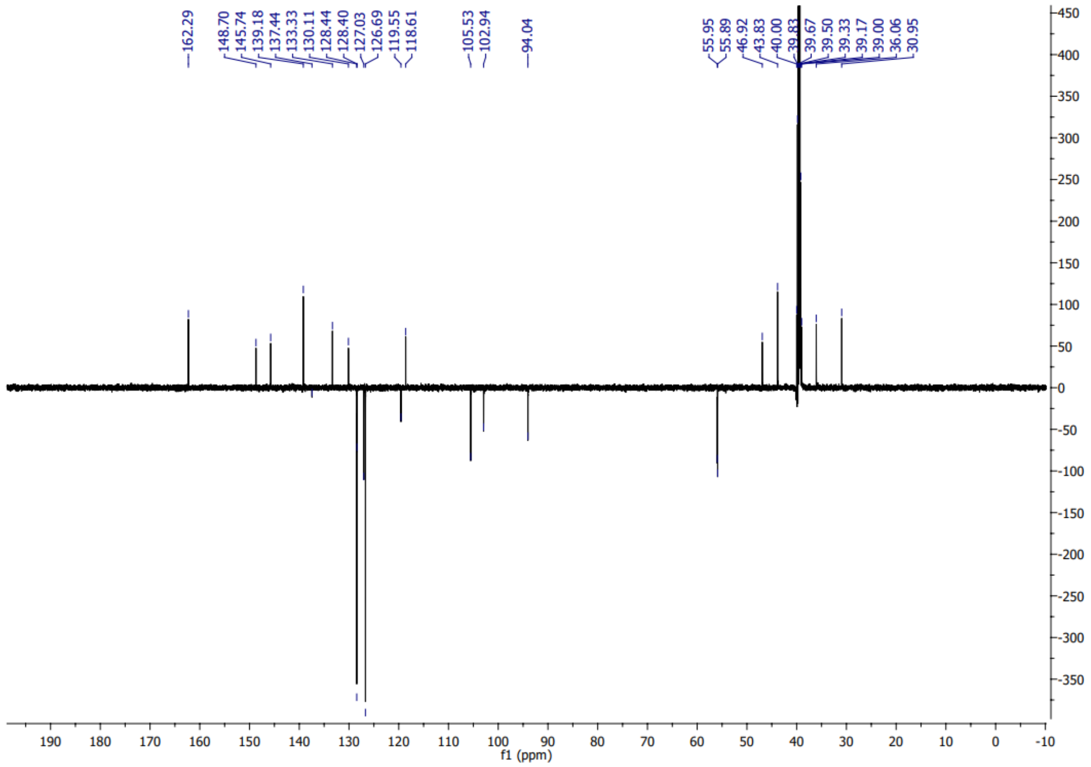

Figure S16. ^{13}C NMR (125 MHz, DMSO-d_6) of compound **8c**.

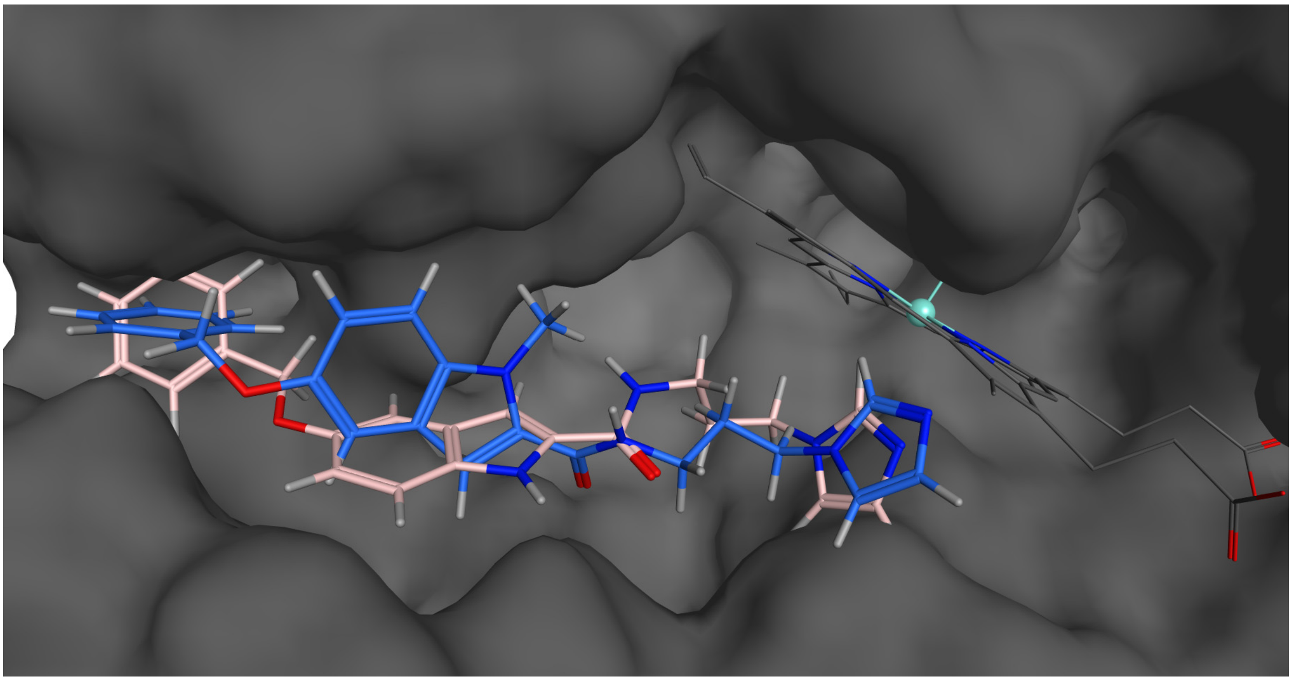

Figure S17. Docked pose of **1** (light pink) and **4d** (blue) inside HO-1.

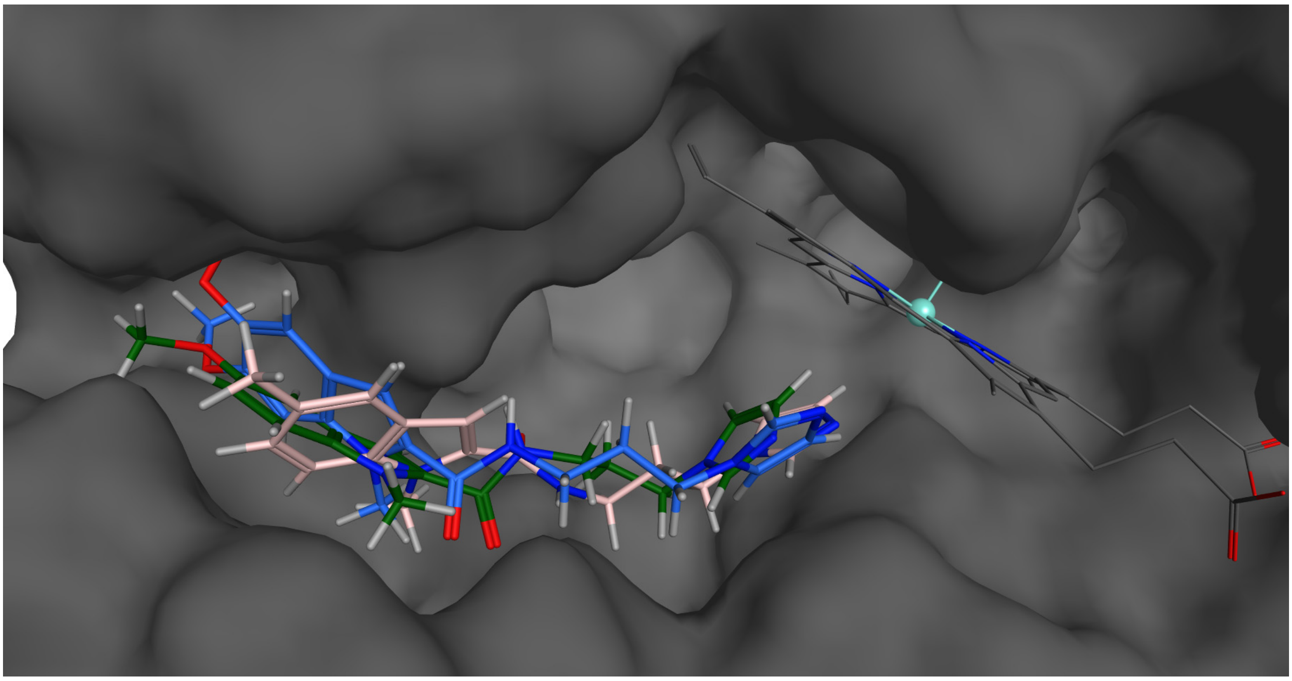

Figure S18. Docked pose of **4a** (light pink), **4b** (green) and **4c** (blue) inside HO-1.

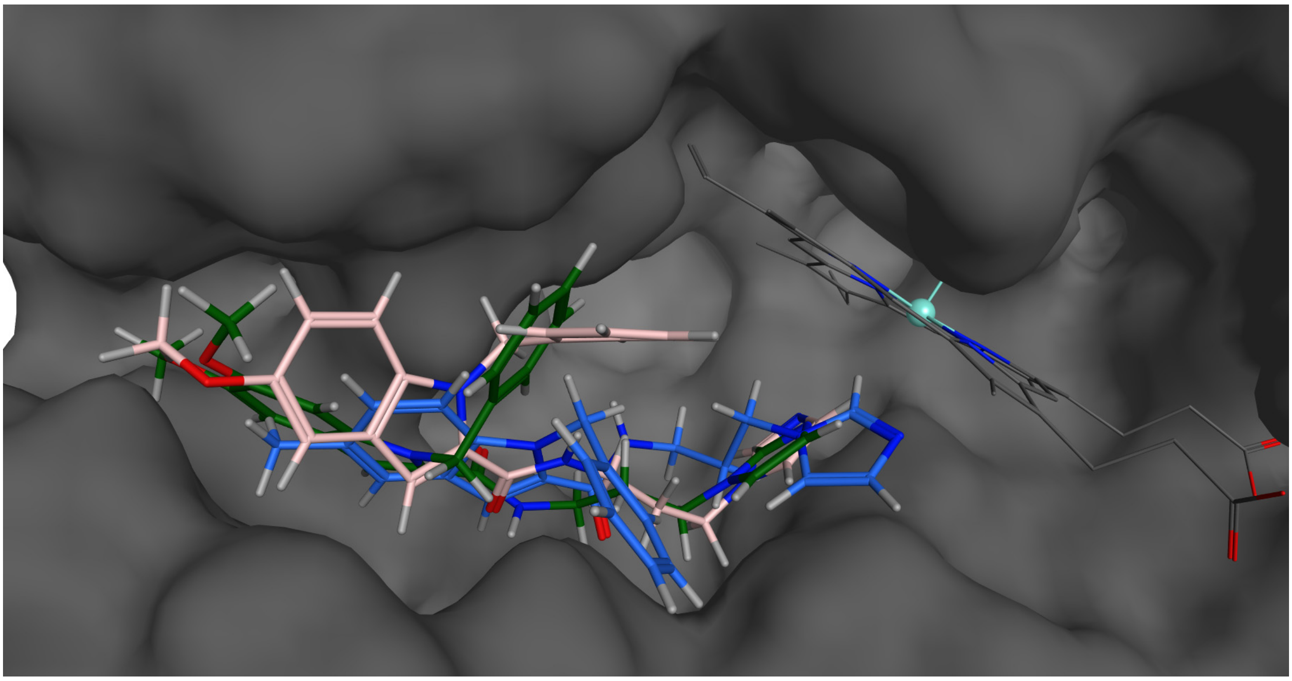

Figure S19. Docked pose of **8a** (blue), **8b** (light pink) and **8c** (green) inside HO-1.

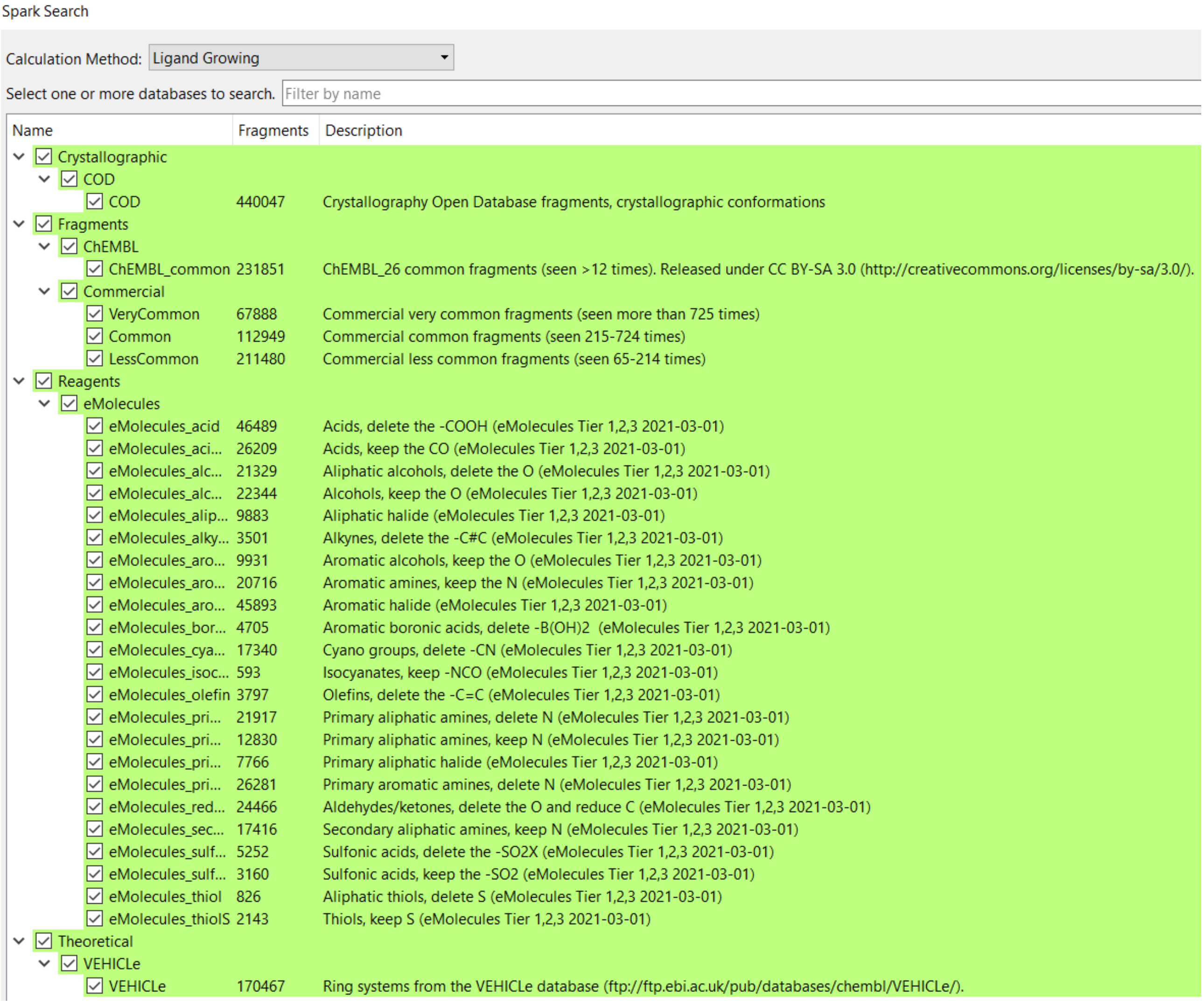

Figure S20. Spark's libraries used for the growing experiments.

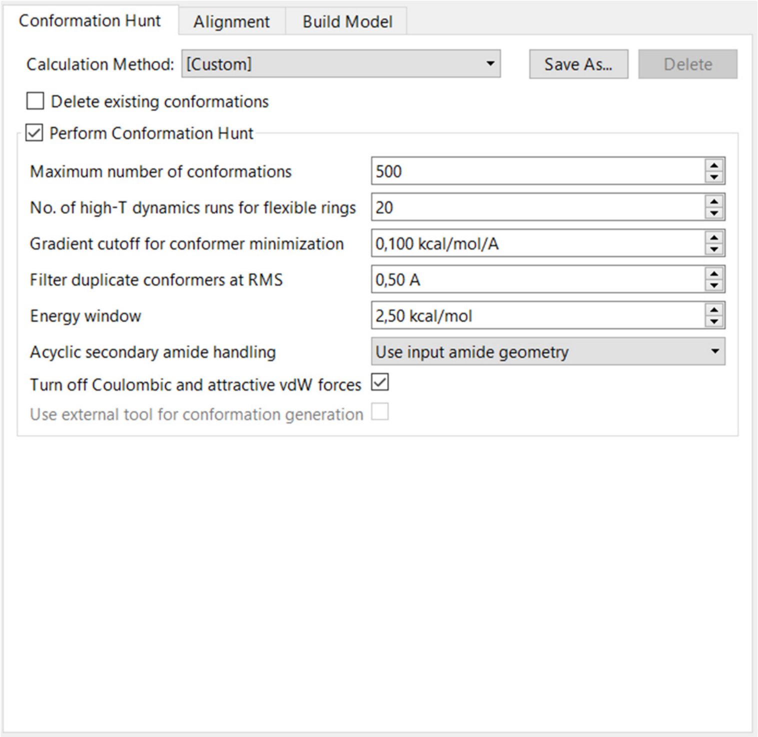

Figure S21. Forge's parameters used for the conformation hunt.

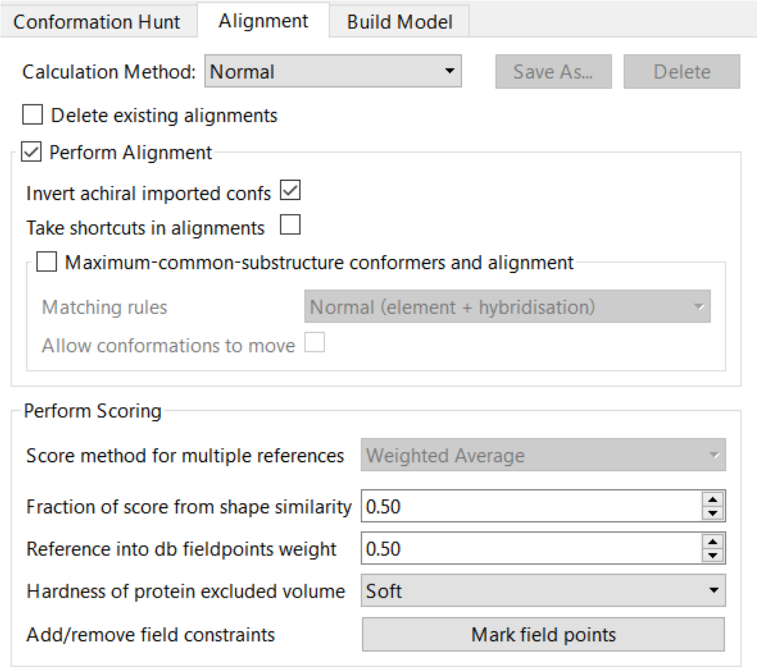

Figure S22. Forge's parameters used for the alignment.

Table S1. K_i µM from docking, IC_{50} µM from QSAR and average µM calculated activity for the virtually evaluated compounds (cut-off at 10 µM average).

Structure	K_i µM from docking	IC_{50} µM from QSAR	Average µM
s1c(-c2noc(n2)-c3ccccc3)ccc1CNn4ccnc4	0.44	1.58	1.01
O=C(Nc1nc(-c2ccccc2)cs1)C3CCN(n4ccnc4)CC3	0.46	1.58	1.02
O=C(NCCCn1cncc1)c2cc3cc(OCc4ccccc4)ccc3n2C (compound **4d**)	1.26	0.79	1.03
O=C(NCCCn1cncc1)/C=C/Nc2ccc(-c3ccccc3)cc2	0.47	1.58	1.03
O=C(Nc1cc(n[nH]1)-c2ccccc2)c3ccc(-n4ccnc4)cc3	0.13	2.00	1.06
O=C(N[C@@H]1COC2(C1)CC[NH+](CC2)Cn3cncc3)c4ccccc4	0.88	1.26	1.07
O=C(NCCCn1cncc1)c2ccc(C3CCN(CC3)c4ccccc4)cc2	0.15	2.00	1.07
O=C(Nc1ccccc1)c2cc([nH]n2)-c3ccc(s3)-n4ccnc4	0.17	2.00	1.08
O=C(NCCCn1cncc1)[C@@H]2CCc3ccc(Oc4ccccc4)cc3C2	0.17	2.00	1.08
O=C(NCc1ccccc1)[C@@H]2CCC3(O2)CCN(n4ccnc4)CC3	0.18	2.00	1.09
Clc1cc(-c2cc(no2)C(=O)NCCCn3cncc3)ccc1-c4ccccc4	0.20	2.00	1.10
O=C(NCCCn1cncc1)CSc2ccc(-c3ccccc3)cn2	1.41	0.79	1.10
O=C(NCCCn1cncc1)c2ccc(s2)-c3ccc(o3)-c4ccccc4	0.21	2.00	1.10
O=C1C(Oc2ccccc2)=COC(COC(=O)NCCCn3cncc3)=C1	1.42	0.79	1.11
O=C(Nn1ccnc1)C2CCN(CC2)c3nc(no3)-c4ccccc4	0.23	2.00	1.11
O=C(NCCn1cncc1)[C@@H]2CCN(CCC2)c3ccccc3	1.25	1.00	1.13
O=C(NC[C@@H]1Cc2cc(-c3ccccc3)ccc2O1)NCCCn4cncc4	0.26	2.00	1.13
[nH+]1cc(ccc1N2CCC(Nn3ccnc3)CC2)-c4ccccc4	0.35	2.00	1.17
O=C(Nn1ccnc1)c2cncc(c2)-c3cnc(s3)-c4ccccc4	0.38	2.00	1.19
O=C(N1CCc2ccc(Oc3ccccc3)cc2C1)NCCCn4cncc4	0.38	2.00	1.19
O=C(NCCCn1cncc1)c2cc3cc(OCc4ccccc4)ccc3[nH]2 (compound **1**)	0.39	2.00	1.19
O=S(=O)(c1ccc(s1)CNC(=O)NCCCn2cncc2)c3ccccc3	0.80	1.58	1.19
O=C(Nc1nc2c(s1)CN(CC2)c3ccccc3)Cn4cncc4	0.42	2.00	1.21
O=C(N[C@@H]1CCN(C1)C(=O)NCCCn2cncc2)Cc3ccccc3	1.17	1.26	1.21
O=C(NCCCn1cncc1)[C@H](Sc2ccc(-c3ccccc3)cn2)C	0.87	1.58	1.23
O=C(NCCc1ccc(cc1C)-c2ccccc2)NCCCn3cncc3	0.47	2.00	1.23
O=C(Nn1ccnc1)c2ccc(-c3nc(no3)-c4ccccc4)cc2	0.07	2.51	1.29
O=C(NCCCn1cncc1)c2cc3cc(OC)ccc3n2Cc4ccccc4 (compound **8b**)	0.07	2.51	1.29
S(CCCn1cncc1)c2ccc(-c3ccccc3)cc2	1.33	1.26	1.29
o1c(cc2c1ccc(N3CCN(n4ccnc4)CC3)c2)-c5ccccc5	0.08	2.51	1.30
O=C(NCCCn1cncc1)c2cc3cc(OC)c(OC)cc3n2Cc4ccccc4 (compound **8c**)	0.09	2.51	1.30
O=C(On1ccnc1)c2ccc(-c3noc(n3)-c4ccccc4)cc2	0.11	2.51	1.31
s1c(Nn2ccnc2)nnc1Nc3nc(-c4ccccc4)cs3	0.16	2.51	1.33
O=C(NCCc1ccc(-c2ccccc2)cc1)NCCCn3cncc3	0.68	2.00	1.34
O=C(NCCCn1cncc1)c2cc(no2)-c3ccc(-c4ccccc4)cc3	0.17	2.51	1.34
O=C(Nn1ccnc1)c2cccc(-n3cc(-c4ccccc4)cn3)c2	0.17	2.51	1.34
O=C(NCCCn1cncc1)c2c(nc(s2)NNC(=O)c3ccccc3)C	0.76	2.00	1.38
O=C(NCCCn1cncc1)c2cn(nn2)-c3ccc(-c4ccccc4)cc3	0.27	2.51	1.39
O=C(N1CCc2cc(Nc3ccccc3)ccc2CC1)NCCCn4cncc4	0.27	2.51	1.39
O=C(NCCCn1cncc1)/C=C/c2ccc3CN(Cc3c2)c4ccccc4	0.28	2.51	1.39
O=C(Nn1ccnc1)c2cc(n[nH]2)-c3ccc(-c4ccccc4)cc3	0.29	2.51	1.40
N1(CCC(CC1)=Cn2cncc2)c3ccc(cc3)-c4ccccc4	0.30	2.51	1.41

O=C(NCCCn1cncc1)[C@H]2CN(CCC2)C(=O)/C=C/c3ccccc3	0.33	2.51	1.42
O=C(Nc1ccc(-n2ccnc2)cn1)[C@H](Sc3ccccc3)C	0.85	2.00	1.42
O=C(NCCCn1cncc1)NCc2ccc(o2)C(Oc3ccccc3)=O	2.05	0.79	1.42
O=C(n1cncc1)Nc2nc(-c3ccc(-c4ccccc4)cc3)c[nH]2	0.37	2.51	1.44
N1(CCC(Nn2ccnc2)CC1)c3ccc(cc3)-c4ccccc4	0.41	2.51	1.46
O=C(N[C@@H]1COc2cc(Oc3ccccc3)ccc2C1)NCCCn4cncc4	0.43	2.51	1.47
O=C(NCCCn1cncc1)c2cc3cc(ccc3n2Cc4ccccc4)C (compound **8a**)	0.47	2.51	1.49
o1c(nc(n1)-c2cc(on2)-n3ccnc3)CNc4ccccc4	0.52	2.51	1.51
O=C(NCCCn1cncc1)CSc2ncc(-c3ccccc3)c(n2)N	2.26	0.79	1.53
s1c(ccc1-n2ccnc2)-c3nnc(o3)CCc4ccccc4	0.55	2.51	1.53
O=C(NC/C=C/n1ccnc1)c2ccc(-c3ccccc3)cc2	0.57	2.51	1.54
O=C(NC1CCN(n2ccnc2)CC1)CSc3ccccc3	1.10	2.00	1.55
N1(CCC(n2cncc2)CC1)c3cnc4ccc(cc4c3)-c5ccccc5	0.01	3.16	1.59
n1(-n2ccnc2)cc(-c3ccc4cnc(cc4c3)-c5ccccc5)cn1	0.02	3.16	1.59
o1c(-c2ccccc2)cc(n1)-c3ccc4c(CCN(n5cncc5)CC4)c3	0.02	3.16	1.59
[nH]1c(nc2cnc(-n3cncc3)cc21)-c4cccc(-c5ccccc5)c4	0.04	3.16	1.60
O=C(Nn1ccnc1)c2csc(n2)C3CCN(CC3)c4ccccc4	0.70	2.51	1.60
O=C(Nc1cnn(-n2cncc2)c1)c3cccc(-c4ccccc4)c3	0.05	3.16	1.61
Clc1cc2c([nH]c(n2)-c3ccccc3)cc1N4CCN(n5ccnc5)CC4	0.06	3.16	1.61
O=C(NCCCn1cncc1)c2ccc(-c3noc(n3)-c4ccccc4)cc2	0.10	3.16	1.63
o1c2cc(-c3ccccc3)ccc2c(n1)C4CCN(n5ccnc5)CC4	0.14	3.16	1.65
O=C(NCCCn1cncc1)CCc2cn(nn2)Cc3ccccc3	2.30	1.00	1.65
O=C(N1CCN(CCC1)c2cc(n[nH]2)-c3ccccc3)NCCCn4cncc4	0.14	3.16	1.65
O=C(NCCCn1cncc1)c2c(oc(n2)-c3cccc(-c4ccccc4)c3)C	0.15	3.16	1.66
O=C(N1CCC(CC1)c2noc(n2)-c3ccccc3)NCCCn4cncc4	0.15	3.16	1.66
N1(CC[C@]2(CCCN(C2)c3ncc(-n4ccnc4)cn3)C1)c5ccccc5	0.24	3.16	1.70
O=C(NC1CC(C1)c2cccc(c2)-c3ccccc3)NCCCn4cncc4	0.27	3.16	1.72
O=C(NCCCn1cncc1)CSc2nnc([nH]2)Cc3ccccc3	2.45	1.00	1.72
O=C(Nn1ccnc1)c2cc([nH]n2)-c3ccc(s3)-c4ccccc4	0.30	3.16	1.73
O=C(N/N=C/c1ccc(o1)-c2ccccc2)Cn3cncc3	0.97	2.51	1.74
O=S(=O)(c1cc(-c2nnc(o2)NCCCn3cncc3)cs1)c4ccccc4	1.49	2.00	1.74
Cc1c(-c2ccccc2)cc[n+](CCCC#Cn3ccnc3)c1	0.36	3.16	1.76
O=C(NCCCn1cncc1)Cc2csc(n2)CCc3ccccc3	2.54	1.00	1.77
o1c2ccc(-c3ccccc3)cc2nc1NCCn4cncc4	0.39	3.16	1.77
O=C(N1CCN(CCC1)c2ccccc2)c3cc(on3)NCCCn4cncc4	0.39	3.16	1.78
O=C(NCCCn1cncc1)c2cnc(s2)-c3ccc(-c4ccccc4)cc3	0.40	3.16	1.78
O=C(NCCCn1cncc1)CCCc2nc(no2)-c3ccccc3	2.86	0.79	1.82
n1(CCCNc2cc(NCCCc3ccccc3)ncn2)cncc1	1.67	2.00	1.83
n1(Cc2cnc(nc2)-c3ccc(-c4ccccc4)cc3)cncc1	0.52	3.16	1.84
s1c(nnc1-c2ccccc2)/N=C/c3ccc(-n4ccnc4)cc3	0.55	3.16	1.85
s1c2cc(Nn3ccnc3)ccc2nc1SCc4ccccc4	0.58	3.16	1.87
O([C@@H]1COc2nc(cn2C1)-c3ccccc3)CC#Cn4ccnc4	0.58	3.16	1.87
O=C(N1CCC(CC1)COC(=O)NCCCn2cncc2)c3ccccc3	0.62	3.16	1.89
O=C(NCc1cnc(Sc2ccccc2)n1C)NCCCn3cncc3	1.33	2.51	1.92
O=C(NC1CCC(CC1)C(=O)NCCCn2cncc2)Cc3ccccc3	0.74	3.16	1.95
O=C(NCCCn1cncc1)C/C(=N/NC(=O)Cc2ccccc2)C	1.41	2.51	1.96
s1c2cc(On3ccnc3)ccc2nc1SCc4ccccc4	0.77	3.16	1.96

o1c(NCCCn2cncc2)nnc1C[C@@H]3CC[C@H](Nc4ccccc4)C3	0.78	3.16	1.97
O=C(NCCCn1cncc1)NCc2cccc(c2)C(=O)c3ccccc3	0.83	3.16	1.99
o1c2ccc(-c3ccccc3)cc2nc1N4CCC(n5cncc5)CC4	0.03	3.98	2.00
Fc1cc(-c2nc3cc(-c4ccccc4)ccc3[nH]2)ccc1-n5ccnc5	0.05	3.98	2.02
O=C(Nc1cc(on1)-n2ccnc2)c3cncc(c3)-c4ccccc4	0.05	3.98	2.02
O=C(NCCCn1cncc1)C2CCC(CC2)CNC(=O)c3ccccc3	0.87	3.16	2.02
[NH+]1(CCC[C@H](n2cncc2)CC1)Cc3cccc(-c4ccccc4)c3	0.07	3.98	2.02
N1(CCCN(CC1)c2ccccc2)c3ccc(Nn4cncc4)cc3	0.08	3.98	2.03
N1(n2ccnc2)CCC(n3c4ccc(cc4nn3)-c5ccccc5)CC1	0.14	3.98	2.06
Clc1c(-c2ccccc2)ccc(CC3CCN(n4ccnc4)CC3)c1	0.15	3.98	2.06
O=C(NCCCn1cncc1)c2cc3cc(NC(=O)c4ccccc4)ccc3o2	0.15	3.98	2.07
O=C(N/N=C/c1ccc(s1)-c2ccccc2)Cn3cncc3	1.62	2.51	2.07
N1(n2ccnc2)CCN(CC1)c3ccc4c([nH]c(n4)-c5ccccc5)n3	0.16	3.98	2.07
N1(CCC(CC1)c2ccccc2)c3ccc(Nn4ccnc4)nn3	0.16	3.98	2.07
O=C(Nn1ccnc1)c2ccc(-n3cc(-c4ccccc4)cn3)cc2	0.17	3.98	2.08
n1(-c2ccc(cc2)C#Cc3cn(nn3)-c4ccccc4)ccnc1	0.18	3.98	2.08
O=C(NCCC(=O)NCCCn1cncc1)[C@@H]2C[C@@H]2c3ccccc3	3.38	0.79	2.08
O=C(N[C@H](c1nc2ccc(-c3ccccc3)cc2s1)C)NCCCn4cncc4	0.19	3.98	2.09
O1Cc2c(NCCCn3cncc3)nnn2C[C@@H]1c4ccc(-c5ccccc5)cc4	0.22	3.98	2.10
O=C(Oc1ccccc1)/C=C/COC(=O)NCCCn2cncc2	3.42	0.79	2.10
O=C(Oc1ccc(Nn2ccnc2)cc1)/C=C/c3ccccc3	0.26	3.98	2.12
n1(CCC#Cc2ccc(-c3ccccc3)cc2)cncc1	0.28	3.98	2.13
S(Cc1ccccc1)Cc2nc3cc(-n4ccnc4)ccc3[nH]2	1.80	2.51	2.16
O=C(NCCCn1cncc1)c2c(n(nn2)-c3ccc(-c4ccccc4)cc3)N	0.34	3.98	2.16
O=C(Nn1ccnc1)c2cn(nn2)-c3ccc(-c4ccccc4)cc3	0.35	3.98	2.17
O=C(N1CCC2(CC1)CO[C@H](C2)Cc3ccccc3)NCCCn4cncc4	0.39	3.98	2.18
O=C1/C(CC[C@@H]1Cn2cncc2)=C/c3ccc(-c4ccccc4)cc3	0.40	3.98	2.19
O=C(N1CCC2(CC[NH+](C2)Cc3ccccc3)CC1)NCCCn4cncc4	0.49	3.98	2.24
O=C(NCCCn1cncc1)NC(=O)/C=C/c2ccc(-c3ccccc3)cc2	0.50	3.98	2.24
O=C(N1CC[C@H](OCCc2ccccc2)C1)NCCCn3cncc3	1.99	2.51	2.25
s1c(nnc1NCCCn2cncc2)-c3cccc(Oc4ccccc4)c3	0.54	3.98	2.26
O=S(=O)(c1cc2c(s1)CCN(C2)C(=O)NCCCn3cncc3)c4ccccc4	0.56	3.98	2.27
O=C(N1CCC[C@H](C1)CNC(=O)c2ccccc2)NCCCn3cncc3	0.59	3.98	2.29
O=S(=O)(Nn1cncc1)c2ccc(s2)C#Cc3ccccc3	0.60	3.98	2.29
O=C(NCCCn1cncc1)CCCc2ccc(s2)-c3ccccc3	2.59	2.00	2.29
O=C(Oc1ccccc1)/C=C/c2ccc(Cn3cncc3)cc2	0.64	3.98	2.31
O=C(N1CCc2c(sc(n2)C(=O)NCCCn3cncc3)CC1)c4ccccc4	0.83	3.98	2.40
o1c(nc(n1)-c2cc(on2)NCCCn3cncc3)CNc4ccccc4	0.83	3.98	2.41
O=C(NCC[NH+]1CCC[C@@](C1)(c2ccccc2)C)NCCCn3cncc3	0.87	3.98	2.43
[NH+]1(CCN(CC1)c2ccccc2)CCC#Cn3ccnc3	0.95	3.98	2.47
O=C(Nn1ccnc1)C(=O)Nc2nnc(s2)Cc3ccccc3	0.95	3.98	2.47
O=S(=O)(/N=C(/N/N=C(\C)C#Cn1ccnc1)N)c2ccccc2	0.97	3.98	2.48
S(c1ccccc1)c2ccc(N3CCC(n4cncc4)CC3)nn2	1.03	3.98	2.51
n12ccc(-n3ccnc3)nc1cc(n2)-c4ccc(-c5ccccc5)cc4	0.06	5.01	2.53
O=C(Nc1nc(-n2ccnc2)ns1)c3ccc(-c4ccccc4)cc3	0.13	5.01	2.57
O=C(Nc1ccc(-c2ccccc2)cc1)c3cc(-n4ccnc4)n[nH]3	0.14	5.01	2.58
O=C(Nc1cc(-n2ccnc2)n[nH]1)c3ccc(-c4ccccc4)cc3	0.14	5.01	2.58

O=C(Nc1nc2c(s1)C[C@H](CC2)c3ccccc3)C(=O)n4ccnc4	0.14	5.01	2.58
O=C1C(=COc2cc(On3ccnc3)ccc12)/C=C/c4ccccc4	0.16	5.01	2.59
O=C(NCCCn1cncc1)NCC#Cc2ccc(-c3ccccc3)cc2	0.16	5.01	2.59
Fc1cc(-c2cc(no2)C(=O)NCCCn3cncc3)ccc1-c4ccccc4	0.17	5.01	2.59
[NH+]1(C[C@H]2C([C@H](N2)C1)c3ccc(-c4ccccc4)cc3)Cn5cncc5	0.19	5.01	2.60
O=C(Nc1ccccc1)C#Cc2ccc(-n3ccnc3)cc2	0.20	5.01	2.61
O=C(NCCCn1cncc1)C(=O)Nc2cccc(Sc3ccccc3)c2	1.24	3.98	2.61
O=C(c1ccc(/C=C/C(=O)NCCCn2cncc2)cc1)c3ccccc3	0.21	5.01	2.61
N1(n2ccnc2)CCC(Nc3ccc(-c4ccccc4)cc3)CC1	0.23	5.01	2.62
N(C1CCC(n2cncc2)CC1)c3ccc(-c4ccccc4)cc3	0.24	5.01	2.62
O=C(c1ccc(C(=O)NC(=O)NCCCn2cncc2)cc1)c3ccccc3	0.24	5.01	2.63
O=C(NCCCn1cncc1)c2nc3ccc(Nc4ccccc4)cc3s2	0.25	5.01	2.63
O=C(Nn1ccnc1)c2cnc([nH]2)-c3ccc(-c4ccccc4)cc3	0.26	5.01	2.64
o1c(ccc1-c2ccc(-c3ccccc3)cc2)/C=C/n4ccnc4	0.27	5.01	2.64
O=C(Oc1cccc(-n2ccc(c2)-c3ccccc3)c1)NCCCn4cncc4	0.27	5.01	2.64
O=C(Nc1ccccc1)c2cc(on2)-c3ccc(-n4ccnc4)cc3	0.29	5.01	2.65
O=C(NCCCn1cncc1)/C=C/Nc2cccc(-c3ccccc3)c2	0.29	5.01	2.65
C/C(=N\c1ccccc1)/C=N/Nc2ccc(-n3ccnc3)cc2	0.30	5.01	2.66
C/C(=N\n1ccnc1)/C=N/Nc2ccc(-c3ccccc3)cc2	0.31	5.01	2.66
O=C(NCCCn1cncc1)NC/C=C/c2ccc(-c3ccccc3)cc2	0.42	5.01	2.72
O=C(NCCCn1cncc1)c2c(nc(s2)N3C=NN(C3=O)c4ccccc4)C	0.46	5.01	2.74
S(Cc1ccccc1)c2ccc(CCn3cncc3)cc2	2.32	3.16	2.74
O=C(NCCCn1cncc1)CSc2nnc(o2)Cc3ccccc3	4.50	1.00	2.75
[nH+]1cc(ccc1N2CCC(Cn3cncc3)CC2)-c4ccccc4	0.50	5.01	2.75
O=C(Oc1ccccc1)c2cccc(-n3c(NCCCn4cncc4)nnn3)c2	0.50	5.01	2.76
O=C(N/N=C/c1ccccc1)C[NH+]2CCN(n3ccnc3)CC2	2.40	3.16	2.78
O=C(NCCc1ccc(s1)-c2ccccc2)NCCCn3cncc3	1.61	3.98	2.80
O=C(On1ccnc1)c2ccc(N/N=N/c3ccccc3)cc2	0.64	5.01	2.83
O=C(Nn1ccnc1)c2nnc(s2)[C@@H]3CCCN(C3)c4ccccc4	0.70	5.01	2.86
s1c(CO/N=C/c2ccccc2)cnc1NCCCn3cncc3	3.24	2.51	2.87
O=C(NCCCn1cncc1)NCc2ccc(Cc3ccccc3)cc2	0.74	5.01	2.88
O=C(Oc1ccccc1)/C=C/c2ccc(Sn3ccnc3)cc2	0.75	5.01	2.88
O=C(NCCCn1cncc1)c2cc3cc(ccc3n2C)C	0.80	5.01	2.91
O=C(NCCCn1cncc1)NCc2cccc(Sc3ccccc3)c2	0.80	5.01	2.91
O=C(NCCCn1cncc1)NCc2cccc(N(c3ccccc3)C)c2	0.96	5.01	2.98
O=C(NCC(=O)NCCCn1cncc1)C2CCN(CC2)c3ccccc3	1.00	5.01	3.01
O=C(Nn1ccnc1)CNC(=O)c2ccc(s2)-c3ccccc3	1.03	5.01	3.02
O=C(NCCCn1cncc1)c2ccc(s2)CNC(=O)c3ccccc3	2.07	3.98	3.02
[NH+]1(C2CCN(CC2)c3ccccc3)CCC(Nn4ccnc4)CC1	1.08	5.01	3.04
S(CCNc1ccccc1)c2nnc(s2)NCCCn3cncc3	4.57	1.58	3.08
O=C(NCCc1ccccc1)c2cnc(s2)NCCCn3cncc3	3.04	3.16	3.10
O=C(NCCCn1cncc1)c2ccc-3c(Cc4cc(-c5ccccc5)ccc43)c2	0.05	6.31	3.18
O=C(Nc1ccccc1)C[NH+]2CCC[C@H](C2)C(=O)NCCCn3cncc3	1.35	5.01	3.18
O=C(NCCCn1cncc1)NCc2nnc(s2)Sc3ccccc3	1.36	5.01	3.19
[NH+]1(CCN(n2ccnc2)CCC1)Cc3ccc(-c4ccccc4)cc3	0.08	6.31	3.20
o1c2ccc(-n3ccnc3)cc2nc1-c4cnc(-c5ccccc5)cc4	0.09	6.31	3.20
s1c2cc(-c3ccccc3)cnc2nc1N4CCC(n5cncc5)CC4	0.13	6.31	3.22

O=C(NC1CCC2(CC1)CCN(n3ccnc3)CC2)c4ccccc4	0.14	6.31	3.22
O=C(N[C@@H]1CCN(n2ccnc2)C1)c3ccc(-c4ccccc4)cc3	0.16	6.31	3.24
O(C1CCN(n2ccnc2)CC1)c3ccc(-c4ccccc4)cc3	0.17	6.31	3.24
O=C(On1ccnc1)c2ccc(o2)-c3ccc(-c4ccccc4)cc3	0.19	6.31	3.25
O=C(Nc1ccc2cc(sc2c1)-c3ccccc3)Cn4cncc4	0.20	6.31	3.25
O=C(NCCCn1cncc1)n2cc(nn2)-c3ccc(-c4ccccc4)cc3	0.20	6.31	3.26
O=C(NCCCn1cncc1)N/N=C\2[C@H](OC3(C2)CCN(CC3)c4ccccc4)C	0.20	6.31	3.26
O=C(NCCCn1cncc1)c2nnc(o2)-c3cccc(-c4ccccc4)c3	0.21	6.31	3.26
O=C(NCCCn1cncc1)Nc2cccc(c2)C#Cc3ccccc3	0.21	6.31	3.26
O=C(NCCCn1cncc1)c2nc(no2)-c3ccc(-c4ccccc4)cc3	0.22	6.31	3.27
O=C(Nc1ccc(-c2ccccc2)cc1)[C@@H]3C[C@H](n4cncc4)C[NH2+]3	0.23	6.31	3.27
O=C(NCCCn1cncc1)[C@@H]2CC(=NO2)c3ccc(-c4ccccc4)cc3	0.27	6.31	3.29
O=C(Nn1ccnc1)c2cnn(n2)-c3ccc(-c4ccccc4)cc3	0.28	6.31	3.30
O=C(Nn1ccnc1)c2cnn(-c3ccc(-c4ccccc4)cc3)c2	0.28	6.31	3.30
Cc1c(NCCCn2cncc2)c(on1)/C=C/c3ccc(s3)-c4ccccc4	0.31	6.31	3.31
O=C(NCCCn1cncc1)C2CCN(CC2)c3ccc(c[nH+]3)-c4ccccc4	0.33	6.31	3.32
O=C(NCCCn1cncc1)c2ncc(o2)-c3ccc(-c4ccccc4)cn3	0.33	6.31	3.32
O=C(NC[C@H](C1CCN(CC1)c2ccccc2)C)NCCCn3cncc3	1.64	5.01	3.33
O=C(OC1CCCC(OC(=O)c2ccccc2)CCC1)NCCCn3cncc3	0.37	6.31	3.34
O=C(NCCCn1cncc1)C/C=C/c2ccc(-c3ccccc3)cc2	0.45	6.31	3.38
O=C(NC1CCN(n2ccnc2)CC1)c3cc(on3)-c4ccccc4	0.46	6.31	3.39
[NH+]1(C2CCN(CC2)c3ccccc3)CCC(CC1)Cn4cncc4	0.46	6.31	3.39
O=C(NC1CCC(CC1)Cn2cncc2)/C=C/c3ccccc3	0.48	6.31	3.40
O=C(NC1CCC(Oc2ccccc2)CC1)/C=C/n3ccnc3	0.56	6.31	3.44
n1(/C=C/CC/C=C/C=C/c2ccccc2)ccnc1	2.89	3.98	3.44
O=C(NCCCn1cncc1)c2csc(n2)-c3ccc(s3)-c4ccccc4	0.59	6.31	3.45
O=C(NCC1CCN(n2ccnc2)CC1)/C=C/c3ccccc3	0.80	6.31	3.56
N/C(Nn1ccnc1)=N\N=C\c2ccc(-c3ccccc3)cc2	0.83	6.31	3.57
S(CC#CCc1ccccc1)c2nnc(o2)-n3ccnc3	0.87	6.31	3.59
S(CCOc1ccccc1)c2nnc(o2)NCCCn3cncc3	4.70	2.51	3.61
o1c(nc(NCCCn2cncc2)n1)[C@@H]3CCC[NH+](C3)Cc4ccccc4	0.96	6.31	3.63
O=C(N1CCC(CC1)CNC(=O)c2ccccc2)NCCCn3cncc3	1.00	6.31	3.66
O=C(NCCCn1cncc1)c2ccnc(C(=O)NCc3ccccc3)c2	1.11	6.31	3.71
O=C(Nc1ccccc1)Cn2cnc(n2)C(=O)NCCCn3cncc3	2.49	5.01	3.75
O=C(N1CC(C1)Cc2cnn(c2)-c3ccccc3)NCCCn4cncc4	1.28	6.31	3.80
S(Sc1ccc(-c2ccccc2)cc1)CCn3cncc3	1.35	6.31	3.83
O=C(NCCCn1cncc1)NCc2ccc(Oc3ccccc3)cc2	1.39	6.31	3.85
O=C(OCC1CCN(CC1)c2ccccc2)/C=C/n3ccnc3	1.50	6.31	3.91
s1c(-c2nc3cc(-n4ccnc4)ccc3[nH]2)ccc1-c5ccccc5	0.06	7.94	4.00
C[n+]1c(cn2ccc(-n3ccnc3)cc21)-c4ccc(-c5ccccc5)cc4	0.07	7.94	4.00
n1(-c2cncc(c2)C#Cc3cnc(-c4ccccc4)cc3)ccnc1	0.07	7.94	4.01
[NH+]1(CCCN(n2cncc2)CC1)Cc3cccc(-c4ccccc4)c3	0.08	7.94	4.01
O=C(Oc1ccccc1)c2ccc(o2)COC(=O)NCCCn3cncc3	3.02	5.01	4.02
N1(n2ccnc2)CCC(CC1)c3nc4ccc(cc4[nH]3)-c5ccccc5	0.10	7.94	4.02
N1(n2ccnc2)CCN(CC1)c3ccc(Nc4ccccc4)cc3	0.12	7.94	4.03
O=C(Nc1cc(on1)-n2ccnc2)c3ccc(-c4ccccc4)cc3	0.12	7.94	4.03
O=C(N1CCC(CC1)c2cc([nH]n2)-c3ccccc3)NCCCn4cncc4	0.13	7.94	4.04

O=C(NCCCn1cncc1)/C=C/c2ccc3cc(-c4ccccc4)ccc3c2	0.13	7.94	4.04
[NH+]1(Cc2cccc(-n3ccnc3)c2)CCN(CCC1)c4ccccc4	0.15	7.94	4.05
O=C(c1ccccc1)c2cnc(N3CCC(n4cncc4)CC3)cc2	0.17	7.94	4.06
Clc1cc(-c2ccc(o2)/C=N/c3ccccc3)ccc1-n4ccnc4	0.17	7.94	4.06
O=C(On1ccnc1)c2cc(on2)-c3ccc(-c4ccccc4)cc3	0.18	7.94	4.06
O1C[C@H](n2ccnc2)CC31CCN(CC3)c4ccc(cc4)-c5ccccc5	0.19	7.94	4.06
Clc1c(noc1-c2ccc(-c3ccccc3)cc2)C(=O)NCCCn4cncc4	0.20	7.94	4.07
O=C(Nn1ccnc1)c2ccc(N3CC[C@H](C3)c4ccccc4)cc2	0.21	7.94	4.07
O=C(NCCCn1cncc1)C2CCN(CC2)c3ccc(cc3)-c4ccccc4	0.21	7.94	4.07
O=C(NCCCn1cncc1)c2cc(N3CCC[C@H](C3)c4ccccc4)ncn2	0.23	7.94	4.09
N1(CCN(CCC1)c2ccc3nnc(n3n2)NCCCn4cncc4)c5ccccc5	0.25	7.94	4.10
O=C(NCCCn1cncc1)c2nc3cc(C(=O)c4ccccc4)ccc3o2	0.28	7.94	4.11
N(C1CCC(CC1)c2ccccc2)c3ccc(-n4ccnc4)cc3	0.30	7.94	4.12
O=C(Nc1nc(C(=O)NCCCn2cncc2)cs1)Cc3ccccc3	1.95	6.31	4.13
O=C(c1ccccc1)/C=C/c2ccc(cc2)/C=N/n3ccnc3	0.32	7.94	4.13
s1c(-c2csc(n2)-c3ccccc3)ccc1CCn4cncc4	0.37	7.94	4.15
O=C(Oc1ccc(Cn2cncc2)cc1)/C=C/c3ccccc3	0.37	7.94	4.16
[NH+]1(CCC(CC1)c2ccc(-c3ccccc3)cc2)Cn4cncc4	0.38	7.94	4.16
S=C(SCCc1nnc(o1)NCCCn2cncc2)c3ccccc3	2.06	6.31	4.18
[NH+]1(C2CCC(Nn3ccnc3)CC2)CCN(CC1)c4ccccc4	0.44	7.94	4.19
Cc1c[n+](CCCC#Cc2ccccc2)ccc1-n3ccnc3	0.46	7.94	4.20
O=C(OCC#CCOc1ccccc1)NCCCn2cncc2	2.14	6.31	4.22
[NH+]1(CCC(C2CCN(CC2)c3ccccc3)CC1)Cn4cncc4	0.51	7.94	4.22
S(SC/C=C/n1ccnc1)C/C=C/c2ccccc2	5.94	2.51	4.23
O=C(NCCCn1cncc1)c2cccc(c2)C(=O)NCc3ccccc3	0.54	7.94	4.24
N1(CCC(Cn2cncc2)CC1)c3ncc(-c4ccccc4)cn3	0.55	7.94	4.24
O(C1CCN(CC1)c2ccccc2)c3ccc(-n4ccnc4)cc3	0.56	7.94	4.25
[nH+]1cc(Nn2cncc2)ccc1N3CCN(CC3)c4ccccc4	0.57	7.94	4.26
[nH]1c2ccc(-n3ccnc3)cc2nc1CCCc4ccccc4	0.60	7.94	4.27
O=C(NCCCn1cncc1)c2cc(no2)[C@@H]3CCCN(C3)c4ccccc4	0.62	7.94	4.28
O=S(=O)(N1CCC(CC1)C(=O)NCCCn2cncc2)Cc3ccccc3	0.65	7.94	4.30
o1c(nc(n1)CCn2cncc2)[C@@H]3CCN(C3)c4ccccc4	0.76	7.94	4.35
O=C(C1CCC(CC1)CNC(=O)NCCCn2cncc2)c3ccccc3	0.79	7.94	4.37
O=C(CCc1nn2c(s1)nnc2NCCCn3cncc3)c4ccccc4	0.88	7.94	4.41
O=C(OCc1ccc(Oc2ccccc2)cc1)NCCCn3cncc3	0.89	7.94	4.42
O=C(Nc1ccccc1)C(=O)Nc2nnc(s2)Cn3cncc3	0.99	7.94	4.47
S(Cc1ncc(o1)-c2ccccc2)c3cnc(s3)-n4cncc4	1.02	7.94	4.48
O1[C@H](CCCCc2ccccc2)CC(NCCCn3cncc3)=N1	5.02	3.98	4.50
O=C(NCCCn1cncc1)NCc2ccc(s2)Cc3ccccc3	1.11	7.94	4.53
s1c(NCCCn2cncc2)nnc1CCSCc3ccccc3	4.10	5.01	4.56
O=C(N1CC(n2cc(nn2)Cc3ccccc3)C1)NCCCn4cncc4	1.42	7.94	4.68
O=C(NCCCn1cncc1)CCc2nc(no2)Cc3ccccc3	1.92	7.94	4.93
O=C(Oc1ccccc1)CSc2nnc(o2)NCCCn3cncc3	8.66	1.26	4.96
Cn1c(SCCCc2ccccc2)nnc1NCCCn3cncc3	2.04	7.94	4.99
N1(CCC(CC1)c2nc3ccc(-n4ccnc4)cn3n2)c5ccccc5	0.05	10.00	5.03
[nH]1c2ccc(-c3ccccc3)cc2nc1-c4cnc(-n5ccnc5)cc4	0.07	10.00	5.03
Fc1cnc(NCCCn2cncc2)nc1Nc3ccc(-c4ccccc4)cc3	0.08	10.00	5.04

O=C1C=C(Nc2ccc(Nc3ccccc3)cc12)C(=O)NCCCn4cncc4	0.09	10.00	5.05
N1(CCC(CC1)c2cc3cc(-n4cncc4)ccc3[nH]2)c5ccccc5	0.10	10.00	5.05
O=C(NCCCn1cncc1)CSc2nnc(s2)Nc3ccccc3	2.16	7.94	5.05
O=C(c1cc2cc(-n3ccnc3)ccc2o1)/C=C/c4ccccc4	0.12	10.00	5.06
O=C(NCCCn1cncc1)c2ccc(o2)-c3cccc(-c4ccccc4)c3	0.14	10.00	5.07
N1(n2ccnc2)CCC(n3c4cnc(cc4nn3)-c5ccccc5)CC1	0.16	10.00	5.08
O=C(NCCCn1cncc1)C(=O)/C=C/c2cccc(-c3ccccc3)c2	0.20	10.00	5.10
[NH+]1(CC2CCC(CC2)c3ccccc3)CCC(n4cncc4)CC1	0.21	10.00	5.10
O1C[C@H](CC21CCN(CC2)c3ccccc3)c4cnn(-n5ccnc5)c4	0.23	10.00	5.12
O=C(Nc1ccc(-c2ccccc2)cc1)[C@@H]3CCN(n4ccnc4)C3	0.24	10.00	5.12
S(n1ccnc1)C2COC(OC2)/C=C/C=C/c3ccccc3	2.30	7.94	5.12
o1c(ncc1-c2ccc(-n3ccnc3)cc2)/C=N/c4ccccc4	0.26	10.00	5.13
N1(CCC(Cn2cncc2)CC1)c3ccc(nn3)-c4ccccc4	0.26	10.00	5.13
O=C(NCCCn1cncc1)c2cnn(c2)-c3nc(-c4ccccc4)cs3	0.27	10.00	5.13
O=C(N1CCN(CC1)c2ncc(-c3ccccc3)cn2)NCCCn4cncc4	0.29	10.00	5.14
s1c(nnc1NCn2cncc2)-c3ccc(o3)-c4ccccc4	0.33	10.00	5.17
C[C@@H]1C[NH+](CCN1c2ccc(-c3ccccc3)cc2)Cn4cncc4	0.34	10.00	5.17
O=C(N1CC[C@H](CCC1)CCc2ccccc2)NCCCn3cncc3	0.37	10.00	5.18
s1c2cc(Nc3ccccc3)ccc2nc1SCn4cncc4	0.37	10.00	5.18
O=C(c1ccc(-n2ccnc2)cc1)/C=C/C=C/c3ccccc3	0.42	10.00	5.21
O=C(NC1CCN(n2ccnc2)CC1)c3cc(n[nH]3)-c4ccccc4	0.42	10.00	5.21
O=C(Nc1nc(ns1)-c2ccccc2)c3ccc(-n4ccnc4)cc3	0.47	10.00	5.24
[nH]1c2ccc(-n3ccnc3)cc2nc1CCNc4ccccc4	0.51	10.00	5.25
o1c(ccc1-c2ccccc2)/C=C/C=N/Nn3cncc3	0.54	10.00	5.27
s1c(nnc1NCCCn2cncc2)/N=C/c3ccc(-c4ccccc4)cc3	0.57	10.00	5.28
[n+]1(CCCC#Cn2ccnc2)ccc(-c3ccccc3)cc1	0.57	10.00	5.28
O=C(N1CC[C@@]2(C[C@H](Nc3ccccc3)CCO2)C1)NCCCn4cncc4	0.70	10.00	5.35
O=S(=O)(C1CCN(CC1)C(=O)NCCCn2cncc2)Cc3ccccc3	0.70	10.00	5.35
O=C(OC1CCC(N(c2ccccc2)C)CC1)C#Cn3ccnc3	0.70	10.00	5.35
O=C(Nc1nc(-c2ccccc2)cs1)c3cnc(-n4ccnc4)cn3	0.72	10.00	5.36
O=S(=O)(N1CC=C(CC1)c2ccccc2)CC(=O)NCCCn3cncc3	0.73	10.00	5.36
O=C(NCCCn1cncc1)c2cnc(s2)-c3cnn(c3)-c4ccccc4	0.73	10.00	5.36
O=C(NCCCn1cncc1)Nc2cccc(CCc3ccccc3)c2	0.76	10.00	5.38
O=C(On1ccnc1)CNC(=O)c2ccc(s2)-c3ccccc3	0.88	10.00	5.44
O=C(NNC(=O)c1ccccc1)CCC(=O)NCCCn2cncc2	4.58	6.31	5.45
S(Cn1cncc1)c2ccc(CCc3ccccc3)cc2	3.01	7.94	5.48
S(Cc1noc(n1)-c2ccccc2)c3nnc(NCCCn4cncc4)[nH]3	0.98	10.00	5.49
O=C(NCCCn1cncc1)CC[NH+]2CC=C(CC2)c3ccccc3	1.08	10.00	5.54
N1(CCC(CC1)Cn2cc(NCCCn3cncc3)cn2)c4ccccc4	1.09	10.00	5.55
O=C(NCCCn1cncc1)N/N=C\c2cccc(Oc3ccccc3)c2	1.13	10.00	5.57
O=C(C(=O)NCCCn1cncc1)/C=C/c2ccc(s2)-c3ccccc3	1.23	10.00	5.61
O=C(NCCCn1cncc1)C(=O)Nc2cccc(Oc3ccccc3)c2	1.34	10.00	5.67
O=C(NCCCn1cncc1)c2nnc(s2)CSCc3ccccc3	5.04	6.31	5.67
[NH+]1(CCC(n2cncc2)CC1)CCCCc3ccccc3	1.50	10.00	5.75
O=C(NCCCn1cncc1)NCc2csc(n2)Nc3ccccc3	1.87	10.00	5.94
N1(CCC(CC1)Cn2cc(NCCCn3cncc3)nn2)c4ccccc4	1.96	10.00	5.98
O=C(NCCCn1cncc1)NCC[C@@H]2CN(CCC2)c3ccccc3	2.10	10.00	6.05

O=C(Oc1ccccc1)CCCOC(=O)NCCCn2cncc2	10.62	1.58	6.10
n1(/C=C/C=C/CC/C=C/c2ccccc2)ccnc1	4.28	7.94	6.11
O=C(OCCCCCc1ccccc1)NCCCn2cncc2	4.29	7.94	6.12
O=C(Nc1cnc(Oc2ccccc2)cc1)C(=O)NCCCn3cncc3	2.24	10.00	6.12
n1(-c2cccc(c2)C#Cc3cnc(nc3)-c4ccccc4)ccnc1	0.02	12.59	6.31
O=C(NCc1nnc(s1)NCCCn2cncc2)Cc3ccccc3	2.62	10.00	6.31
O=C(NCCCn1cncc1)NCCCCCc2ccccc2	7.63	5.01	6.32
Cn1c2ccc(cc2nc1-c3ccc(-n4ccnc4)cc3)-c5ccccc5	0.07	12.59	6.33
N1(n2ccnc2)CCC(CC1)Cc3ccc(-c4ccccc4)cc3	0.11	12.59	6.35
Fc1cc(-c2ccccc2)ccc1OC3CCN(n4ccnc4)CC3	0.11	12.59	6.35
s1c(nn2c1nnc2NCCCn3cncc3)-c4ccc(-c5ccccc5)cc4	0.13	12.59	6.36
Fc1c(-c2ccccc2)ccc(C[NH+]3CCN(n4ccnc4)CC3)c1	0.14	12.59	6.37
Nc1nc(N2CCN(n3ccnc3)CC2)nc4nc(nn14)-c5ccccc5	0.15	12.59	6.37
N1(CCC[C@H](C1)c2ccccc2)c3ccc4nnc(n4n3)NCCCn5cncc5	0.17	12.59	6.38
O=C(NCCCn1cncc1)c2cnn(-c3cccc(-c4ccccc4)c3)c2	0.17	12.59	6.38
O=C(Nc1ccc(-n2ccnc2)cc1)[C@@H]3C[C@H](C[NH2+]3)c4ccccc4	0.18	12.59	6.38
n1(C2CCC(CC3CCC(CC3)c4ccccc4)CC2)cncc1	0.21	12.59	6.40
Clc1cc(C=2C[C@H](ON2)C(=O)NCCCn3cncc3)ccc1-c4ccccc4	0.21	12.59	6.40
O(n1ccnc1)C2CCN(CC2)c3ccc(nn3)-c4ccccc4	0.22	12.59	6.41
O=C(NCCCn1cncc1)c2cccc(c2)C(=O)/C=C/c3ccccc3	0.24	12.59	6.42
O=C(NCCCn1cncc1)c2cc(on2)-c3ccc(-c4ccccc4)cc3	0.25	12.59	6.42
FC1(CN(C1)C(=O)NCCCn2cncc2)c3ccc(cc3)-c4ccccc4	0.25	12.59	6.42
O=C(Nc1ccccc1)c2ccc(N3CC[C@H](n4cncc4)C3)cc2	0.26	12.59	6.42
O=C([C@@H]1CCc2c(C1)cc(s2)C(=O)NCCCn3cncc3)c4ccccc4	0.27	12.59	6.43
[NH+]1(CC2CCC(n3cncc3)CC2)CCC(CC1)c4ccccc4	0.29	12.59	6.44
S(Cn1cncc1)c2nnc3c4cc(nn4ccn23)-c5ccccc5	0.29	12.59	6.44
O=C(NCCCn1cncc1)NCc2csc(n2)N(c3ccccc3)C	2.89	10.00	6.44
O=C(Nn1ccnc1)c2nnn(n2)-c3ccc(-c4ccccc4)cc3	0.31	12.59	6.45
O=C(Nc1ccccc1)c2nnc(s2)[C@@H]3CCCN(n4ccnc4)C3	0.32	12.59	6.46
O=C(OCn1cncc1)/C=C/c2ccc(-c3ccccc3)cc2	0.33	12.59	6.46
N1(n2ccnc2)CCC3(CC(C3)CCCc4ccccc4)CC1	0.36	12.59	6.47
[NH+]1(CC2CCN(n3ccnc3)CC2)CCC(CC1)c4ccccc4	0.36	12.59	6.48
O=C(Nc1cnn(-n2ccnc2)c1)c3cc(on3)-c4ccccc4	0.37	12.59	6.48
o1c2ccc(-n3ccnc3)cc2nc1CCCc4ccccc4	0.47	12.59	6.53
O=C(NCCCn1cncc1)c2cc(no2)-c3cnn(-c4ccccc4)c3	0.47	12.59	6.53
O(CCc1ccccc1)c2ccc3c(nc(o3)-n4ccnc4)c2	0.47	12.59	6.53
O=C(NCCCn1cncc1)c2csc(n2)[C@@H]3C[C@H](CCO3)c4ccccc4	0.49	12.59	6.54
O=C(OCc1ccccc1)/C=C/c2ccc(-n3ccnc3)cc2	0.50	12.59	6.54
[NH+]1(C2CCC(CC2)Cn3cncc3)CC=C(CC1)c4ccccc4	0.50	12.59	6.55
S(Cc1ccccc1)c2nc3ccc(On4ccnc4)cc3[nH]2	0.51	12.59	6.55
O=C(NCCCn1cncc1)c2coc(n2)-c3coc(n3)-c4ccccc4	0.62	12.59	6.60
O=C(NCCCn1cncc1)NCc2nnc3C[C@H](CCn23)c4ccccc4	0.63	12.59	6.61
O=C(NCCCn1cncc1)/C=C/c2ccc3c(c2)ccn3-c4ccccc4	0.65	12.59	6.62
O=C(N[C@@H]1CC[NH+](C2CCN(n3ccnc3)CC2)C1)c4ccccc4	0.66	12.59	6.62
O=C(Nc1cccc(CNc2ccccc2)c1)NCCCn3cncc3	0.74	12.59	6.66
o1cc(nc1-n2ccnc2)C[NH2+][C@@H]3CCN(C3)c4ccccc4	0.76	12.59	6.67
O=C1N(n2ccnc2)C[C@H](O1)CNC(=O)/C=C/c3ccccc3	0.78	12.59	6.68

O=C(Nc1ccc(Nc2ccccc2)cc1)C(=O)NCCCn3cncc3	0.79	12.59	6.69
C/C(=C\c1ccccc1)/C=C/C=C(\C)/C=C/n2ccnc2	0.83	12.59	6.71
O=C(NCCCn1cncc1)N/N=C\c2ccc(Oc3ccccc3)cc2	0.86	12.59	6.73
O=C(NCCCn1cncc1)c2cnc(N3CCN(CC3)c4ccccc4)cc2	0.99	12.59	6.79
O(n1ccnc1)CC#CC#CCOc2ccccc2	3.62	10.00	6.81
s1c(C[NH+]2CC[C@H](C2)c3ccccc3)cnc1NCCCn4cncc4	1.20	12.59	6.89
O=C(OCCC1CCN(CC1)c2ccccc2)NCCCn3cncc3	1.22	12.59	6.90
O=C(Nc1cncc(C(=O)NCCCn2cncc2)c1)Cc3ccccc3	1.48	12.59	7.03
O=C(NC1CCC(Nc2ccccc2)CC1)C(=O)NCCCn3cncc3	1.53	12.59	7.06
O=C(NCCCn1cncc1)c2ccc(SCc3ccccc3)cc2	1.58	12.59	7.08
O=C(NCCCn1cncc1)c2nnc(s2)CNC(=O)c3ccccc3	1.70	12.59	7.14
S(CCOc1ccccc1)c2nnc(s2)NCCCn3cncc3	13.23	1.26	7.24
O=C(NCCCn1cncc1)c2ccc(s2)CCCc3ccccc3	1.91	12.59	7.25
o1c(nnc1NCCCn2cncc2)C[NH2+]CCc3ccccc3	4.59	10.00	7.29
O=C(NCCCn1cncc1)c2ccc(N3CCN(CC3)c4ccccc4)cn2	2.13	12.59	7.36
s1c(NCCCc2ccccc2)nnc1NCCCn3cncc3	2.14	12.59	7.36
O=C(NCCCn1cncc1)C2CCC([NH2+]Cc3ccccc3)CC2	2.19	12.59	7.39
S(Cc1nnc(s1)SCn2cncc2)c3ccccc3	8.59	6.31	7.45
O=C(NCCCn1cncc1)CC/C=C/CCc2ccccc2	4.97	10.00	7.48
O=C(NCCCn1cncc1)CNC(=O)c2cc(n(n2)C)-c3ccccc3	2.49	12.59	7.54
O=C(NC[C@H]([NH3+])c1ccccc1)/C=C/C(=O)NCCCn2cncc2	2.55	12.59	7.57
S(CCC[NH+]1CCN(n2ccnc2)CC1)c3ccccc3	2.60	12.59	7.60
O=C(NCCCn1cncc1)CCCn2cc(-c3ccccc3)cn2	2.68	12.59	7.64
O=C(NCCCn1cncc1)CSc2nc(Sc3ccccc3)ns2	9.05	6.31	7.68
n1(CCCCc2ccccc2)cnc(NCCCn3cncc3)c1	2.92	12.59	7.75
O=C(NCCn1cc(-c2ccccc2)cn1)NCCCn3cncc3	2.95	12.59	7.77
O=C(Nc1ncc(s1)-n2cncc2)c3cccc(-c4ccccc4)c3	0.03	15.85	7.94
s1cc(-n2ccnc2)nc1NN=C3CCC(CC3)c4ccccc4	0.06	15.85	7.96
o1c2ccc(-c3ccccc3)cc2nc1-c4cnc(-n5ccnc5)cc4	0.08	15.85	7.96
n1(nc2ccc(-n3ccnc3)cc2n1)-c4ccc(-c5ccccc5)cc4	0.08	15.85	7.96
s1c(ccc1-n2ccnc2)C#Cc3ccc(-c4ccccc4)cc3	0.08	15.85	7.97
O1c2ccc(On3ccnc3)cc2CCC41CCN(CC4)c5ccccc5	0.09	15.85	7.97
Fc1cc(-c2nc3cc(-n4ccnc4)ccc3[nH]2)ccc1-c5ccccc5	0.09	15.85	7.97
N1(CCC(CC1)c2nc3cc(-n4ccnc4)ccc3[nH]2)c5ccccc5	0.10	15.85	7.97
O=C(c1ccc(N2CCC(n3cncc3)CC2)cc1)c4ccccc4	0.10	15.85	7.97
N1(n2ccnc2)CCN(CC1)c3ccc4c([nH]c(n4)-c5ccccc5)c3	0.10	15.85	7.97
n1(-c2ccc(-c3nc4cc(-c5ccccc5)cnc4[nH]3)cc2)ccnc1	0.11	15.85	7.98
O(CCCn1cc(NCCCn2cncc2)cn1)c3ccccc3	10.95	5.01	7.98
Fc1cc2c(onc2C3CCN(n4ccnc4)CC3)cc1-c5ccccc5	0.13	15.85	7.99
O=C1c2c(CN1n3ccnc3)ccc(n2)-c4ccc(-c5ccccc5)cc4	0.13	15.85	7.99
O=C(Nc1nnc(o1)-n2ccnc2)c3ccc(-c4ccccc4)cc3	0.14	15.85	8.00
O=C(Nn1cncc1)c2cc(on2)-c3cccc(-c4ccccc4)c3	0.15	15.85	8.00
O=C(NCCCn1cncc1)c2cc(n(n2)C)-c3ccc(-c4ccccc4)cc3	0.16	15.85	8.01
o1c2cnc(cc2cc1C3CCN(n4ccnc4)CC3)-c5ccccc5	0.17	15.85	8.01
O=C1c2cc(nn2C=NN1n3ccnc3)-c4ccc(-c5ccccc5)cc4	0.17	15.85	8.01
C/C(=C\CC1CCC(CC1)c2ccccc2)/C=C/n3ccnc3	0.20	15.85	8.03
s1c2c(nc1N3CCN(n4ccnc4)CC3)CC[C@H](C2)c5ccccc5	0.22	15.85	8.03

[NH+]1(C2CCN(n3ccnc3)CC2)CCC(Nc4ccccc4)CC1	0.23	15.85	8.04
O=C(NCCCn1cncc1)c2ccc(s2)-c3ccc(-c4ccccc4)cc3	0.26	15.85	8.05
Clc1cc(CC2CCN(CC2)c3ccccc3)ccc1-n4ccnc4	0.27	15.85	8.06
O=C(N[C@@H]1CCN(n2ccnc2)C1)c3ccc(-c4ccccc4)cn3	0.28	15.85	8.06
n1(CCCNc2nccc(Nc3ccc(-c4ccccc4)cc3)n2)cncc1	0.28	15.85	8.07
Cc1cc(ccc1N2CC[NH+](Cn3cncc3)CC2)-c4ccccc4	0.29	15.85	8.07
[NH+]1(CC2CCC(CC2)c3ccccc3)CCN(n4ccnc4)CC1	0.30	15.85	8.07
O=C(NCCCn1cncc1)c2cnn(-c3ccc(-c4ccccc4)cc3)c2	0.30	15.85	8.08
O=C(NCCCn1cncc1)c2cnn(c2N)-c3ccc(-c4ccccc4)cc3	0.31	15.85	8.08
O=C(NCCCn1cncc1)c2c(oc(n2)C3CCN(CC3)c4ccccc4)C	0.33	15.85	8.09
O=C(NCCCn1cncc1)[C@@H]2COc3ccc(Oc4ccccc4)cc3C2	0.34	15.85	8.10
O=C(NCCCn1cncc1)c2cnc(N3C[C@@H]4[C@@H](C4c5ccccc5)C3)nc2	0.35	15.85	8.10
O=C(NCCCn1cncc1)[C@@H]2COc3cc(Oc4ccccc4)ccc3C2	0.37	15.85	8.11
O=C(OCc1cn2c(n1)ccc(c2)-c3ccccc3)NCCCn4cncc4	0.41	15.85	8.13
[NH+]1(CCN(CCC1)c2ccccc2)Cc3ccc(-n4ccnc4)cc3	0.45	15.85	8.15
O=C(NCCCn1cncc1)c2cn(nn2)-c3ccc(-c4ccccc4)cn3	0.46	15.85	8.16
O=C(/C=C/NCCCn1cncc1)/C=C/c2ccc(-c3ccccc3)cc2	0.47	15.85	8.16
O=C(NCCCn1cncc1)c2csc(n2)-c3ccc(o3)-c4ccccc4	0.47	15.85	8.16
O=C(OCn1cncc1)/C=C/c2ccc(o2)-c3ccccc3	0.49	15.85	8.17
O=C(NC1CC(C1)c2cc(ncn2)-c3ccccc3)NCCCn4cncc4	0.51	15.85	8.18
O=C(NCCCn1cncc1)c2nnc(s2)C3CCN(CC3)c4ccccc4	0.53	15.85	8.19
s1cc(nc1CCc2ccccc2)-c3nc(-n4ccnc4)cs3	0.55	15.85	8.20
O=C(NCCCn1cncc1)/C=C/C=C/c2ccc(-c3ccccc3)cc2	0.57	15.85	8.21
S(CCNc1ccc(-n2ccnc2)cc1)c3ccccc3	3.84	12.59	8.21
O=C(OCC1CCC(CC1)Cc2ccccc2)NCCCn3cncc3	0.66	15.85	8.25
O=C(NCCCn1cncc1)c2nnc(o2)-c3csc(c3)-c4ccccc4	0.68	15.85	8.27
O=C(NC[C@@H]1[C@@H]2CCN(C[C@@H]2CO1)c3ccccc3)NCCCn4cncc4	0.76	15.85	8.31
O=C(NCCCn1cncc1)C(=O)NCc2ccc(-c3ccccc3)cc2	0.78	15.85	8.31
N1(CCC2(CC(C2)CCCn3cncc3)CC1)c4ccccc4	0.78	15.85	8.31
O=C(NCCCn1cncc1)/C=C/c2ccc(o2)/C=C/c3ccccc3	0.78	15.85	8.32
O=C(NCCCn1cncc1)c2cc(N3CCN(CC3)c4ccccc4)ncn2	0.79	15.85	8.32
[NH+]1(CCN(CC1)c2ccc(c[nH+]2)-c3ccccc3)Cn4cncc4	0.89	15.85	8.37
O=C(NCCCn1cncc1)NCC[C@@H]2CCO[C@H](C2)c3ccccc3	0.91	15.85	8.38
[n+]1(CCCC#Cc2ccccc2)cccc(NCCCn3cncc3)c1	0.91	15.85	8.38
[n+]1(C[C@@H]2[C@H](C2)Cc3ccccc3)ccc(NCCCn4cncc4)cc1	0.91	15.85	8.38
O=C(NCCCn1cncc1)CSc2nc(Cc3ccccc3)cs2	6.80	10.00	8.40
O=C(NCC1CCC(Oc2ccccc2)CC1)NCCCn3cncc3	1.03	15.85	8.44
[NH+]1(C2CCC(n3cncc3)CC2)CCN(CC1)Cc4ccccc4	1.03	15.85	8.44
O=C(NCC1CCN(CC1)c2ccccc2)C(=O)NCCCn3cncc3	1.18	15.85	8.51
O=C(NCC1CCN(CC1)c2ccccc2)/C=C/n3ccnc3	1.31	15.85	8.58
O=C(NCCCn1cncc1)NCc2ccc(N(c3ccccc3)C)cc2	1.42	15.85	8.63
O=C(NCCCn1cncc1)NCOCCOc2ccccc2	10.99	6.31	8.65
Clc1c(Oc2ccccc2)ccc(CNC(=O)NCCCn3cncc3)c1	1.49	15.85	8.67
[NH2+](C[C@@H]1CCc2nnc(n2C1)NCCCn3cncc3)Cc4ccccc4	1.72	15.85	8.78
O=C(NCCCn1cncc1)Nc2cncc(SCc3ccccc3)c2	1.88	15.85	8.87
O=C(N[C@H](C)c1nnc(Sc2ccccc2)o1)NCCCn3cncc3	1.92	15.85	8.88

O=C(N1CC[C@H](C1)CCOc2ccccc2)NCCCn3cncc3	2.00	15.85	8.92
O=C(OCc1csc(n1)Cc2ccccc2)NCCCn3cncc3	5.54	12.59	9.07
O=C(NCCCn1cncc1)c2nnc(s2)CCCc3ccccc3	2.47	15.85	9.16
S(CCCc1ccccc1)c2ccc(-n3ccnc3)cc2	10.61	7.94	9.27
O=C(NCCCn1cncc1)CCn2cc(nn2)Cc3ccccc3	2.77	15.85	9.31
n1(-c2ccc(CCCCc3ccccc3)cc2)cncc1	2.91	15.85	9.38
O=C(NC(=O)NCCCn1cncc1)CSc2nnc(o2)-c3ccccc3	6.18	12.59	9.38
O=C(NCCCn1cncc1)Nc2ncc(s2)SCc3ccccc3	3.22	15.85	9.54
O=C(Oc1ccccc1)Cn2cc(nn2)C(=O)NCCCn3cncc3	3.62	15.85	9.74
O=C(OCC[NH+](/[N+]([O-])=N/c1ccccc1)C)NCCCn2cncc2	3.72	15.85	9.79
O=C(Nc1nnc(s1)-n2cncc2)c3cccc(-c4ccccc4)c3	0.05	19.95	10.00

Table S2. ECPF4 fingerprint similarity matrix values.

Title	672	924	738	877	621	788	937	563	696	762	599	843	932	719	741	297	328	268	166	103	183	49	411	368	146	41	223	393	442	178	111	162	659	92	271	756	330	247	97	8	6	7	2	1	987	918	714	586	861	781
672	0	0.431	0.408	0.26	0.319	0.273	0.319	0.284	0.27	0.408	0.26	0.313	0.288	0.275	0.358	0.31	0.282	0.293	0.277	0.32	0.259	0.282	0.299	0.321	0.244	0.238	0.291	0.256	0.238	0.293	0.247	0.239	0.288	0.275	0.282	0.371	0.3	0.284	0.233	0.23	0.227	0.235	0.227	0.233	0.293	0.256	0.312	0.338	0.244	0.231
924	0.431	0	0.192	0.286	0.355	0.319	0.275	0.313	0.243	0.292	0.343	0.328	0.299	0.246	0.313	0.246	0.224	0.233	0.207	0.26	0.205	0.24	0.24	0.231	0.188	0.183	0.25	0.215	0.183	0.179	0.205	0.172	0.279	0.188	0.192	0.203	0.183	0.244	0.179	0.176	0.174	0.181	0.174	0.179	0.216	0.23	0.253	0.222	0.188	0.236
738	0.408	0.192	0	0.228	0.247	0.225	0.234	0.25	0.237	0.316	0.228	0.275	0.253	0.239	0.247	0.31	0.266	0.276	0.277	0.286	0.259	0.266	0.266	0.287	0.291	0.253	0.275	0.272	0.268	0.309	0.262	0.267	0.232	0.325	0.299	0.333	0.3	0.253	0.262	0.289	0.286	0.28	0.286	0.293	0.26	0.225	0.247	0.319	0.308	0.231
877	0.26	0.286	0.228	0	0.492	0.484	0.353	0.46	0.282	0.347	0.27	0.349	0.3	0.288	0.313	0.304	0.293	0.288	0.272	0.315	0.268	0.347	0.293	0.282	0.222	0.262	0.269	0.22	0.232	0.226	0.241	0.218	0.262	0.222	0.293	0.274	0.217	0.262	0.212	0.238	0.235	0.244	0.235	0.241	0.27	0.267	0.256	0.243	0.269	0.208
621	0.319	0.355	0.247	0.492	0	0.459	0.483	0.483	0.308	0.379	0.294	0.386	0.349	0.317	0.414	0.355	0.338	0.333	0.311	0.406	0.307	0.338	0.319	0.306	0.24	0.25	0.292	0.237	0.25	0.244	0.26	0.235	0.31	0.24	0.264	0.261	0.234	0.25	0.228	0.256	0.253	0.263	0.253	0.276	0.257	0.328	0.243	0.246	0.257	0.243
788	0.273	0.319	0.225	0.484	0.459	0	0.329	0.453	0.278	0.342	0.319	0.303	0.278	0.246	0.29	0.282	0.273	0.267	0.253	0.293	0.265	0.273	0.307	0.295	0.22	0.229	0.316	0.247	0.229	0.224	0.237	0.216	0.258	0.22	0.256	0.237	0.214	0.275	0.209	0.235	0.233	0.241	0.233	0.238	0.284	0.315	0.253	0.224	0.25	0.205
937	0.319	0.275	0.234	0.353	0.483	0.329	0	0.364	0.309	0.267	0.227	0.297	0.236	0.297	0.365	0.294	0.284	0.278	0.262	0.343	0.275	0.319	0.267	0.256	0.228	0.253	0.276	0.225	0.237	0.232	0.247	0.224	0.25	0.228	0.319	0.264	0.222	0.269	0.217	0.244	0.241	0.25	0.241	0.247	0.373	0.388	0.263	0.268	0.276	0.282
563	0.284	0.313	0.25	0.46	0.483	0.453	0.364	0	0.648	0.532	0.508	0.339	0.309	0.277	0.344	0.313	0.284	0.296	0.278	0.362	0.325	0.301	0.284	0.273	0.244	0.222	0.293	0.225	0.237	0.232	0.231	0.224	0.27	0.228	0.301	0.247	0.222	0.286	0.232	0.244	0.241	0.235	0.241	0.247	0.243	0.257	0.247	0.233	0.293	0.3
696	0.27	0.243	0.237	0.282	0.308	0.278	0.309	0.648	0	0.403	0.422	0.262	0.239	0.281	0.328	0.299	0.27	0.282	0.266	0.329	0.295	0.27	0.27	0.26	0.247	0.21	0.263	0.213	0.225	0.22	0.218	0.212	0.274	0.215	0.253	0.233	0.21	0.241	0.22	0.217	0.214	0.207	0.214	0.22	0.23	0.227	0.234	0.236	0.247	0.286
762	0.408	0.292	0.316	0.347	0.379	0.342	0.267	0.532	0.403	0	0.386	0.419	0.382	0.294	0.3	0.31	0.282	0.293	0.293	0.338	0.305	0.282	0.282	0.304	0.259	0.253	0.291	0.241	0.253	0.309	0.247	0.253	0.269	0.275	0.316	0.412	0.3	0.253	0.247	0.244	0.241	0.235	0.241	0.247	0.26	0.225	0.278	0.267	0.259	0.297
599	0.26	0.343	0.228	0.27	0.294	0.319	0.227	0.508	0.422	0.386	0	0.328	0.3	0.25	0.257	0.25	0.228	0.237	0.226	0.28	0.253	0.228	0.244	0.235	0.222	0.188	0.303	0.282	0.202	0.198	0.195	0.191	0.281	0.193	0.198	0.208	0.188	0.232	0.198	0.195	0.193	0.186	0.193	0.198	0.205	0.25	0.241	0.243	0.207	0.329
843	0.313	0.328	0.275	0.349	0.386	0.303	0.297	0.339	0.262	0.419	0.328	0	0.708	0.49	0.317	0.35	0.313	0.328	0.306	0.318	0.267	0.354	0.313	0.319	0.233	0.278	0.268	0.23	0.227	0.253	0.236	0.244	0.377	0.304	0.375	0.4	0.26	0.227	0.221	0.218	0.215	0.224	0.215	0.221	0.269	0.211	0.29	0.361	0.25	0.235
932	0.288	0.299	0.253	0.3	0.349	0.278	0.236	0.309	0.239	0.382	0.3	0.708	0	0.344	0.288	0.318	0.288	0.3	0.282	0.292	0.247	0.288	0.288	0.293	0.215	0.225	0.247	0.213	0.21	0.235	0.218	0.226	0.339	0.28	0.288	0.304	0.241	0.21	0.205	0.202	0.2	0.207	0.2	0.205	0.213	0.195	0.234	0.271	0.231	0.216
719	0.275	0.246	0.239	0.288	0.317	0.246	0.297	0.277	0.281	0.294	0.25	0.49	0.344	0	0.317	0.35	0.313	0.349	0.288	0.318	0.267	0.354	0.313	0.3	0.233	0.296	0.268	0.23	0.227	0.221	0.236	0.213	0.377	0.233	0.313	0.333	0.243	0.227	0.237	0.218	0.215	0.224	0.215	0.221	0.25	0.211	0.29	0.361	0.216	0.217
741	0.358	0.313	0.247	0.313	0.414	0.29	0.365	0.344	0.328	0.3	0.257	0.317	0.288	0.317	0	0.355	0.319	0.333	0.293	0.364	0.273	0.319	0.319	0.306	0.24	0.234	0.274	0.237	0.25	0.228	0.26	0.22	0.31	0.257	0.247	0.261	0.234	0.25	0.228	0.225	0.222	0.231	0.222	0.228	0.275	0.271	0.278	0.229	0.24	0.243
297	0.31	0.246	0.31	0.304	0.355	0.282	0.294	0.313	0.299	0.31	0.25	0.35	0.318	0.35	0.355	0	0.722	0.607	0.597	0.586	0.493	0.576	0.576	0.548	0.532	0.448	0.508	0.524	0.47	0.456	0.492	0.457	0.5	0.484	0.476	0.435	0.448	0.448	0.435	0.449	0.443	0.463	0.443	0.456	0.25	0.247	0.343	0.257	0.301	0.219
328	0.282	0.224	0.266	0.293	0.338	0.273	0.284	0.284	0.27	0.282	0.228	0.313	0.288	0.313	0.319	0.722	0	0.54	0.606	0.547	0.466	0.515	0.515	0.515	0.478	0.465	0.457	0.471	0.486	0.472	0.507	0.473	0.441	0.417	0.429	0.391	0.405	0.444	0.395	0.427	0.421	0.458	0.421	0.413	0.244	0.241	0.312	0.25	0.275	0.215
268	0.293	0.233	0.276	0.288	0.333	0.267	0.278	0.296	0.282	0.293	0.237	0.328	0.3	0.349	0.333	0.607	0.54	0	0.493	0.548	0.465	0.54	0.54	0.515	0.435	0.53	0.478	0.429	0.423	0.411	0.485	0.395	0.464	0.435	0.448	0.409	0.464	0.423	0.431	0.405	0.4	0.417	0.4	0.411	0.237	0.25	0.307	0.243	0.286	0.224
166	0.277	0.207	0.277	0.272	0.311	0.253	0.262	0.278	0.266	0.293	0.226	0.306	0.282	0.288	0.293	0.597	0.606	0.493	0	0.522	0.449	0.472	0.472	0.473	0.459	0.41	0.44	0.434	0.429	0.455	0.427	0.494	0.4	0.403	0.413	0.378	0.392	0.375	0.383	0.412	0.39	0.387	0.39	0.4	0.226	0.224	0.274	0.247	0.286	0.214
103	0.32	0.26	0.286	0.315	0.406	0.293	0.343	0.362	0.329	0.338	0.28	0.318	0.292	0.318	0.364	0.586	0.547	0.548	0.522	0	0.582	0.571	0.523	0.5	0.464	0.431	0.53	0.417	0.451	0.438	0.449	0.421	0.448	0.423	0.478	0.397	0.411	0.451	0.419	0.472	0.466	0.465	0.466	0.479	0.231	0.26	0.299	0.27	0.312	0.301
183	0.259	0.205	0.259	0.268	0.307	0.265	0.275	0.325	0.295	0.305	0.253	0.267	0.247	0.267	0.273	0.493	0.466	0.465	0.449	0.582	0	0.529	0.446	0.429	0.397	0.387	0.493	0.375	0.405	0.395	0.403	0.381	0.373	0.38	0.427	0.355	0.37	0.405	0.378	0.425	0.42	0.418	0.42	0.43	0.209	0.25	0.271	0.259	0.282	0.241
49	0.282	0.24	0.266	0.347	0.338	0.273	0.319	0.301	0.27	0.282	0.228	0.354	0.288	0.354	0.319	0.576	0.515	0.54	0.472	0.571	0.529	0	0.515	0.493	0.417	0.425	0.5	0.411	0.405	0.395	0.423	0.38	0.441	0.417	0.493	0.412	0.405	0.444	0.395	0.427	0.421	0.438	0.421	0.432	0.26	0.256	0.312	0.267	0.291	0.2
411	0.299	0.24	0.266	0.293	0.319	0.307	0.267	0.284	0.27	0.282	0.244	0.313	0.288	0.313	0.319	0.576	0.515	0.54	0.472	0.523	0.446	0.515	0	0.689	0.417	0.405	0.478	0.431	0.405	0.395	0.423	0.38	0.491	0.437	0.429	0.391	0.405	0.425	0.395	0.39	0.385	0.4	0.385	0.395	0.276	0.241	0.329	0.234	0.275	0.2
368	0.321	0.231	0.287	0.282	0.306	0.295	0.256	0.273	0.26	0.304	0.235	0.319	0.293	0.3	0.306	0.548	0.515	0.515	0.473	0.5	0.429	0.493	0.689	0	0.4	0.39	0.458	0.413	0.39	0.434	0.405	0.383	0.443	0.419	0.431	0.435	0.446	0.427	0.38	0.392	0.387	0.403	0.387	0.38	0.389	0.232	0.3	0.225	0.28	0.193
146	0.244	0.188	0.291	0.222	0.24	0.22	0.228	0.244	0.247	0.259	0.222	0.233	0.215	0.233	0.24	0.532	0.478	0.435	0.459	0.464	0.397	0.417	0.417	0.4	0	0.395	0.444	0.5	0.452	0.44	0.411	0.461	0.338	0.405	0.417	0.38	0.395	0.395	0.479	0.397	0.41	0.39	0.41	0.421	0.222	0.22	0.287	0.228	0.284	0.225
41	0.238	0.183	0.253	0.262	0.25	0.229	0.253	0.222	0.21	0.253	0.188	0.278	0.225	0.296	0.234	0.448	0.465	0.53	0.41	0.431	0.387	0.425	0.405	0.39	0.395	0	0.395	0.39	0.479	0.429	0.458	0.412	0.328	0.395	0.486	0.449	0.479	0.385	0.392	0.405	0.4	0.416	0.383	0.392	0.232	0.229	0.364	0.237	0.262	0.205
223	0.291	0.25	0.275	0.269	0.292	0.316	0.276	0.293	0.263	0.291	0.303	0.268	0.247	0.268	0.274	0.508	0.457	0.478	0.44	0.53	0.493	0.5	0.478	0.458	0.444	0.395	0	0.438	0.413	0.403	0.411	0.387	0.403	0.405	0.457	0.38	0.395	0.472	0.403	0.434	0.429	0.427	0.429	0.44	0.237	0.299	0.321	0.293	0.3	0.21
393	0.256	0.215	0.272	0.22	0.237	0.247	0.225	0.225	0.213	0.241	0.282	0.23	0.213	0.23	0.237	0.524	0.471	0.429	0.434	0.417	0.375	0.411	0.431	0.413	0.5	0.39	0.438	0	0.39	0.38	0.405	0.4	0.354	0.4	0.411	0.375	0.39	0.427	0.38	0.375	0.37	0.385	0.37	0.38	0.235	0.247	0.316	0.195	0.265	0.207
442	0.238	0.183	0.268	0.232	0.25	0.229	0.237	0.237	0.225	0.253	0.202	0.227	0.21	0.227	0.25	0.47	0.486	0.423	0.429	0.451	0.405	0.405	0.405	0.39	0.452	0.479	0.413	0.39	0	0.667	0.641	0.569	0.328	0.472	0.405	0.37	0.403	0.385	0.429	0.423	0.455	0.416	0.436	0.447	0.217	0.229	0.313	0.222	0.277	0.22
178	0.293	0.179	0.309	0.226	0.244	0.224	0.232	0.232	0.22	0.309	0.198	0.253	0.235	0.221	0.228	0.456	0.472	0.411	0.455	0.438	0.395	0.395	0.395	0.434	0.44	0.429	0.403	0.38	0.667	0	0.486	0.597	0.319	0.421	0.432	0.478	0.486	0.375	0.418	0.395	0.425	0.387	0.425	0.436	0.241	0.224	0.289	0.217	0.286	0.214
111	0.247	0.205	0.262	0.241	0.26	0.237	0.247	0.231	0.218	0.247	0.195	0.236	0.218	0.236	0.26	0.492	0.507	0.485	0.427	0.449	0.403	0.423	0.423	0.405	0.411	0.458	0.411	0.405	0.641	0.486	0	0.429	0.344	0.493	0.423	0.386	0.419	0.4	0.39	0.403	0.397	0.413	0.397	0.408	0.225	0.269	0.308	0.215	0.272	0.244
162	0.239	0.172	0.267	0.218	0.235	0.216	0.224	0.224	0.212	0.253	0.191	0.244	0.226	0.213	0.22	0.457	0.473	0.395	0.494	0.421	0.381	0.38	0.38	0.383	0.461	0.412	0.387	0.4	0.569	0.597	0.429	0	0.306	0.423	0.397	0.364	0.378	0.361	0.402	0.398	0.41	0.373	0.427	0.438	0.218	0.216	0.264	0.209	0.321	0.207
659	0.288	0.279	0.232	0.262	0.31	0.258	0.25	0.27	0.274	0.269	0.281	0.377	0.339	0.377	0.31	0.5	0.441	0.464	0.4	0.448	0.373	0.441	0.491	0.443	0.338	0.328	0.403	0.354	0.328	0.319	0.344	0.306	0	0.338	0.349	0.35	0.328	0.348	0.319	0.314	0.31	0.324	0.31	0.319	0.242	0.221	0.303	0.29	0.261	0.227
92	0.275	0.188	0.325	0.222	0.24	0.22	0.228	0.228	0.215	0.275	0.193	0.304	0.28	0.233	0.257	0.484	0.417	0.435	0.403	0.423	0.38	0.417	0.437	0.419	0.405	0.395	0.405	0.4	0.472	0.421	0.493	0.423	0.338	0	0.457	0.42	0.472	0.395	0.385	0.416	0.41	0.427	0.41	0.421	0.237	0.22	0.304	0.198	0.316	0.195
271	0.282	0.192	0.299	0.293	0.264	0.256	0.319	0.301	0.253	0.316	0.198	0.375	0.288	0.313	0.247	0.476	0.429	0.448	0.413	0.478	0.427	0.493	0.429	0.431	0.417	0.486	0.457	0.411	0.405	0.432	0.423	0.397	0.349	0.457	0	0.574	0.444	0.486	0.395	0.427	0.421	0.438	0.421	0.432	0.26	0.241	0.365	0.25	0.342	0.2
756	0.371	0.203	0.333	0.274	0.261	0.237	0.264	0.247	0.233	0.412	0.208	0.4	0.304	0.333	0.261	0.435	0.391	0.409	0.378	0.397	0.355	0.412	0.391	0.435	0.38	0.449	0.38	0.375	0.37	0.478	0.386	0.364	0.35	0.42	0.574	0	0.538	0.37	0.36	0.355	0.351	0.365	0.351	0.36	0.292	0.237	0.386	0.264	0.307	0.211
330	0.3	0.183	0.3	0.217	0.234	0.214	0.222	0.222	0.21	0.3	0.188	0.26	0.241	0.243	0.234	0.448	0.405	0.464	0.392	0.411	0.37	0.405	0.405	0.446	0.395	0.479	0.395	0.39	0.403	0.486	0.419	0.378	0.328	0.472	0.444	0.538	0	0.385	0.392	0.37	0.366	0.38	0.366	0.375	0.247	0.214	0.28	0.193	0.277	0.19
247	0.284	0.244	0.253	0.262	0.25	0.275	0.269	0.286	0.241	0.253	0.232	0.227	0.21	0.227	0.25	0.448	0.444	0.423	0.375	0.451	0.405	0.444	0.425	0.427	0.395	0.385	0.472	0.427	0.385	0.375	0.4	0.361	0.348	0.395	0.486	0.37	0.385	0	0.375	0.423	0.418	0.453	0.418	0.41	0.247	0.259	0.346	0.193	0.325	0.205
97	0.233	0.179	0.262	0.212	0.228	0.209	0.217	0.232	0.22	0.247	0.198	0.221	0.205	0.237	0.228	0.435	0.395	0.431	0.383	0.419	0.378	0.395	0.395	0.38	0.479	0.392	0.403	0.38	0.429	0.418	0.39	0.402	0.319	0.385	0.395	0.36	0.392	0.375	0	0.378	0.39	0.37	0.39	0.4	0.212	0.209	0.274	0.202	0.271	0.2
8	0.23	0.176	0.289	0.238	0.256	0.235	0.244	0.244	0.217	0.244	0.195	0.218	0.202	0.218	0.225	0.449	0.427	0.405	0.412	0.472	0.425	0.427	0.39	0.392	0.397	0.405	0.434	0.375	0.423	0.395	0.403	0.398	0.314	0.416	0.427	0.355	0.37	0.423	0.378	0	0.797	0.75	0.597	0.487	0.224	0.235	0.286	0.229	0.282	0.212
6	0.227	0.174	0.286	0.235	0.253	0.233	0.241	0.241	0.214	0.241	0.193	0.215	0.2	0.215	0.222	0.443	0.421	0.4	0.39	0.466	0.42	0.421	0.385	0.387	0.41	0.4	0.429	0.37	0.455	0.425	0.397	0.41	0.31	0.41	0.421	0.351	0.366	0.418	0.39	0.797	0	0.794	0.681	0.541	0.221	0.233	0.282	0.226	0.279	0.209
7	0.235	0.181	0.28	0.244	0.263	0.241	0.25	0.235	0.207	0.235	0.186	0.224	0.207	0.224	0.231	0.463	0.458	0.417	0.387	0.465	0.418	0.438	0.4	0.403	0.39	0.416	0.427	0.385	0.416	0.387	0.413	0.373	0.324	0.427	0.438	0.365	0.38	0.453	0.37	0.75	0.794	0	0.614	0.48	0.229	0.241	0.293	0.22	0.274	0.202
2	0.227	0.174	0.286	0.235	0.253	0.233	0.241	0.241	0.214	0.241	0.193	0.215	0.2	0.215	0.222	0.443	0.421	0.4	0.39	0.466	0.42	0.421	0.385	0.387	0.41	0.383	0.429	0.37	0.436	0.425	0.397	0.427	0.31	0.41	0.421	0.351	0.366	0.418	0.39	0.597	0.681	0.614	0	0.676	0.221	0.233	0.267	0.226	0.279	0.209
1	0.233	0.179	0.293	0.241	0.276	0.238	0.247	0.247	0.22	0.247	0.198	0.221	0.205	0.221	0.228	0.456	0.413	0.411	0.4	0.479	0.43	0.432	0.395	0.38	0.421	0.392	0.44	0.38	0.447	0.436	0.408	0.438	0.319	0.421	0.432	0.36	0.375	0.41	0.4	0.487	0.541	0.48	0.676	0	0.212	0.238	0.274	0.232	0.286	0.214
987	0.293	0.216	0.26	0.27	0.257	0.284	0.373	0.243	0.23	0.26	0.205	0.269	0.213	0.25	0.275	0.25	0.244	0.237	0.226	0.231	0.209	0.26	0.276	0.389	0.222	0.232	0.237	0.235	0.217	0.241	0.225	0.218	0.242	0.237	0.26	0.292	0.247	0.247	0.212	0.224	0.221	0.229	0.221	0.212	0	0.319	0.273	0.211	0.237	0.24
918	0.256	0.23	0.225	0.267	0.328	0.315	0.388	0.257	0.227	0.225	0.25	0.211	0.195	0.211	0.271	0.247	0.241	0.25	0.224	0.26	0.25	0.256	0.241	0.232	0.22	0.229	0.299	0.247	0.229	0.224	0.269	0.216	0.221	0.22	0.241	0.237	0.214	0.259	0.209	0.235	0.233	0.241	0.233	0.238	0.319	0	0.269	0.192	0.235	0.288
714	0.312	0.253	0.247	0.256	0.243	0.253	0.263	0.247	0.234	0.278	0.241	0.29	0.234	0.29	0.278	0.343	0.312	0.307	0.274	0.299	0.271	0.312	0.329	0.3	0.287	0.364	0.321	0.316	0.313	0.289	0.308	0.264	0.303	0.304	0.365	0.386	0.28	0.346	0.274	0.286	0.282	0.293	0.267	0.274	0.273	0.269	0	0.231	0.272	0.213
586	0.338	0.222	0.319	0.243	0.246	0.224	0.268	0.233	0.236	0.267	0.243	0.361	0.271	0.361	0.229	0.257	0.25	0.243	0.247	0.27	0.259	0.267	0.234	0.225	0.228	0.237	0.293	0.195	0.222	0.217	0.215	0.209	0.29	0.198	0.25	0.264	0.193	0.193	0.202	0.229	0.226	0.22	0.226	0.232	0.211	0.192	0.231	0	0.198	0.23
861	0.244	0.188	0.308	0.269	0.257	0.25	0.276	0.293	0.247	0.259	0.207	0.25	0.231	0.216	0.24	0.301	0.275	0.286	0.286	0.312	0.282	0.291	0.275	0.28	0.284	0.262	0.3	0.265	0.277	0.286	0.272	0.321	0.261	0.316	0.342	0.307	0.277	0.325	0.271	0.282	0.279	0.274	0.279	0.286	0.237	0.235	0.272	0.198	0	0.21
781	0.231	0.236	0.231	0.208	0.243	0.205	0.282	0.3	0.286	0.297	0.329	0.235	0.216	0.217	0.243	0.219	0.215	0.224	0.214	0.301	0.241	0.2	0.2	0.193	0.225	0.205	0.21	0.207	0.22	0.214	0.244	0.207	0.227	0.195	0.2	0.211	0.19	0.205	0.2	0.212	0.209	0.202	0.209	0.214	0.24	0.288	0.213	0.23	0.21	0

Table S3. ECPF6 fingerprint similarity matrix values.

Title	621	877	788	696	563	762	599	741	49	103	328	297	268	166	368	411	183	146	393	659	41	223	97	756	271	247	178	442	162	111	92	330	1	2	7	6	8	932	843	719	937	714	924	672	738	987	586	918	861	781
621	0	0.385	0.351	0.222	0.371	0.296	0.225	0.356	0.275	0.344	0.252	0.287	0.276	0.232	0.238	0.245	0.221	0.168	0.168	0.267	0.172	0.209	0.164	0.183	0.183	0.177	0.169	0.172	0.164	0.177	0.17	0.165	0.19	0.176	0.183	0.178	0.179	0.28	0.329	0.27	0.326	0.173	0.26	0.233	0.176	0.186	0.181	0.223	0.183	0.17
877	0.385	0	0.378	0.206	0.368	0.274	0.209	0.235	0.255	0.234	0.223	0.229	0.22	0.207	0.221	0.227	0.197	0.157	0.157	0.206	0.18	0.195	0.153	0.191	0.202	0.185	0.159	0.161	0.154	0.165	0.158	0.154	0.168	0.165	0.171	0.167	0.168	0.245	0.274	0.222	0.25	0.181	0.217	0.195	0.164	0.194	0.179	0.186	0.191	0.148
788	0.351	0.378	0	0.204	0.365	0.271	0.241	0.21	0.198	0.211	0.2	0.204	0.196	0.185	0.219	0.225	0.195	0.156	0.175	0.192	0.16	0.224	0.152	0.168	0.179	0.193	0.157	0.16	0.153	0.164	0.157	0.153	0.167	0.164	0.169	0.165	0.167	0.219	0.232	0.184	0.236	0.179	0.238	0.204	0.162	0.204	0.167	0.216	0.179	0.147
696	0.222	0.206	0.204	0	0.506	0.287	0.347	0.235	0.198	0.234	0.2	0.216	0.208	0.195	0.198	0.204	0.216	0.174	0.154	0.204	0.149	0.193	0.16	0.167	0.179	0.172	0.156	0.158	0.151	0.153	0.155	0.151	0.156	0.153	0.149	0.154	0.156	0.184	0.196	0.208	0.225	0.168	0.181	0.204	0.171	0.17	0.176	0.162	0.179	0.198
563	0.371	0.368	0.365	0.506	0	0.424	0.387	0.245	0.218	0.255	0.209	0.225	0.217	0.203	0.207	0.213	0.235	0.172	0.162	0.202	0.157	0.212	0.168	0.176	0.209	0.202	0.164	0.167	0.159	0.161	0.164	0.16	0.174	0.171	0.167	0.172	0.174	0.242	0.258	0.206	0.26	0.177	0.238	0.213	0.18	0.179	0.174	0.182	0.209	0.208
762	0.296	0.274	0.271	0.287	0.424	0	0.288	0.221	0.209	0.243	0.211	0.226	0.218	0.215	0.23	0.214	0.225	0.185	0.175	0.204	0.179	0.214	0.18	0.294	0.221	0.183	0.215	0.179	0.18	0.174	0.197	0.212	0.176	0.173	0.169	0.175	0.176	0.293	0.312	0.22	0.202	0.2	0.226	0.295	0.225	0.193	0.198	0.164	0.19	0.209
599	0.225	0.209	0.241	0.347	0.387	0.288	0	0.19	0.171	0.204	0.172	0.185	0.179	0.169	0.181	0.186	0.189	0.158	0.198	0.208	0.135	0.217	0.145	0.15	0.143	0.167	0.142	0.144	0.137	0.138	0.14	0.137	0.142	0.14	0.135	0.141	0.142	0.235	0.25	0.188	0.173	0.172	0.255	0.196	0.165	0.153	0.18	0.177	0.153	0.224
741	0.356	0.235	0.21	0.235	0.245	0.221	0.19	0	0.262	0.316	0.24	0.287	0.276	0.221	0.238	0.245	0.2	0.168	0.168	0.267	0.162	0.198	0.164	0.183	0.173	0.177	0.16	0.172	0.154	0.177	0.18	0.165	0.16	0.157	0.162	0.158	0.16	0.227	0.27	0.27	0.258	0.194	0.222	0.257	0.176	0.198	0.17	0.189	0.173	0.17
49	0.275	0.255	0.198	0.198	0.218	0.209	0.171	0.262	0	0.5	0.424	0.494	0.473	0.389	0.42	0.433	0.442	0.333	0.333	0.391	0.333	0.408	0.324	0.298	0.396	0.368	0.316	0.321	0.305	0.33	0.336	0.327	0.351	0.345	0.358	0.348	0.351	0.224	0.302	0.302	0.229	0.226	0.177	0.209	0.188	0.188	0.193	0.179	0.216	0.143
103	0.344	0.234	0.211	0.234	0.255	0.243	0.204	0.316	0.5	0	0.443	0.5	0.478	0.419	0.424	0.438	0.461	0.362	0.336	0.395	0.336	0.426	0.339	0.288	0.386	0.371	0.342	0.349	0.33	0.346	0.34	0.33	0.38	0.373	0.374	0.376	0.38	0.226	0.265	0.265	0.243	0.217	0.189	0.232	0.2	0.168	0.195	0.181	0.228	0.205
328	0.252	0.223	0.2	0.2	0.209	0.211	0.172	0.24	0.424	0.443	0	0.63	0.447	0.52	0.439	0.438	0.38	0.402	0.402	0.364	0.361	0.371	0.327	0.288	0.346	0.358	0.367	0.374	0.378	0.385	0.34	0.33	0.33	0.336	0.361	0.339	0.342	0.226	0.24	0.24	0.209	0.228	0.168	0.211	0.19	0.179	0.184	0.171	0.207	0.154
297	0.287	0.229	0.204	0.216	0.225	0.226	0.185	0.287	0.494	0.5	0.63	0	0.524	0.5	0.462	0.477	0.396	0.436	0.436	0.436	0.35	0.402	0.353	0.313	0.375	0.36	0.356	0.363	0.368	0.374	0.381	0.356	0.356	0.349	0.363	0.352	0.356	0.245	0.289	0.289	0.214	0.245	0.181	0.226	0.215	0.181	0.187	0.173	0.222	0.155
268	0.276	0.22	0.196	0.208	0.217	0.218	0.179	0.276	0.473	0.478	0.447	0.524	0	0.408	0.442	0.457	0.381	0.35	0.35	0.415	0.43	0.386	0.352	0.3	0.36	0.346	0.33	0.336	0.319	0.373	0.353	0.369	0.33	0.324	0.336	0.327	0.33	0.235	0.277	0.29	0.206	0.225	0.174	0.218	0.196	0.174	0.18	0.177	0.214	0.159
166	0.232	0.207	0.185	0.195	0.203	0.215	0.169	0.221	0.389	0.419	0.52	0.5	0.408	0	0.402	0.4	0.362	0.382	0.369	0.33	0.322	0.354	0.314	0.277	0.33	0.308	0.35	0.333	0.385	0.33	0.325	0.316	0.317	0.311	0.311	0.314	0.328	0.219	0.231	0.22	0.193	0.202	0.156	0.205	0.195	0.165	0.18	0.159	0.211	0.152
368	0.238	0.221	0.219	0.198	0.207	0.23	0.181	0.238	0.42	0.424	0.439	0.462	0.442	0.402	0	0.616	0.364	0.333	0.346	0.375	0.321	0.381	0.324	0.324	0.356	0.355	0.364	0.321	0.316	0.33	0.349	0.377	0.316	0.322	0.333	0.325	0.327	0.236	0.25	0.238	0.196	0.226	0.177	0.241	0.209	0.279	0.172	0.169	0.216	0.143
411	0.245	0.227	0.225	0.204	0.213	0.214	0.186	0.245	0.433	0.438	0.438	0.477	0.457	0.4	0.616	0	0.374	0.343	0.356	0.405	0.33	0.392	0.333	0.294	0.353	0.352	0.324	0.33	0.313	0.34	0.359	0.336	0.324	0.319	0.33	0.321	0.324	0.231	0.245	0.245	0.202	0.243	0.182	0.225	0.193	0.204	0.177	0.174	0.211	0.147
183	0.221	0.197	0.195	0.216	0.235	0.225	0.189	0.2	0.442	0.461	0.38	0.396	0.381	0.362	0.364	0.374	0	0.322	0.31	0.303	0.311	0.404	0.314	0.265	0.355	0.342	0.317	0.322	0.306	0.319	0.313	0.305	0.35	0.345	0.345	0.347	0.35	0.188	0.198	0.198	0.203	0.202	0.156	0.195	0.185	0.156	0.19	0.177	0.211	0.171
146	0.168	0.157	0.156	0.174	0.172	0.185	0.158	0.168	0.333	0.362	0.402	0.436	0.35	0.382	0.333	0.343	0.322	0	0.431	0.268	0.316	0.349	0.406	0.28	0.336	0.324	0.357	0.364	0.381	0.324	0.33	0.321	0.333	0.328	0.316	0.33	0.322	0.157	0.165	0.165	0.162	0.212	0.134	0.175	0.205	0.164	0.16	0.157	0.212	0.16
393	0.168	0.157	0.175	0.154	0.162	0.175	0.198	0.168	0.333	0.336	0.402	0.436	0.35	0.369	0.346	0.356	0.31	0.431	0	0.281	0.316	0.349	0.319	0.28	0.336	0.349	0.31	0.316	0.333	0.324	0.33	0.321	0.31	0.305	0.316	0.308	0.31	0.157	0.165	0.165	0.162	0.233	0.154	0.185	0.195	0.174	0.14	0.176	0.202	0.15
659	0.267	0.206	0.192	0.204	0.202	0.204	0.208	0.267	0.391	0.395	0.364	0.436	0.415	0.33	0.375	0.405	0.303	0.268	0.281	0	0.257	0.319	0.26	0.267	0.277	0.278	0.252	0.257	0.243	0.265	0.271	0.263	0.252	0.248	0.257	0.25	0.252	0.264	0.316	0.316	0.189	0.224	0.204	0.216	0.168	0.179	0.211	0.158	0.2	0.162
41	0.172	0.18	0.16	0.149	0.157	0.179	0.135	0.162	0.333	0.336	0.361	0.35	0.43	0.322	0.321	0.33	0.311	0.316	0.316	0.257	0	0.313	0.319	0.343	0.4	0.313	0.333	0.363	0.322	0.348	0.319	0.369	0.311	0.306	0.328	0.319	0.322	0.161	0.191	0.202	0.176	0.278	0.13	0.169	0.179	0.168	0.164	0.161	0.195	0.145
223	0.209	0.195	0.224	0.193	0.212	0.214	0.217	0.198	0.408	0.426	0.371	0.402	0.386	0.354	0.381	0.392	0.404	0.349	0.349	0.319	0.313	0	0.327	0.278	0.371	0.383	0.319	0.325	0.308	0.321	0.327	0.319	0.354	0.348	0.348	0.351	0.354	0.186	0.196	0.196	0.202	0.231	0.183	0.214	0.193	0.172	0.219	0.205	0.22	0.149
97	0.164	0.153	0.152	0.16	0.168	0.18	0.145	0.164	0.324	0.339	0.327	0.353	0.352	0.314	0.324	0.333	0.314	0.406	0.319	0.26	0.319	0.327	0	0.273	0.327	0.316	0.348	0.354	0.336	0.316	0.321	0.325	0.325	0.319	0.308	0.322	0.314	0.153	0.161	0.171	0.158	0.207	0.131	0.171	0.19	0.16	0.146	0.153	0.207	0.146
756	0.183	0.191	0.168	0.167	0.176	0.294	0.15	0.183	0.298	0.288	0.288	0.313	0.3	0.277	0.324	0.294	0.265	0.28	0.28	0.267	0.343	0.278	0.273	0	0.441	0.278	0.336	0.27	0.267	0.278	0.308	0.376	0.265	0.261	0.27	0.263	0.265	0.216	0.269	0.229	0.187	0.301	0.145	0.257	0.234	0.212	0.183	0.17	0.229	0.152
271	0.183	0.202	0.179	0.179	0.209	0.221	0.143	0.173	0.396	0.386	0.346	0.375	0.36	0.33	0.356	0.353	0.355	0.336	0.336	0.277	0.4	0.371	0.327	0.441	0	0.398	0.342	0.324	0.319	0.333	0.365	0.355	0.355	0.348	0.361	0.351	0.355	0.204	0.265	0.216	0.22	0.284	0.138	0.2	0.211	0.189	0.174	0.171	0.25	0.144
247	0.177	0.185	0.193	0.172	0.202	0.183	0.167	0.177	0.368	0.371	0.358	0.36	0.346	0.308	0.355	0.352	0.342	0.324	0.349	0.278	0.313	0.383	0.316	0.278	0.398	0	0.308	0.313	0.298	0.321	0.327	0.319	0.342	0.348	0.373	0.351	0.354	0.155	0.164	0.164	0.191	0.252	0.172	0.203	0.183	0.183	0.139	0.185	0.263	0.149
178	0.169	0.159	0.157	0.156	0.164	0.215	0.142	0.16	0.316	0.342	0.367	0.356	0.33	0.35	0.364	0.324	0.317	0.357	0.31	0.252	0.333	0.319	0.348	0.336	0.342	0.308	0	0.576	0.5	0.366	0.336	0.4	0.339	0.333	0.311	0.336	0.317	0.168	0.177	0.157	0.164	0.211	0.128	0.205	0.215	0.175	0.152	0.159	0.211	0.152
442	0.172	0.161	0.16	0.158	0.167	0.179	0.144	0.172	0.321	0.349	0.374	0.363	0.336	0.333	0.321	0.33	0.322	0.364	0.316	0.257	0.363	0.325	0.354	0.27	0.324	0.313	0.576	0	0.481	0.495	0.393	0.322	0.345	0.339	0.328	0.353	0.333	0.151	0.159	0.159	0.167	0.225	0.13	0.169	0.189	0.158	0.154	0.161	0.205	0.154
162	0.164	0.154	0.153	0.151	0.159	0.18	0.137	0.154	0.305	0.33	0.378	0.368	0.319	0.385	0.316	0.313	0.306	0.381	0.333	0.243	0.322	0.308	0.336	0.267	0.319	0.298	0.5	0.481	0	0.331	0.336	0.306	0.339	0.333	0.301	0.325	0.317	0.163	0.171	0.151	0.159	0.195	0.124	0.171	0.189	0.16	0.147	0.154	0.234	0.147
111	0.177	0.165	0.164	0.153	0.161	0.174	0.138	0.177	0.33	0.346	0.385	0.374	0.373	0.33	0.33	0.34	0.319	0.324	0.324	0.265	0.348	0.321	0.316	0.278	0.333	0.321	0.366	0.495	0.331	0	0.404	0.33	0.319	0.314	0.325	0.316	0.319	0.155	0.164	0.164	0.171	0.22	0.143	0.174	0.183	0.162	0.149	0.195	0.2	0.178
92	0.17	0.158	0.157	0.155	0.164	0.197	0.14	0.18	0.336	0.34	0.34	0.381	0.353	0.325	0.349	0.359	0.313	0.33	0.33	0.271	0.319	0.327	0.321	0.308	0.365	0.327	0.336	0.393	0.336	0.404	0	0.374	0.348	0.342	0.355	0.345	0.348	0.2	0.212	0.167	0.164	0.224	0.136	0.197	0.239	0.175	0.142	0.158	0.235	0.142
330	0.165	0.154	0.153	0.151	0.16	0.212	0.137	0.165	0.327	0.33	0.33	0.356	0.369	0.316	0.377	0.336	0.305	0.321	0.321	0.263	0.369	0.319	0.325	0.376	0.355	0.319	0.4	0.322	0.306	0.33	0.374	0	0.305	0.3	0.31	0.303	0.305	0.174	0.183	0.173	0.16	0.208	0.132	0.212	0.212	0.181	0.138	0.154	0.208	0.138
1	0.19	0.168	0.167	0.156	0.174	0.176	0.142	0.16	0.351	0.38	0.33	0.356	0.33	0.317	0.316	0.324	0.35	0.333	0.31	0.252	0.311	0.354	0.325	0.265	0.355	0.342	0.339	0.345	0.339	0.319	0.348	0.305	0	0.584	0.393	0.432	0.398	0.149	0.157	0.157	0.174	0.202	0.128	0.167	0.215	0.156	0.161	0.168	0.211	0.152
2	0.176	0.165	0.164	0.153	0.171	0.173	0.14	0.157	0.345	0.373	0.336	0.349	0.324	0.311	0.322	0.319	0.345	0.328	0.305	0.248	0.306	0.348	0.319	0.261	0.348	0.348	0.333	0.339	0.333	0.314	0.342	0.3	0.584	0	0.491	0.533	0.481	0.146	0.154	0.154	0.171	0.198	0.126	0.164	0.211	0.163	0.159	0.165	0.208	0.15
7	0.183	0.171	0.169	0.149	0.167	0.169	0.135	0.162	0.358	0.374	0.361	0.363	0.336	0.311	0.333	0.33	0.345	0.316	0.316	0.257	0.328	0.348	0.308	0.27	0.361	0.373	0.311	0.328	0.301	0.325	0.355	0.31	0.393	0.491	0	0.744	0.677	0.151	0.159	0.159	0.176	0.215	0.13	0.169	0.208	0.168	0.154	0.171	0.205	0.145
6	0.178	0.167	0.165	0.154	0.172	0.175	0.141	0.158	0.348	0.376	0.339	0.352	0.327	0.314	0.325	0.321	0.347	0.33	0.308	0.25	0.319	0.351	0.322	0.263	0.351	0.351	0.336	0.353	0.325	0.316	0.345	0.303	0.432	0.533	0.744	0	0.71	0.148	0.155	0.155	0.172	0.21	0.127	0.165	0.213	0.164	0.16	0.167	0.21	0.151
8	0.179	0.168	0.167	0.156	0.174	0.176	0.142	0.16	0.351	0.38	0.342	0.356	0.33	0.328	0.327	0.324	0.35	0.322	0.31	0.252	0.322	0.354	0.314	0.265	0.355	0.354	0.317	0.333	0.317	0.319	0.348	0.305	0.398	0.481	0.677	0.71	0	0.149	0.157	0.157	0.174	0.211	0.128	0.167	0.215	0.165	0.161	0.168	0.211	0.152
932	0.28	0.245	0.219	0.184	0.242	0.293	0.235	0.227	0.224	0.226	0.226	0.245	0.235	0.219	0.236	0.231	0.188	0.157	0.157	0.264	0.161	0.186	0.153	0.216	0.204	0.155	0.168	0.151	0.163	0.155	0.2	0.174	0.149	0.146	0.151	0.148	0.149	0	0.606	0.267	0.183	0.171	0.232	0.219	0.185	0.162	0.202	0.144	0.171	0.157
843	0.329	0.274	0.232	0.196	0.258	0.312	0.25	0.27	0.302	0.265	0.24	0.289	0.277	0.231	0.25	0.245	0.198	0.165	0.165	0.316	0.191	0.196	0.161	0.269	0.265	0.164	0.177	0.159	0.171	0.164	0.212	0.183	0.157	0.154	0.159	0.155	0.157	0.606	0	0.44	0.219	0.204	0.247	0.232	0.196	0.196	0.253	0.152	0.181	0.167
719	0.27	0.222	0.184	0.208	0.206	0.22	0.188	0.27	0.302	0.265	0.24	0.289	0.29	0.22	0.238	0.245	0.198	0.165	0.165	0.316	0.202	0.196	0.171	0.229	0.216	0.164	0.157	0.159	0.151	0.164	0.167	0.173	0.157	0.154	0.159	0.155	0.157	0.267	0.44	0	0.219	0.204	0.184	0.208	0.173	0.184	0.253	0.152	0.159	0.155
937	0.326	0.25	0.236	0.225	0.26	0.202	0.173	0.258	0.229	0.243	0.209	0.214	0.206	0.193	0.196	0.202	0.203	0.162	0.162	0.189	0.176	0.202	0.158	0.187	0.22	0.191	0.164	0.167	0.159	0.171	0.164	0.16	0.174	0.171	0.176	0.172	0.174	0.183	0.219	0.219	0	0.188	0.214	0.248	0.17	0.289	0.196	0.275	0.198	0.208
714	0.173	0.181	0.179	0.168	0.177	0.2	0.172	0.194	0.226	0.217	0.228	0.245	0.225	0.202	0.226	0.243	0.202	0.212	0.233	0.224	0.278	0.231	0.207	0.301	0.284	0.252	0.211	0.225	0.195	0.22	0.224	0.208	0.202	0.198	0.215	0.21	0.211	0.171	0.204	0.204	0.188	0	0.179	0.221	0.179	0.2	0.164	0.191	0.207	0.154
924	0.26	0.217	0.238	0.181	0.238	0.226	0.255	0.222	0.177	0.189	0.168	0.181	0.174	0.156	0.177	0.182	0.156	0.134	0.154	0.204	0.13	0.183	0.131	0.145	0.138	0.172	0.128	0.13	0.124	0.143	0.136	0.132	0.128	0.126	0.13	0.127	0.128	0.232	0.247	0.184	0.214	0.179	0	0.354	0.14	0.159	0.165	0.162	0.138	0.165
672	0.233	0.195	0.204	0.204	0.213	0.295	0.196	0.257	0.209	0.232	0.211	0.226	0.218	0.205	0.241	0.225	0.195	0.175	0.185	0.216	0.169	0.214	0.171	0.257	0.2	0.203	0.205	0.169	0.171	0.174	0.197	0.212	0.167	0.164	0.169	0.165	0.167	0.219	0.232	0.208	0.248	0.221	0.354	0	0.32	0.215	0.279	0.184	0.179	0.167
738	0.176	0.164	0.162	0.171	0.18	0.225	0.165	0.176	0.188	0.2	0.19	0.215	0.196	0.195	0.209	0.193	0.185	0.205	0.195	0.168	0.179	0.193	0.19	0.234	0.211	0.183	0.215	0.189	0.189	0.183	0.239	0.212	0.215	0.211	0.208	0.213	0.215	0.185	0.196	0.173	0.17	0.179	0.14	0.32	0	0.193	0.255	0.164	0.221	0.167
987	0.186	0.194	0.204	0.17	0.179	0.193	0.153	0.198	0.188	0.168	0.179	0.181	0.174	0.165	0.279	0.204	0.156	0.164	0.174	0.179	0.168	0.172	0.16	0.212	0.189	0.183	0.175	0.158	0.16	0.162	0.175	0.181	0.156	0.163	0.168	0.164	0.165	0.162	0.196	0.184	0.289	0.2	0.159	0.215	0.193	0	0.155	0.24	0.179	0.187
586	0.181	0.179	0.167	0.176	0.174	0.198	0.18	0.17	0.193	0.195	0.184	0.187	0.18	0.18	0.172	0.177	0.19	0.16	0.14	0.211	0.164	0.219	0.146	0.183	0.174	0.139	0.152	0.154	0.147	0.149	0.142	0.138	0.161	0.159	0.154	0.16	0.161	0.202	0.253	0.253	0.196	0.164	0.165	0.279	0.255	0.155	0	0.138	0.144	0.161
918	0.223	0.186	0.216	0.162	0.182	0.164	0.177	0.189	0.179	0.181	0.171	0.173	0.177	0.159	0.169	0.174	0.177	0.157	0.176	0.158	0.161	0.205	0.153	0.17	0.171	0.185	0.159	0.161	0.154	0.195	0.158	0.154	0.168	0.165	0.171	0.167	0.168	0.144	0.152	0.152	0.275	0.191	0.162	0.184	0.164	0.24	0.138	0	0.171	0.222
861	0.183	0.191	0.179	0.179	0.209	0.19	0.153	0.173	0.216	0.228	0.207	0.222	0.214	0.211	0.216	0.211	0.211	0.212	0.202	0.2	0.195	0.22	0.207	0.229	0.25	0.263	0.211	0.205	0.234	0.2	0.235	0.208	0.211	0.208	0.205	0.21	0.211	0.171	0.181	0.159	0.198	0.207	0.138	0.179	0.221	0.179	0.144	0.171	0	0.154
781	0.17	0.148	0.147	0.198	0.208	0.209	0.224	0.17	0.143	0.205	0.154	0.155	0.159	0.152	0.143	0.147	0.171	0.16	0.15	0.162	0.145	0.149	0.146	0.152	0.144	0.149	0.152	0.154	0.147	0.178	0.142	0.138	0.152	0.15	0.145	0.151	0.152	0.157	0.167	0.155	0.208	0.154	0.165	0.167	0.167	0.187	0.161	0.222	0.154	0
